# Positive selection neighboring functionally essential sites and disease-implicated regions of mammalian reproductive proteins

**DOI:** 10.1186/1471-2148-10-39

**Published:** 2010-02-11

**Authors:** Claire C Morgan, Noeleen B Loughran, Thomas A Walsh, Alan J Harrison, Mary J O'Connell

**Affiliations:** 1 Bioinformatics and Molecular Evolution Group, School of Biotechnology, Dublin City University, Glasnevin, Dublin 9, Ireland

## Abstract

**Background:**

Reproductive proteins are central to the continuation of all mammalian species. The evolution of these proteins has been greatly influenced by environmental pressures induced by pathogens, rival sperm, sexual selection and sexual conflict. Positive selection has been demonstrated in many of these proteins with particular focus on primate lineages. However, the *mammalia *are a diverse group in terms of mating habits, population sizes and germ line generation times. We have examined the selective pressures at work on a number of novel reproductive proteins across a wide variety of *mammalia*.

**Results:**

We show that selective pressures on reproductive proteins are highly varied. Of the 10 genes analyzed in detail, all contain signatures of positive selection either across specific sites or in specific lineages or a combination of both. Our analysis of SP56 and Col1a1 are entirely novel and the results show positively selected sites present in each gene. Our findings for the Col1a1 gene are suggestive of a link between positive selection and severe disease type. We find evidence in our dataset to suggest that interacting proteins are evolving in symphony: most likely to maintain interacting functionality.

**Conclusion:**

Our *in silico *analyses show positively selected sites are occurring near catalytically important regions suggesting selective pressure to maximize efficient fertilization. In those cases where a mechanism of protein function is not fully understood, the sites presented here represent ideal candidates for mutational study. This work has highlighted the widespread rate heterogeneity in mutational rates across the *mammalia *and specifically has shown that the evolution of reproductive proteins is highly varied depending on the species and interacting partners. We have shown that positive selection and disease are closely linked in the Col1a1 gene.

## Background

Reproductive proteins are essential for success of sexually reproducing species and indeed for the emergence of new species. In the past it has been observed that reproductive proteins tend to be under positive selective pressure to change, i.e. adaptive evolution, a trend found in a variety of animal species from abalone to primates [1,2]. Adaptive evolution or positive selection is a selective pressure placed on a protein by a change in environment in order to improve the fitness of the organism in that environment.

With changes in environment, that can include mating system, there is a subsequent selective pressure on the protein sequences related to those functions to adapt accordingly. This variation can be detected using the well-known measurements of the rate of non-synonymous substitutions per non-synonymous site (Dn) and synonymous substitutions per synonymous site (Ds) and their ratio ω = Dn/Ds. The detection of adaptive evolution, where the ratio exceeds unity, is referred to as positive Darwinian selection. Detecting positive Darwinian selection in a region of a protein, or indeed in a lineage of a phylogeny, indicates that there is a selective advantage in changing the amino acid sequence in this region. These signals are essential for our understanding of functionally important residues in a protein sequence and protein functional shift.

In general, the rate of mutation that a gene undergoes is contingent on a number of factors including; protein structure, presence of gene duplicates, location in the genome, effective population size, germ line generation time, and composition of the sequence (for review see [3]). It has recently been shown that the number of physical interactions of a particular protein also influences the intrinsic rate of evolution [4]. Evidence for the generation time effect has come from studies on various proteins and species including analyses of substitution rates in higher primates and rodents [5], substitution rates in higher grasses and in palms [6], in mammalian genomes [7] and in chloroplast and sex mutation rate ratios [5,6]. With recent advances in sequencing we have an opportunity to examine these effects using a wider selection of proteins and species. Documented selective pressures associated with positive selection in reproductive proteins include: (i) intense sperm competition whereby sperm from numerous males, ejaculated into the female reproductive tract, compete with one another for the prized fertilization of the egg [8]; (ii) evasion of the immune system, whereby surface layer reproductive proteins evolve to evade destruction by the host's immune system [8]; and finally (iii) selective pressures enforced by mating system, related of course to point (i) above. Species that are more promiscuous have increased levels of selective pressure acting on reproductive proteins than species that are monogamous. This later point is illustrated in the study of SEMG2, where adaptive evolution was found to correlate with mating system in primates [9].

In order to determine the variation in selective pressure in these proteins, there are a number of criteria that the data must meet. Firstly, the data must have a robust phylogenetic signal. Secondly, systematic biases that may exist in the data must be minimized, these include but are not limited to: long branch attraction (LBA), amino acid composition bias, base composition bias and unqualified ortholog predictions, all of which may lead to inaccurate estimates of phylogeny. Thirdly, sensitivity to taxa number is a known limitation of methods for detecting positive selection, therefore more than 6 taxa are needed to gain accurate estimations of selective pressure using the maximum likelihood (ML) method applied here [10].

In this study we have selected a subset of proteins that have roles to play in reproduction. Our dataset was composed of three major datatypes, (i) previously published reproductive proteins, (ii) interacting proteins, here we identified proteins shown to interact with (i), and finally (iii), genes identified from microarray experiments as being highly expressed in reproductive tissues. For group (iii) we assume that those proteins highly expressed in reproductive tissues are important for the function of that tissue. The previously untested reproductive proteins analysed here are from data types (ii) and (iii) outlined above. These novel proteins are SP56, Porimin and Col1a1. SP56 is sperm binding protein number 56, this protein is a representative of the interacting protein subset of sequences analysed. SP56 has been shown to interact with ZP3 - a well-studied reproductive protein. Both Porimin and Col1a1 have been identified from published microarray experiments on normal human tissue [11], and were selected for analysis due to their high levels of expression in reproductive tissues in that study. Porimin is a transmembrane protein that is highly expressed in the uterus, prostate and placenta and Col1a1 is highly expressed in the uterus. Further evidence for the link between Porimin and reproduction was not available in the literature and therefore results from this particular gene are taken with caution until this protein is further characterized. Col1a1 plays an important role during spermatogenesis where it mediates the detachment and migration of germ cells, thus adding further support for its role in reproduction [12].

We have analyzed these data with an approach sensitive to all the systematic biases and limitations of methods given above. A number of genes in our dataset have been analyzed previously but have not taken these limitations and considerations into account. We have expanded these datasets to include a greater number of taxa, we have analyzed all of these genes for evidence of systematic biases and we have used improved models of codon evolution. In this paper we have included models that allow for rate variation across the sequence and across the phylogeny.

## Results and Discussion

**Figure 1:**
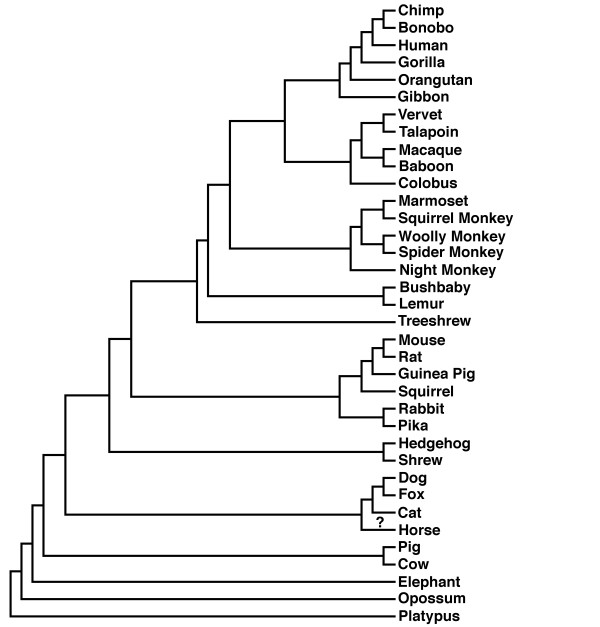
**Canonical mammalian species phylogeny**. Shown here is a representation of the agreed relationships amongst the *mammalia *for the species used in this analysis. The "?" on the lineage leading to horse indicates controversy over the position of this lineage on the phylogeny.

**Figure 2:**
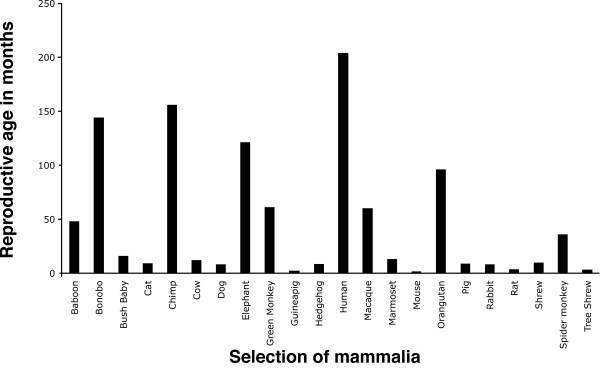
**Selection of mammalia used in the analysis and the time to reproductive age in months**. Species are shown on the X-axis in alphabetical order. On the Y-axis is the number of months it takes for each species to reach reproductive age.

We performed phylogenetic analyses on all 11 datasets. The resultant gene trees were found to conflict with the canonical phylogeny species ([13], as adapted in Figure 1. The only exception was the Catsper1 mammalian dataset. We postulate the following causes for this conflict: (1) amino acid and/or base composition bias, (2) lack of phylogenetic signal in the data, and finally (3), LBA caused by mixtures of long and short germ line generation times (see Figure 2 for a sample of species and their germ line generation times from our dataset). What follows is a summary of the results of the tests of data quality and bias we performed, see Table 1 for synopsis. We carried out these tests to determine in each case whether these conflicting phylogenies are accurate descriptions of history or whether the data are subject to these known issues listed 1-3 above. Subsequent statistical comparison of the gene trees and species phylogeny using the Shimodaira Hasegawa (SH) test [14] revealed that there is no statistical difference between the gene and species trees in each case, see Table 2 for results of SH tests. The only exceptions were Prkar2a and ZP3 where the presence of polytomies in the gene trees caused the preference of the unresolved nodes over the resolved nodes.

**Table 1:** Summary of the analysis of quality and bias present in the data

GENE	DATA QUALITY			PHYLOGENETIC ANALYSIS		
		
	LM Category	AA Comp Bias	Base Comp Bias	Substitution Model	Gene v Species Tree	LBA Artifact
**Adam2**	1	Pass	Pass	JTT+G	Unresolved	No
**Catsper1 Exon1**	1	Pass	Pass	JTT+I+G+F	Unresolved	No
**Catsper1 Mammals**	1	Pass	Pass	JTT+G+F	Unresolved	No
**Col1a1**	1	Pass	Pass	JTT+G	Unresolved	No
**Ph20**	1	Pass	Pass	JTT+G+F	Resolved	Yes
**Porimin**	1	Pass	Pass	JTT+G+F	Unresolved	No
**Prkar2a**	2	Pass	Pass	JTT+I+G	Unresolved	No
**Semg2**	1	Pass	Pass	JTT+G+F	Unresolved	No
**Sp56**	2	Pass	Pass	JTT+I+G	Unresolved	No
**Zp2**	1	Pass	Pass	JTT+G	Unresolved	No
**Zp3**	1	Pass	Pass	JTT+G+F	Unresolved	No

Genes with significant signal are given in the Likelihood mapping, or, L.M. Category column, see text for explanation of the category 1 and 2 in this column. Results of the amino acid composition and nucleotide base composition bias tests, are shown in the A.A. Comp Bias and Base Comp Bias columns respectively. The phylogenetic trees for each gene are drawn using the substitution model described where G = gamma distributed rates across sites, I = invariable sites, F = frequency of amino acids, JTT = Jones Taylor Thornton model. In the case of LBA analysis, No = no evidence of LBA in the gene analysed, Yes = evidence of LBA in the gene analysed.

**Table 2:** Summary of SH tests for complete gene datasets

Gene	SH - gene	SH - ideal	Best-fit Tree
Adam2	1.0000	0.1200	NS
Catsper1 Exon1	1.0000	0.1460	NS
Catsper1 mammals	0.5020	1.0000	NS
Col1a1	1.0000	0.2650	NS
Ph20	1.0000	0.3220	NS
Porimin	0.4040	1.0000	NS
Prkar2a	1.0000	0.0490	gene
Semg2	1.0000	0.1010	NS
Sp56	1.0000	0.2380	NS
Zp2	0.1620	1.0000	NS
Zp3	1.0000	0.0050	gene

For each gene, the likelihood of estimated Bayesian phylogeny (gene) and corresponding ideal species tree (ideal) to fit the dataset were determined with the SH test at a 5% significance level. Values equal to 1.0000 represent the tree with the lowest log likelihood, values less than 0.05 refer to those cases where there is a significant difference between the two topologies, and the gene tree is a significantly better fit to the data. NS = No Statistical significance between gene and species tree, in these cases the species tree was used.

### 1. Tests of Data Quality and Bias

#### (i) Test for amino acid and base composition biases

We tested all multiple sequence alignments (MSAs) for evidence of significant levels of amino acid composition bias and base composition bias in each lineage using the TreePuzzle software [15]. We found that all alignments passed the significance test with p-values < 0.05, see Table 1 for summary. For full set of amino acid and base composition bias test results, see Additional Files 1 and 2 respectively. In summary the discordance between each of the gene trees and the canonical species phylogeny is not a result of amino acid or base composition biases providing evidence of false relationships.

#### (ii) Test for phylogenetic signal

**Figure 3:**
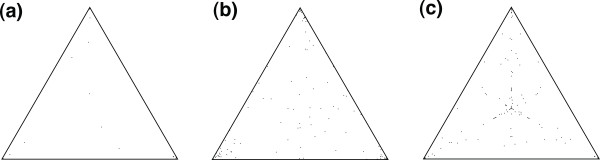
**Example of likelihood mapping categories**. **(a), category 1 **genes with strong phylogenetic signal the example given here is the ZP2 gene, **(b) category 2 **genes with intermediate levels of phylogenetic signal, the example given here is the Prkar2a gene, and **(c)**, **category 3 **genes with low/no phylogenetic signal, the example given here is the CD9 gene.

We performed the likelihood mapping procedure implemented in the TreePuzzle software [15,16] to determine the level of phylogenetic signal/conflict present in each alignment, for more detail see the *Methods *section. Our initial dataset consisted of 27 genes, we used this filtering step to reduce our dataset to contain only those genes with phylogenetic signal. We categorized the results from the likelihood mapping analysis into 3 main categories of signal: category 1 had strong phylogenetic signal (see Figure 3a), category 2 had medium level of phylogenetic signal (see Figure 3b) and category 3 had low/no levels of phylogenetic signal (see Figure 3c). The results of the test for phylogenetic signal are summarized in Table 1 and in total 9 out of the 27 genes had strong phylogenetic signal (category 1), with an additional 2 genes with moderate levels of phylogenetic signal (category 2). The complete set of results for the likelihood mapping process is given in Additional File 3. The remaining 17 genes failed the test (category 3). The category 3 genes (with low or no levels of phylogenetic signal) were subsequently removed from the analysis, only 10 genes were retained for further analysis.

#### (iii) Long Branch Attraction (LBA) analysis

We assessed the data for evidence of LBA which would manifest itself in the data by drawing species with a greater number of mutations in the gene of interest together erroneously on the phylogenetic tree. The method applied uses the MSA and the corresponding phylogeny to categorise rates amongst sites, using an approach we have previsouly published for mammalian data [17], as described in detail the *Methods *section. In this method of site-stripping we apply the phylogenetic tree (estimated *ab initio *in this software) and the MSA to classify all sites in the alignment into one of eight categories of mutation rate. These are arbitrary categories from 1-8; with 1 being the most highly conserved sites and 8 being the most highly variable. Essentially, these estimates allow us to select only the most conserved sites for phylogeny reconstruction. Sites are sequentially stripped from the alignments based on their rate of evolution and phylogenies are created based on slower evolving sites. These site-stripped phylogenies are then compared to the species tree. Using two independent methods of comparison we determined whether the resultant stripped trees had topologies significantly similar to the species phylogeny. The "root mean squared deviation", or RMSD, method is restricted to binary trees [18], see Additional File 4 for full set of results. Therefore we also employed the SH method of comparing phylogenies [14], see Additional File 5 for full set of results. For a full description of the RMSD statistic used here [18], see the corresponding section in the *Methods*. Using this approach we could identify the signature of LBA in the Ph20 dataset alone, see Table 1 for summary.

### 2. Analysis of selective pressures using codon models of evolution

Following analysis of the phylogenies of these reproductive genes, we determined the selective forces at work on these 10 genes (11 datasets). Only those genes passing the data quality tests were analyzed here (*i.e*. 10 genes), see Table 1. In the case of Catsper1, we have analyzed the gene at two different evolutionary distances because it contains high levels of insertion and deletion events. The two datasets for Catsper1 are: exon 1 from the primates only, and, the entire gene from only distant mammalian groups. Hence the number of datasets is 11, and the number of genes tested is 10. The alignments in all cases reached significant levels following randomization tests (z-scores > 1000 in all cases, a z-score of greater than 5 is typically taken as significant).

**Figure 4:**
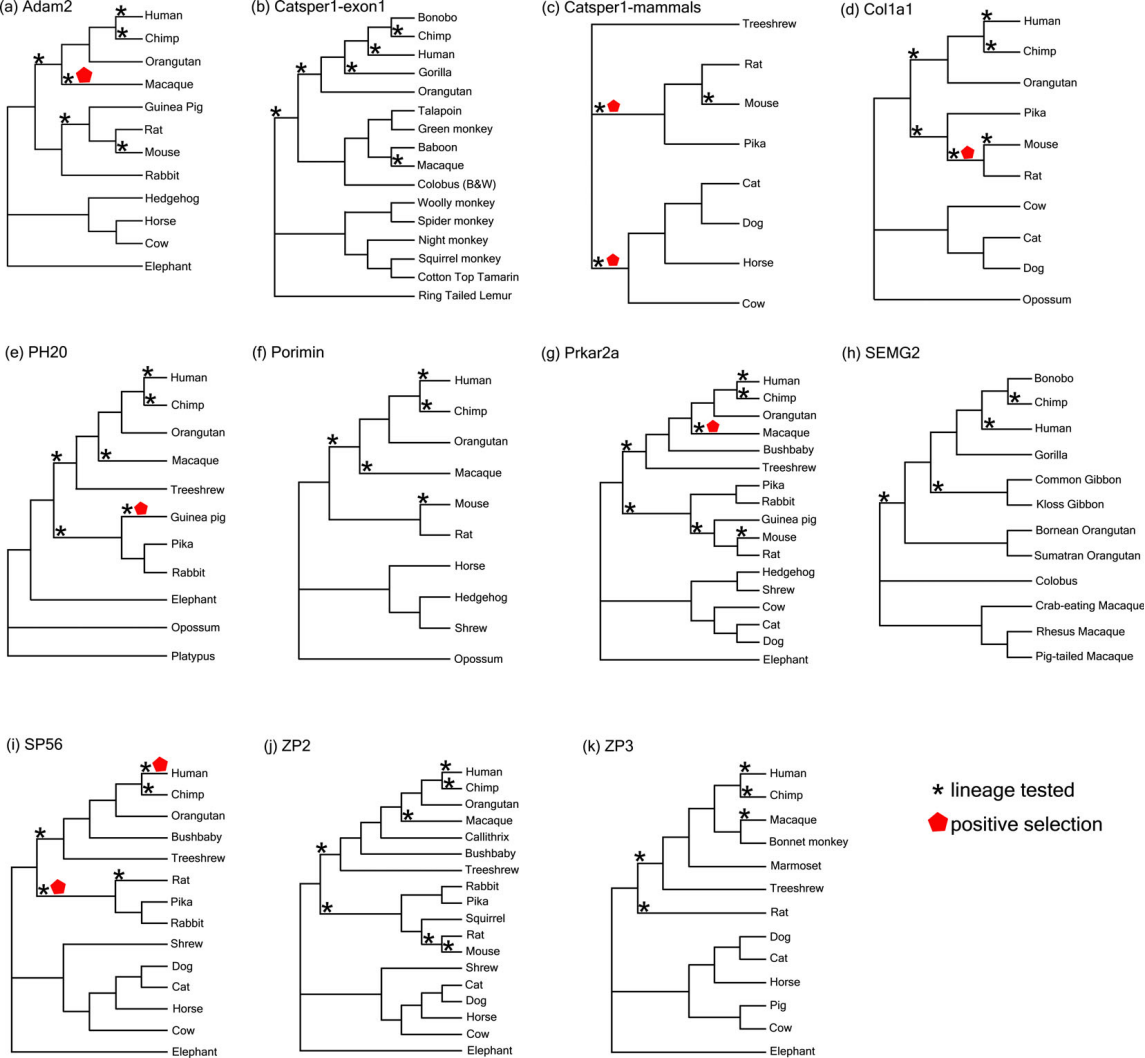
**Results of lineage specific positive selection analysis on 11 datasets**. The phylogeny used for each gene is a reduced version of the species phylogeny. The lineages labeled as foreground in the analysis are denoted in the diagram with asterix symbols. These are the results following LRT analysis. Those lineages where positive selection was determined are represented by red pentagons.

In those cases where the genes had already been analyzed in previous studies, we expand upon the data in these studies and use more sophisticated models of evolution. ML methods are sensitive to sample size with a minimum of 6 taxa recommended from simulation studies [10]. For a summary of the site-specific and lineage-specific results, see Table 3 and Table 4 respectively. For a summary of all likelihood ratio tests (LRTs) performed in the analyses of these genes see Table A9. In general the lineages tested in the lineage specific analysis for each gene were as follows: modern human; the primate ancestor; modern mouse, and the rodent ancestor, these are indicated in Figure 4(a-k). For certain datasets the species tested varied depending on those species for which high quality sequence data existed for that gene, these are discussed on a gene-by-gene basis below. In summary, for each of the 11 datasets tested, positive selection was detected. In the site-specific test between 7 and 94 sites per gene were identified as positively selected. In the lineage-specific analyses there were up to 2 lineages per gene identified as having evidence of positive selection. Below is a brief description of the results on a gene-by-gene basis, the complete set of all parameters, likelihood values and LRTs are given in Additional File 6.

**Table 3:** Summary of the results of the site-specific analysis: in each case the most significant model was M8

Gene	n	Parameter estimates	# Positively selected Sites
Adam2	12	p_0 _= 0.92632 p = 0.37637 q = 0.60688 p_1 _= 0.07368 ω = 3.94326	45>0.50 15>0.95 5>0.99

Catsper1_Exon1 (primates only)	16	p_0 _= 0.82736 p = 0.13661 q = 0.03850 p_1 _= 0.17264 ω = 3.13071	95>0.50 7>0.95 1>0.99

Catsper1_Mammals (non-primate mammals only)	8	p_0 _= 0.83315 p = 0.34233 q = 0.51278 p_1 _= 0.16685 ω = 3.26879	124>0.50 30>0.95 8>0.99

Col1a1	10	p0 = 0.98023 p = 0.04796 q = 0.32286 p1 = 0.01977 ω = 4.09285	66>0.50 21>0.95 8>0.99

Ph20	11	p_0 _= 0.87658 p = 0.56141 q = 0.83349 p_1 _= 0.12342 ω = 2.20500	39>0.50 3>0.95 0>0.99

Porimin	10	p_0 _= 0.85067 p = 0.41864 q = 0.32952 p_1 _= 0.14933 ω = 12.21841	30>0.50 13>0.95 5>0.99

Prkar2a	17	p_0 _= 0.95102 p = 0.16339 q = 0.98823 p_1 _= 0.04898 ω = 2.60992	19>0.50 4>0.95 0>0.99

Semg2	12	p_0 _= 0.97236 p = 0.01163 q = 0.00500 p_1 _= 0.02764 ω = 12.26405	41>0.50 5>0.95 2>0.99

Sp56	14	p_0 _= 0.98807 p = 0.16114 q = 1.12262 p_1 _= 0.01193 ω = 3.81710	8>0.50 2>0.95 2>0.99

Zp2	18	p_0 _= 0.87339 p = 0.63945 q = 0.75356 p_1 _= 0.12661 ω = 2.04655	52>0.50 9>0.95 6>0.99

Zp3	13	p_0 _= 0.91489 p = 0.30029 q = 0.77328 p_1 _= 0.08511 ω = 1.92305	48>0.50 0>0.95 0>0.99

Following LRT analysis M8 was chosen in each case as the most significant model. n refers to the number of taxa in each dataset. The proportion of sites (p), evolving under each corresponding selective pressures (ω) are shown. For example, p_0 _refers to the proportion of the protein evolving under the selective pressure value given by ω_0_. The parameters p and q describe the beta distribution. The final column gives the number of sites with posterior probability (PP) of 0.50, 0.95 and 0.99 that belong in the positively selected category or sites. The number before the ">" refers to the number of sites with a specific PP value.

**Table 4:** Summary of lineage-specific positive selection detected.

Species tested as Foreground	Significant LRT	Parameter estimates		
		P	Fwd *ω*	Bck *ω*
**Adam2**				
Macaque	ModelA v M1	9.57%	1.71	0.10/1
**Catsper1 Mammals**				
Ferungulata	ModelA v M1	4.46%	998.99	0.09/1
Rodents	ModelA v M1	5.45%	999.00	0.084/1
	ModelB v m3Discrtk2	4.47%	999.00	0.12/1.38
**Col1a1**				
Rodents	ModelA v M1	2.17%	72.73	0.013/1
	ModelB v m3Discrtk2	1.93%	72.77	0.02/1.35
**PH-20**				
Guinea Pig	ModelA v M1	6.3%	11.48	0.13/1
	ModelB v m3Discrtk2	6.14%	12.57	0.14/1.10
**Prkar2a**				
Macaque	ModelA v M1	2.37%	999.00	0.04/1
	ModelB v m3Discrtk2	2.53	999.00	0.04/1.22
**Sp56**				
Human	ModelB v m3Discrtk2	100%	62.40015	0.02/0.55
Glires	ModelB v m3Discrtk2	2.56%	1.03	0.02/0.55

Summary table of significant results for lineages specific analyses following LRT analyses. Lineages tested as foreground (Fwd) are shown in the first column. Only those lineages with significant LRT values for Model B or Model A and ω >1 are shown here. Parameter estimates are given for the LRT values highlighted in bold. P is the proportion of sites under selection the corresponding selective pressure as measured by ω. Fwd ω and Bck ω scores for the foreground species and background species respectively are given in the final column.

#### Col1a1

Possibly the most intriguing result from our entire analysis is that from the Col1a1 protein. According to the microarray study employed here [11], Col1a1 is highly expressed in the uterus tissue. It is also found in most structural tissues including cartilage, bone, tendon, skin and part of the eye (sclera). It is a member of the group 1 collagen proteins involved in the development of the uterine fibroids [19]. There are two propeptide regions to the Col1a1 gene, denoted N- and C-terminal propeptides. According to studies on Col1a1 function, a role has been established for Col1a1 in spermatogenesis [12].

**Figure 5:**
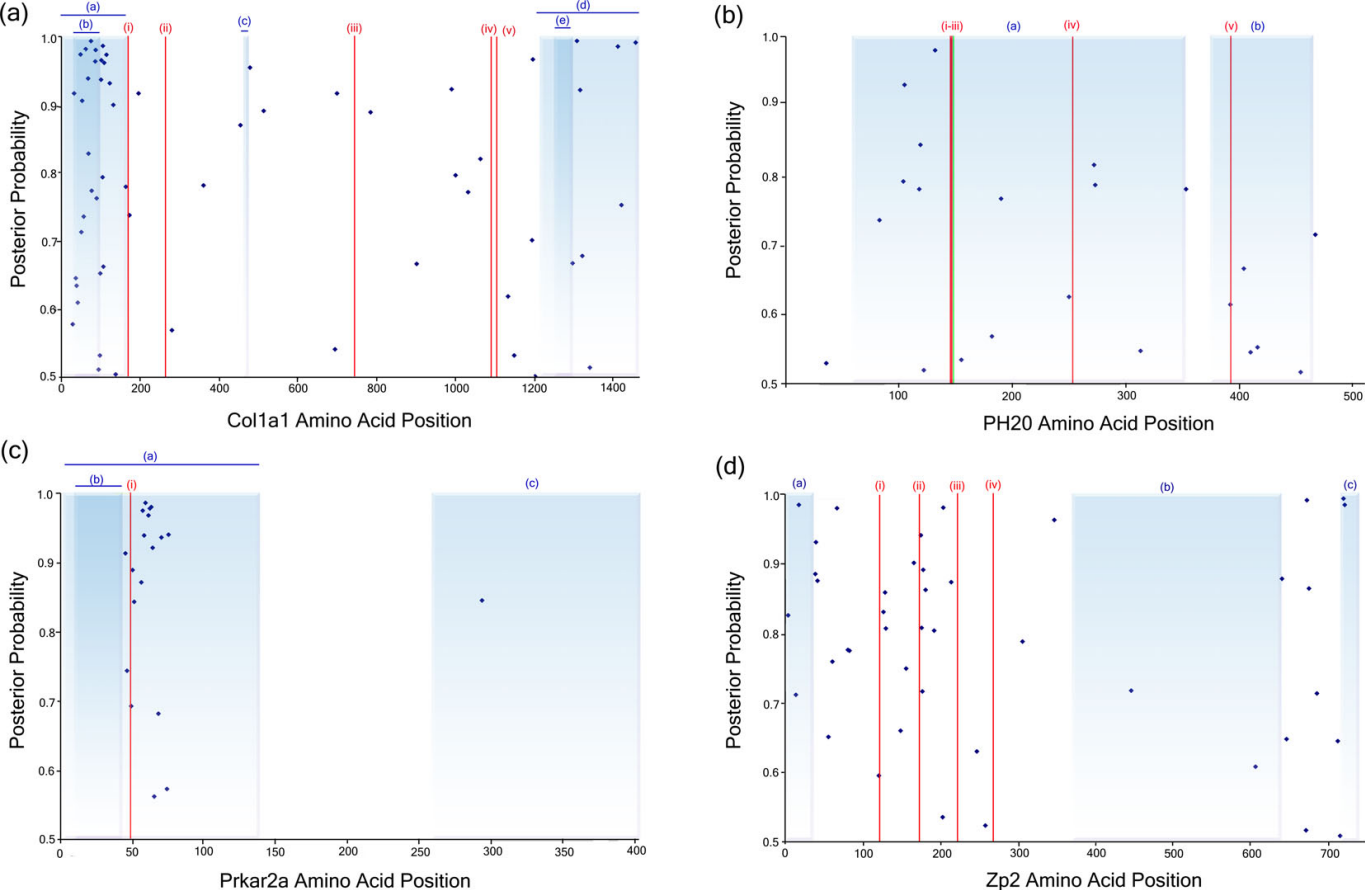
**Results of positive selection analysis for 4 genes**. Each of the four graphs represents the CDS of a gene from position 1 to the stop codon (X-axis). The Y-axis is the posterior probability of each of the sites belonging to the positively selected category. The dark blue data points are sites estimated to be under positive selection. Alternative pale blue and white regions depict alternative domains in the protein, this data is taken from Swiss-Prot. The vertical red bars in each case represent functionally important sites, these are specific to each gene as follows: **(a) Col1a1**, (i) Cleavage site by procollagen N-endopeptidase, (ii) O-linked Gylcosylation site, (iii) Cell attachment site, (iv) Cell Attachment site, and (v) O-linked Glycosylation site. **(b) PH20**, (i) active site, proton donor, (ii) and (iii) are positions when mutated result in loss of activity, and (iv) N-linked Glycosylation site, **(c) Prkar2a**, (i) and is a Phosphothreoinine modified residue, **(d) ZP2**, (i), (iii) and (iv) are N-linked Glycosylation sites, and (ii) is the cleavage site.

Our site-specific analysis shows 66 sites evolving with an ω value of 4.09, see Table 3. In summary 35/66 of our positively selected sites fall in the N-terminal propeptide region (23-161) and 9/66 positively selected sites fall in the C-terminal propeptide region (1219-1464), this can be seen clearly in Figure 5a. Position 162 in Col1a1 is cleaved and modified by an endopeptidase, position 162 is also modified by pyrrolidone carboxcylic acid (Swiss-Prot PO2452). A positively selected site at position 163 is neighboring this multifunctional site, suggesting that there has been an evolutionary effort to improve cleavage and/or modification in this protein.

Variations in Col1a1 are linked with Osteogenesis Imperfecta (OI), an autosomal dominant disease, resulting in an inability to make the correct collagen protein. There are a spectrum of OI conditions, the most severe is OI type 2 (OI-II) leading to death in the perinatal period. A recent extensive study of the Single Nucleotide Polymorphisms (SNPs) associated with OI has revealed a number of substitutions of glycine residues within the triple helical domains of the Col1a1 protein [20]. The total number of disease implicated sites in the Swiss-Prot entry P02452 for Col1a1 is 99: 4 of these are OI non-specific, 4 are OI-I, 59 are OI_II, 14 are OI-IV and 15 are SNPs (2 are associated with another disease). One third of the mutations that result in substitutions for glycine in Col1a1 are lethal whereas those between the start codon and position 200 are non-lethal. Only 1 of the sites we have identified as positively selected is in the non-lethal domain from position 1-200, this is site 195. This positively selected site is neighboring the SNP position 197 that causes a mild OI phenotype. In Table 5 we show a list of 11 positively selected sites that fall in close proximity to sites associated with disease and are located between 280 and 1456, spanning the important triple helix region. These positions are all within 1 to 5 amino acid residues of known disease variants, 8 of these disease variants are the severe/lethal OI-II disease form. Two exclusively lethal regions, helix positions 691-823 and 910-964 aligned with major binding regions [20] and we find a positively selected site in this region. Following a randomization test for the positively selected sites and disease implicated sites (as denoted by Swiss-Prot entry PO2452), we have found that the pattern we observe, i.e. finding positively selected sites in close proximity to disease implicated sites is significant in 3 out of the 11 cases examined here (at P < 0.05).

**Table 5:** Summary of the positively selected sites in the col1a1 gene, their clinical relevance, and, the probability of being located within distance "d" from the nearest disease-implicated site.

Positively selected sites	Posterior Probability	Human Variant: SNP position	Distance (*d*)	Probability of being *d* from nearest disease-implicated site	Genetic code distances between observed character states	Clinical Association	
195	0.926	197	2	0.04	A-N = 2	G → C	mild phenotype
280	0.588	275	5	0.26	A-S = 1; S-T = 1; T-A = 1	G → D	OI-II
478	0.959	476	2	0.128	A-S = 1; S-T = 1; T-A = 1	G → R	OI-II
784	0.968	776	8	0.396	A-S = 1; S-T = 1; T-A = 1	G → S	OI-II
1032	0.535	1025	7	0.364	A-P = 1	G → R	OI-II
1063	0.826	1061	2	0.128	N-S = 1	G → D	OI-II
		1061	2	0.032	N-S = 1	G → S	OI-IV
1149	0.623	1151	2	0.032	A-S = 1	G → S	OI-III
		1151	2	0.128	A-S = 1	G → V	OI-II
1194	0.675	1195	1	0.076	A-G = 1; G-S = 1; S-A = 1	G → C	OI-II mild form
1196	0.972				A-F = 2; F-Y = 1; Y-A = 2		
1316	0.928	1312	4	0.24	K-N = 1; N-P = 2; P-K = 2	W → C	OI-II
1456	0.997	1460	4	0.1	C-F = 1; C-L = 2; C-M = 2; F-L = 1; F-M = 2; L-M = 1	P → H	dbSNP: rs17853657

The sites under positive selection in the col1a1 protein and their associated posterior probabilities (PP) are shown. The third column shows variant positions (SNPs) as determined using Swiss-Prot human (PO2452) sequence. The fourth and fifth columns show the residue distance "d" of the positively selected site from its nearest genetic variant, and the probability of being located "d" residues from any disease implicated site by random chance alone. The sixth column uses single-letter amino acid symbols to show the genetic code distances between all observed character states at each positively selected site. "Clinical Association" show the replacement substitution at the human variant position and its clinical association with that human variant. OI = Osteolysis imperfecta, OI-I to -IV. The final entry for dbSNP is database entry number rs17853657 and as yet has not been associated with OI although it is in the same domain as the other disease-causing SNPs.

Lineage-specific analysis shows evidence for positive selection in this protein in the rodent ancestor. In total, 2.2% of the sites in the rodent ancestor have ω = 72.73, while the rest of the species are evolving under purifying selection, ω = 0.013. For a summary of site and lineage specific results for Col1a1, see Table 3 and 4. For complete set of results see Additional File 6(d).

#### Prkar2a (interacts with SEMG2)

Prkar2a is a cAMP dependent protein kinase that is attached to the sperm flagella via regulatory subunit (RII) [21]. Protein tyrosine phosphorylation has been linked with successful fertilization due to hyper-activated sperm motility [22]. This increase in phosphorylation is part of a cAMP dependent pathway that activates protein kinase A [22].

The PRKA families were previously tested for positive selection using 3 to 4 taxa and site-specific model M8 with no significant results for positive selection reported. With our 17 taxa dataset, we were able to detect that 4.7% of sites were evolving at a rate of ω = 2.60, see Table 3 for summary of details.

Positively selected sites detected in the site-specific analysis of Prkar2a were compared to the human Swiss-Prot sequence (P13861). In total 18 sites were predicted to be positively selected, 17 of these sites occur in the region of the protein associated with dimerization and phosphorylation (2-138), see Figure 5(c). In the Swiss-Prot entry there are a number of residues listed as being modified by phosphoserine. These are positions 58, 78, 80, 99 and phosphothreonine at position 54. The sites estimated to be positively selected from our analysis are: 58, 59, 61, 62, 63, 64, 65, 68, 70, 74, 75, these sites are at or in close proximity to these modified residues.

The regulatory subunit alpha 2 of Prkar2a has been shown *in vitro *to interact with Semg2. The phosphorylation of Semg2 may lead to its activation into forming a gel matrix in the female reproductive tract. From our analysis it is shown that while Semg2 has positively selected sites dispersed throughout its sequence, whereas the positively selected sites for Prkar2a are localized to the N-terminus region, and the remainder of the gene is under strong purifying selection. Literature has so far not specified an exact phosphorylation site for Semg2, which prevents us from commenting further on its interactions with Prkar2a.

Lineage-specific analysis shows that Prkar2a in the macaque has undergone a greater selective pressure to change when compared with other *mammalia *in the dataset, with 2.53% of sites evolving at ω = 1.22, see Table 4 for summary of results. For complete set of results for Prkar2a, see Additional File 6(g).

#### Ph20 (interacts with ZP2 and ZP3)

Ph20 is expressed in the testis and found in the acrosome of the sperm. It is also codes for a receptor that is involved in the sperm to zona pellucida (ZP) adhesion [23].

Previous analysis conducted on this protein involved 6 taxa [24]. Here we have increased the number of taxa to 11. We have omitted the carnivores from our analysis of Ph20 as the sequences were spurious. We found evidence for LBA in the Ph20 dataset. By removing fast evolving sites a fully resolved gene phylogeny is obtained. This gene tree now is in agreement with the ideal species phylogeny ([13].

Lineage-specific analysis shows that guinea pig is under positive selection, with 6.1% of sites with ω = 12.57 while all other species in the background are evolving at ω = 0.14 or neutrally, see Table 4. The 39 positively selected sites were then compared to the human Swiss-Prot sequence (P38567), see Figure 5(b) for results. Catalytically important resides 146, 148, 211 284 and 287 when mutated result in a reduction in, or loss of, activity [25]. It has been shown experimentally that mutations in the region of this active site significantly reduce or completely block the function of this protein [25]. Our results show that 3 of the positively selected sites, 155, 272, 273, are in close proximity to these regions. Another 5 positively selected sites: 83, 155, 252, 353 and 391 are close to glycosylation sites, see Figure 5(b). These sites when modified are known to change the structure and function of the Ph20 protein. For complete set of results for Ph20 see Additional File 6(e). These results are of significance as the Ph20 protein changes position in the sperm during the different stages of the fertilization process. In guinea pig Ph20 protein is known to migrate from the post acrosomal membrane to the inner acrosomal membrane [26]. Thus finding these positively selected sites in close proximity to these glycosylation sites in guinea pig suggests that these sites have been selected to modify the Ph20 structure more effectively thus increasing the chance of capacitation.

#### SP56 (interacts with ZP2 and ZP3)

The binding of sperm to the zona pellucida (ZP) is crucial for gamete formation to take place. The exact mechanisms of this process are still to be uncovered therefore any predictions on important residues will greatly improve knowledge by directing mutational studies. SP56 has been shown through photoaffinity cross-linking experiments to have a specific binding affinity for ZP3 [27]. Therefore it is believed to play an important role in the binding of sperm to the ZP matrix. Experiments have shown that during capacitation SP56 is released from the acrosomal matrix and becomes situated in the sperm head membrane, enabling it to act as a ZP3 binding protein [28].

Here we have found 8 positions in the SP56 protein that are under positive selection (ω = 3.82) following site-specific analysis. These sites were compared to the human SP56 entry in Swiss-Prot (Q13228) to determine possible links to function. One of these 8 positively selected sites is position 122, regarded as a SNP number (rs35396382) in dbSNP database [29]. Although further experimental work needs to be conducted to decipher the clinical association of this position, it is extremely interesting that our most significant positively selected site also displays variation in the population, especially given the overall high level of conservation in this gene. For summary of results see Tables 3 and 4, and for full set of results for this gene see Additional File 6(i).

#### ZP2

Zona pellucida (ZP) proteins form the complex glycoprotein coat that surrounds the oocyte [30]. These ZP proteins have been shown to be under strong pressure to change, and results have been published on both site and lineage analyses [31]. Here we have expanded the analysis of ZP2 to include 18 taxa (maximum previously tested = 8 [31]). We have also applied more complex models of evolution and have sampled deeper branches on the phylogeny including a representative of the *Afrotheria *- elephant.

In this case, the results of our larger dataset and more complex models show that the values of ω determined here vary slightly when compared to previous analyses [31]. This previous test showed 4.7% of sites to have ω = 2.5, increasing the size of the dataset in this study results in 52 sites in ZP2 that have an ω value of 2.05. See Additional File 6(j) for complete results.

Positively selected sites were compared to the human Swiss-Prot entry for ZP2 (Q05996) to identify possible function for these sites, see Figure 5(d). ZP2 contains 7 carbohydrate chains situated between sites 87-462, these are important for the sperm to bind to the ZP of the egg coat [32]. Of the 46 sites identified to be under positive selection, 23 fall between positions 66-257, this region contains 5 of the binding domains of the carbohydrate chains. The clustering can be seen more clearly in Figure 5(d). Another cluster of positively selected sites (10 sites in total) occurs in the propeptide region (641-745). It has been suggested that upon the cleavage of the propeptide region, the mature ZP2 protein plays a role in the prevention of polyspermy [33].

#### ZP3

Analysis of site-specific evolution in ZP3 identified 48 positively selected sites. Of specific interest are positively selected positions 329, 330, 332, 336, 338, 339, as these sites were in close proximity to identified sperm binding sites (329-334) [34], see Table 3. The furin cleavage site is identified at position (350-353), and the propeptide domain at position (351-424). When cleavage takes place the ZP3 undergoes a conformational change that inhibits any further sperm binding to the coat thus preventing polyspermy [35]. Of the 48 positively selected sites identified, 10 fall within the propeptide domain, with an additional 12 occurring close to the vicinity of the furin cleavage and sperm binding sites, thus suggesting that there is a pressure to improve binding and prevent polyspermy. For complete set of results for ZP3, see Additional File 6(k).

#### Adam2 (Fertilin β)

Adam2 is a cell adhesion molecule that plays a fundamental role in the final binding of sperm to the oocyte membrane [36]. Indirect interactions have been shown with female proteins CD9 [37]. (We have not continued further analysis on CD9, as it failed the likelihood mapping test).

Previous results have been published reporting positive selection using site-specific analysis on 6 taxa [24]. Here we have included 12 taxa for Adam2 and we have investigated the possible functional implications of positively selected sites found. In the site-specific analysis we find 7.3% of sites with ω = 3.94, this corresponds to 45 sites in total, see Table 3. Comparison of these positions to human Swiss-Prot Adam2 sequence (Q99965), we determine that 39/45 positively selected sites are situated in the C-terminus region. On closer investigation of these sites we find that 12/45 positively selected sites occur in the disintegrin domain (position 384-473). The disintegrin domain has been shown to be involved in the binding of Adam2 to the oocyte [38]. A cysteine-rich domain occurs between (477-606), 16/45 positively selected sites fall in this region. It has been suggested for Adam12, (another member of the Adam family of proteins), that the cysteine-rich domain plays a role in mediating the cellular interactions via syndecans and integrin [39], a similar role for this domain in Adam2 can be postulated. Overall the results for Adam2 suggest a selective pressure for increased binding of Adam2 to the oocyte regardless of species of origin. For a complete set of results and LRTs for Adam2, see Additional File 6(a).

#### Catsper1

Catsper1 is involved in regulating the calcium cation channel in sperm flagella, the result of which is movement of sperm [40]. Previous studies on Catsper1 exon 1 have been performed [41]. We intended to expand our analysis to span all exons and expand the data set to include a variety of *mammalia*. However, the exon 1 of non-primate *mammalia *is so highly variable that an accurate alignment cannot be constructed. The remaining exons were highly conserved across all species. We therefore split our catsper1 dataset into two sections each of which produced a good quality alignment for analysis, (1) exon1 of catsper1 for the primates, and (2) entire catsper1 gene for non-primate *mammalia*.

##### (a) Catsper1 Exon 1 primates

Site-specific analysis of this protein identified 17% of the protein under positive selection with ω = 3.13. Previous analysis of this exon showed positive selection on indel substitutions in this gene [41]. The positively selected sites are situated throughout exon1, little is known about the functional significance of these sites. However, it is known that exon 1 has a significant role to play in altering the rate of calcium ion channel inactivation. Different lengths in the N-terminus result in different rates of channel inactivation, where a long terminus results in a longer time to activation than the shorter terminus. This is described most effectively by the ball and chain mechanism described in [41]. See Additional File 6(b) for complete results. These results show the importance of this protein, and specifically the first exon, for reproductive success.

##### (b) Catsper1 entire gene non-primate mammals

Our site-specific analysis identified 16.7% of the sites under positive selection with an ω = 3.27, see Table 3. These sites all cluster in exon 1. While the rodent ancestor appears to be under positive selection with 4.47% of its sites evolving at ω = 999, see Additional File 6(c) for complete set of results. A previous study of 9 rodent species, including *Mus musculus *individuals from 4 different populations, has shown that within the rodent order there has been a continued pressure to evolve, with positive selection for indel substitutions in exon1 of the Catsper1 gene [43].

#### Semg2

A member of the family of semenogelin genes, Semg2 is involved in the formation of a postcopulatory plug [44]. Previously, positive selection has been reported for both site-specific and lineage-specific analysis for Semg2 [9,45]. We have expanded the data set from previous analyses to incorporate more species.

In our site-specific analysis, we found that 2.7% of our sites had an ω value of 12.26, see Table 3.

We have performed a novel functional analysis of these positively selected sites by comparing them to the human Semg2 sequence (Q02383) in the Swiss-Prot database. This is a step not previously taken by other studies of Semg2. A striking pattern emerged - all known domains of this protein have several positively selected sites. There is a probable glycosylation site at position 272, which is located close to a large stretch of positively selected sites (positions 262 to 289). It is so far unknown how significant this glycosylation site is in Semg2 and whether it plays a role in modifying the protein to form a copulatory plug. However, the results indicate that this protein, and in particular the region around the glycosylation site, has been under significant pressure to change.

A complete set of results for Semg2 is given in Additional File 6(h). The lineage-specific results are not described here in detail as lineage analyses have been carried out previously on the primate Semg2 gene [9,45]. It has been shown recently that the rate of evolution for this protein varies depending on the level of sperm competition [9]. Our results are in agreement with this finding, thus further verifying our approach.

#### Porimin

Two isoforms of this protein have been identified; we have focused on isoform 1 in the *mammalia*, as isoform 2 contains an additional human specific region between residues 34-52. To date the exact mechanisms of this transmembrane receptor are unknown. This protein is not well characterized biochemically and its function cannot be verified as reproduction related, therefore we only discuss the results briefly below.

On site-specific analysis of this protein we determined that 30 of the sites are under positive selection (ω = 12.22), see Table 3. From analysis of the sites on the Swiss-Prot entry for human Porimin (Q8N131), we could determine that two positively selected sites (146 and 147), were found in a highly conserved region and fall in close proximity to the N-linked glycosylation site. For complete set of results for Porimin, see Additional File 6(f).

## Conclusion

Testing for phylogenetic signal and biases, such as amino acid composition bias and LBA, indicated that there was adequate phylogenetic signal for 10 of the genes and in general no evidence of systematic biases. On testing for LBA, Ph20 was the only protein in this dataset that displayed the typical signature of this bias with gene and species tree agreement being maximized with the removal of the fastest evolving categories. This would suggest that while germ line generation times vary greatly in the dataset, the effect of the resultant LBA does not impact on the sequence data to any great extent (1/11datasets).

Selective pressures for the reproductive proteins studied here are heterogeneous. All proteins exhibited regions of strong conservation proving the importance of maintaining structural stability and overall function in these proteins. All but 1 protein (Adam2) exhibited evidence of positive selection in specific lineages, and all proteins without exception exhibited positive selection in regions of catalytic/functional importance. For SP56 and Col1a1 the site-specific results are entirely novel. The lineage-specific results described here for Prkar2a and Catsper1 exon 1 in primates, are also novel. We have shown that, in the case of Catsper1, there is a fundamental protein functional shift between new world monkeys and old world monkeys. The Dn/Ds measurement applied here assumes that neutral substitution rate is akin to Ds, therefore no selection on silent sites. There have been many publications of late to the contrary therefore we are mindful of examining the rate of silent substitution in all our analyses [46,47].

For the reproductive genes in our dataset, we show that lineages evolve at unique rates and at functionally crucial sites, specifically those involved in phosphorylation. We have also shown that a number of these proteins (Col1a1 and Catsper1) show positive selection for example in the ancestral rodent lineage and evidence of purifying selection in the subsequent divergent species.

Overall our analyses of these reproductive proteins show how important it is to carefully examine data for systematic biases prior to testing for lineage and/or site specific positive selection. We have also demonstrated the importance of including large numbers of taxa/lineages in these analyses. This finding was highlighted in our analysis of Prkar2a where previous analysis of this protein had included only 4 taxa and therefore reported a negative result. We do not observe any large-scale effect of germ line generation time in our dataset, with only 1 protein (Ph20) with evidence of long branch attraction. The results of Col1a1 indicate that the positively selected sites may have been of such importance for this protein that neighboring mutated sites may have been maintained in the population despite their propensity for causing disease. The location of positively selected sites determined using this approach and in regions of functional importance in the proteins in this dataset, provides us with further evidence of the link between functional shift and positive selection.

## Methods

The data analyzed in this study consist of homologous reproductive genes from a variety of mammalian genomes. Genes were identified as being reproduction related from literature searches, analysis of protein interaction networks (iHOP) [48] and expression (microarray) data [11]. The microarray expression data used is from normal human tissues. We have also included a more in-depth analysis of previously identified cases of positive selection in reproductive proteins. A list of all data used in this study are available in Additional File 7, the total number of genes analyzed was 10. Homologs of all 10 reproduction related genes were identified in mammalian genomes that span the entire phylogeny of mammals, see Figure 1. For each of the reproduction related genes, the alignment of homologs contained between 10 and 18 species, and the alignment length varied between 351 and 4374 base pairs.

### Sequence Data

Protein coding sequences for the reproductive proteins were retrieved by the combination of two methods; Ensembl and Blast searches. Orthologous coding sequences from all available completed mammalian genomes were retrieved from the Ensembl database [49]. These orthologs had been identified previously by performing a genome-wide reciprocal WUBlastp+SmithWaterman search of each gene across all completed genomes. To include those *mammalia *that were not present in Ensembl a BlastP search was conducted on all the human amino acid sequences from each gene against the Swiss-Prot database.

### Mammalian Species

**Primates:** Human *(Homo sapiens)*, Chimp (*Pan troglodytes)*, Bonobo *(Pan paniscus)*, Bornean Orangutan *(Pongo pygmaeus)*, Sumatran Orangutan (*Pongo abelii*), *Gorilla (Gorilla gorilla)*, Rhesus Macaque *(Macaca mulatta)*, Crab eating *Macaque *(*Macaca fascicularis*), Pigtailed Macaque (*Macaca nemestrina)*, Bonnet monkey *(Macaca radiata)*, Baboon (*Papio hamadryas*), Mantled Guereza *(Colobus guereza)*, Vervet Monkey (*Cercopithecus aethiops*), Angolan Talapoin (*Miopithecus talapoin*), Squirrel Monkey (*Saimiri sciureus*), Cotton top tamarin (*Saguinus oedipus*), Common Marmoset (*Callithrix jacchus)*, Marmoset/Callithrix *(Callithrix-jacchus)*, Spider Monkey *(Ateles geoffroyi*), Bushbaby *(Otolemur garnettii*), Common woolly monkey (*Lagothrix lagotricha*), Ringtailed lemurs (*Lemur catta)*, Kloss Gibbon *(Hylobates klossii)*, Common/Lar Gibbon *(Hylobates lar)*, Night/owl Monkey *(Aotus trivirgatus boliviensis)*. Scandentia: Treeshrew *(Tupaia belangeri)*. Rodents: Mouse *(Mus musculus*), Rat *(Rattus norvegicus)*, Guinea pig *(Cavia porcellus)*, Ground Squirrel/Squirrel *(Spermophilus tridecemlineatus)*. Lagomorpha: Rabbit (*Oryctolagus cuniculus*), Pika (*Ochotona princes*). Eulipotyphila: Hedgehog *(Erinaceus europaeus)*, Shrew (*Sorex araneus*). Carnivores: Cat *(Felis catus)*, Dog *(Canis familiaris)*. Artiodactyla: Cow *(Bos taurus*), Pig *(Sus scrofa)*. Perisodactyla: Horse *(Equus caballus)*. Proboscidea: Elephant *(Loxodonta africana)*. Monotremata: Platypus *(Ornithorhynchus anatinus)*. Didelphimorphia: Opossum *(Monodelphis domestica)*.

### Multiple Sequence Alignment (MSA)

All coding sequences were translated into their corresponding amino acid sequences using in-house translation software. Gene family alignments were generated at protein level using ClustalX 1.83.1 using default parameter settings [50]. The corresponding nucleotide gene family datasets were aligning based on their protein alignments using in-house software. Each gene family alignment was manually edited using Se-Al [51] to remove any ambiguous regions.

### Nucleotide composition bias, amino acid composition bias and likelihood mapping tests

TreePuzzle 5.2 [15] performs a chi-square test that compares the amino acid composition of each sequence to the frequency distribution assumed in the General Time Reversible (GTR) and Jones Taylor Thornton (JTT) models [52]. Ideally no species should fail this test, however, where two species fail and are thus drawn together on a tree, these sequences are excluded. Using the likelihood mapping method, each tree is disassembled into its constituent quartets and the support for each possible quartet is assessed. If the data contains phylogenetic signal then the likelihood of all three possible relationships for that quartet will be equally likely, these are represented by the three tips of the triangle, and the majority of the signal will be in these tip regions. Otherwise, the vertices and central region will be most heavily populated by supporting quartets.

### Phylogeny Reconstruction

Phylogenetic trees were constructed using MrBayes v3.2.1 [53] and the amino acid sequences. Amino acid sequences were used in order to vitiate the effects of base and codon compositional biases. The substitution model was selected following model testing using Modelgenerator version 85 [54]. The selected model was JTT, the GTR rate model was implemented and the first 20000 trees for each gene were discarded as "burnin". A majority rule consensus tree from the remaining trees sampled was constructed for each gene. The parameter settings for each gene phylogeny are summarized in Additional File 8.

### Site-stripping for significance

To test for long branch attraction (LBA) we applied the slow-fast approach of Brinkman and Phillipe [55]. We implemented the rate categorisation in a maximum likelihood framework in TreePuzzle 5.2 [15]. This software takes the alignment as input and generates *ab initio *phylogenetic trees. It then calculates the rate of mutation for each site in the alignment. The software specifies 8 arbitrary categories of site: each one of these categories contains some portion of the alignment. In this manuscript 8 is the most rapidly evolving (for example every lineage has a different character state for that character), and category 1 is the most slowly evolving (for example each lineage has the same/identical character state for that character). Sites are then progressively removed from the protein MSA according to their evolutionary rate, and at each stage a new phylogenetic tree is constructed based on this slightly reduced dataset. The difference between the new topology created on a reduced alignment and the original topology reconstructed based on the entire alignment are then compared in a statistical framework to determine which fits the data best (SH Test 2, see below) or which is most similar to the species phylogeny (RMSD Test 1, see below). At each stage we employ MrBayes [56] to perform the phylogenetic reconstruction using the aforementioned settings.

### Tests of the difference between two trees

#### Test 1: Nodal distance calculation

TOPD/FMTS v 3.3 [18] calculates the distance between the site-stripped trees and the 'ideal' tree. The 'ideal' tree used for each gene was a pruned version of the canonical species tree as seen in Figure 1. A distance matrix is derived by counting the number of nodes that separate each of the taxa in a tree. A distance matrix is calculated for each site-stripped tree as compared to the ideal species tree. The nodal distance score is obtained by calculating the RMSD of the matrices. If both trees are identical the RMSD value would be 0, indicating no distance between them. This figure increases the more distance there is between the two trees.

#### Test 2: Shimodaira-Hasegawa (SH) statistical test of two trees

For each gene MSA, complete and site-stripped, a comparison of the likelihood of the estimated Bayesian phylogeny for that alignment with the likelihood of its corresponding 'ideal' species tree was carried out using the SH test [14] implemented in TreePuzzle 5.2 [15] to determine which tree was significantly the best-fit tree for the alignment.

### Selective Pressure Analysis

PAML 4.3 [57,58] uses a ML method of calculating ω for site-specific and lineage-site specific changes. Codeml, part of the PAML 4.3 package [57,58], applies a series of models to our data, with each model differing from the previous with the addition of more complex parameters. The simplest model is M0, and it calculates an ω value over the entire alignment. This model assumes that all sites and all lineages are evolving at the same rate. Model M3 is an extension of M0 and allows all *ω *values to vary freely. There are two variations of the M3 model, m3(k = 2) discrete which allows two variable classes of sites and m3(k = 3) which allows three classes of site. M1 is a neutral model that allows two parameter estimates for proportion of sites where ω = 0 or ω = 1. M2 is the selection model, it allows three parameters where ω = 0 or ω = 1 or ω is estimated and free to be greater than 1. M7, is the beta model, it allows ten different site classes for ω between 0 and 1. M7 is compared against the more parameter rich M8 (beta &omega >1). M8 allows 10 different site classes but contains an additional parameter whereby the 11^th ^ω is free to vary between 0 and >1. M8a(beta &omega = 1) is null hypothesis of model 8. Model A & Model B are models that allow testing of ω variation in lineage-site analyses. Model A is an extension of M1 and Model B is a more parameter rich extension of m3(k = 2). We have also implemented model A null which is denoted as modelA1 elsewhere. Model A null is compared to model A in an LRT as per Additional File 9. Only statistically significant models for the data are taken into account. Statistically significant results were decided by calculating the difference in log likelihood or, lnL, scores between models and their more parameter rich extensions in a likelihood ratio test (LRT) as described previously in [17,58]. If the likelihood score was exceeded the critical χ^2 ^values, then the result was significant. See Additional File 9 for full set of LRTs performed.

### In silico analysis of positively selected sites

Sites under positive selection (ω > 1) were estimated using the empirical Bayes methods in the site-specific and lineage specific analysis performed. The methods used were naúve empirical Bayes (NEB) and Bayes empirical Bayes (BEB) [58]. Swiss-Prot is a protein sequence database that provides description of the function of a protein, the domain structures, post-translational modifications and variants. Significant sites, verified through close examination of the MSAs and codeml output using alignment visualisation software Se-AL [51], were compared with unaligned human amino acid sequence taken from Swiss-Prot. These sites were examined to see whether or not they lay in catalytically important regions of the protein.

## Abbreviations

A.A.: Amino Acid; Bck: Background lineage/s; BEB: Bayes Empirical Bayes; CDS: Coding DNA sequence; Dn: Non-synonymous substitution per non-synonymous site; Ds: Synonymous substitution per synonymous site; F: Frequency of amino acids; Fwd: Foreground lineage/s; G: gamma distributed sites rates across sites; GTR: General Time Reversible; I: invariable; JTT: Jones, Taylor and Thornton; LBA: Long Branch Attraction; LM: Likelihood mapping; LRT: Likelihood Ratio Test; ML: Maximum Likelihood; MSA: Multiple Sequence Alignment; N/A: data not available; NB: Non-binary tree; NEB: Naïve Empirical Bayes; NS: No statistical difference; OI: Osteogenesis imperfecta; OI-II/-III/-IV: Osteogenesis imperfecta type -2/-3/-4; P: probability; PP: Posterior Probability; RMSD: Root Mean Squared Deviation; SH: Shimodaira Hasegawa; SNP: Single nucleotide polymorphism.

## Authors' contributions

CCM carried out all data assembly, including searches of (i) literature, (ii) microarray studies, and (iii) protein interaction databases. CCM carried out all homolog identification and MSAs. NBL and CCM carried out all data quality and phylogeny analyses. TAW designed and performed randomization tests, designed bespoke software for the analyses and contributed to the preparation of the manuscript. CCM, NBL and MJO'C carried out all selective pressure analyses. NBL and CCM participated in drafting the manuscript. AJH analysed reproductive age data and genestational times for all mammals in the study, and helped to draft the manuscript. MJO'C conceived of the study, its design and coordination and drafted the manuscript. All authors read and approved the final draft.

## Supplementary Material

Additional file 1 **Additional Table 1 - Results of amino acid composition bias per gene**. Results of the amino acid composition bias test and shown here on a per gene basis. We would expect that if two species have similarly and significantly (P < 0.05) biased amino acid composition that they would be drawn together on the phylogeny. Those with P < 0.05 scores are highlighted but are dispersed throughout different genes. The frequency distribution assumed in the maximum likelihood model calculated by Tree-Puzzle (5% chi-square p-values) was used. N/A = species not represented in the gene dataset. Click here for file

Additional file 2 **Additional Table 2 - Results of base composition bias per gene**. Results of the base composition bias test and shown here on a per gene basis. We would expect that if two species have similarly and significantly (P < 0.05) biased base composition that they would be drawn together on the phylogeny. Those with P < 0.05 scores are highlighted but are dispersed throughout different genes. The frequency distribution assumed in the maximum likelihood model calculated by Tree-Puzzle (5% chi-square p-values) was used. N/A = species not represented in the gene dataset. Click here for file

Additional file 3 **Additional Table 3 - Results of likelihood mapping test for phylogenetic support and conflict estimated for each gene**. Results of Likelihood mapping test are shown here on a gene-by-gene basis. This table summarizes the amount of phylogenetic signal and conflict in each alignment. The three possible topologies for each quartet of species are represented by the corners of the triangle, these corners represent strong support for phylogenetic signal. Quartets present on the vertices represent incongruence in the phylogenetic signal. Quartets at the centre of the triangle represents those quartets where all three topologies are equally likely, i.e. phylogenetic signal completely lacking. Each gene is subsequently given a category based on the quality of the data, only categories 1 and 2 were used. Click here for file

Additional file 4 **Additional Table 4 - Results of root mean squared deviation (RMSD) analysis for comparing binary trees**. This table summarizes the results of comparing the site stripped phylogenies with the ideal species phylogeny. In the first column is the gene name. Each of the subsequent columns represents a category of site variation that is removed (1 is the slowest evolving, 8 the most rapid). The values given for each category removed is the RMSD statistic and represents how similar the resultant site stripped topology is to the canonical species phylogeny. NB - non-binary tree, N/A - not applicable (site category not estimated for alignment). Click here for file

Additional file 5 **Additional Table 5 - Results of the SH test for site-stripped gene versus ideal species phylogeny**. This table summarizes the results of comparing the site stripped phylogenies with the ideal species phylogeny using the SH test, this is a more statistically robust approach and more suited to multi-furcating topologies such as those in the dataset. Each of the rows represents a category of site variation that is removed. For each site stripped site dataset the resultant gene tree is compared to the species phylogeny. The values given for each category removed denotes whether there is a significant difference between the site stripped tree and the species phylogeny, values of less than 0.05 represent those cases where there is a significant difference between the phylogenies. NS = No Statistical significance between gene and species tree, the species tree was taken in these cases. Click here for file

Additional file 6 **Additional Table 6(a-k) - Complete results of Maximum likelihood analysis for selective pressure variation per gene**. For each gene analyzed (a-k) the results are shown in full on a gene-by-gene basis (in alphabetical order). The layout of each table is identical for each gene. The corresponding LRTs performed and all scores and values computed are shown below. The models used are given in the left-most column (Model), followed by the number of parameters associated with that model (P). The Log Likelihood or each model is given in the column (L), and the estimates of the parameters for the proportion of sites (p) and the ratio of Dn/Ds (ω) are given. Sites identified by each model as being positively selected are shown in the final column. Click here for file

Additional file 7 **Additional Table 7 - Summary of data used in the analysis. Species names, unique identifiers and sequence lengths are given for all data**. Summary of data used in the analysis. Species names, unique identifiers for Ensembl (ENS) or Swiss-Prot and database versions are given. The sequence length per species are given for all genes. Click here for file

Additional file 8 **Additional Table 8 - Parameters for Phylogeny Reconstruction per gene**. The parameters used to reconstruct each gene tree in MrBayes are shown. The model of rate heterogeneity for each gene is shown, along with the number of generations required, and the number of markov chains (these values vary based on the size of the dataset). Click here for file

Additional file 9 **Additional Table 9 - Likelihood ratio tests (LRTs) performed using all evolutionary models used in selection analysis**. Details on all likelihood ratio tests performed in the analysis. The models are denoted by their abbreviated names, Model A1 is denoted as Model A null throughout the manuscript. The number of degrees of freedom (df) are shown, this is relevant for the chi-squared test for significance, the critical values in each instance are given in the final column. Click here for file
