# Erratum to: Positive selection neighboring functionally essential sites and disease-implicated regions of mammalian reproductive proteins

**DOI:** 10.1186/s12862-017-1015-y

**Published:** 2017-07-21

**Authors:** Claire C Morgan, Noeleen B Loughran, Thomas A Walsh, Alan J Harrison, Mary J O’Connell

**Affiliations:** Bioinformatics and Molecular Evolution Group, School of Biotechnology, Dublin City University, Glasnevin, Dublin 9, Ireland

## Erratum

After publication of the original article [1] the authors found that all instances of the protein **SP56** should have been referred to as **SELENBP1** in the article.

The authors wish to clarify that while SP56 is a known synonym for SELENBP1, the gene referred to throughout the manuscript as SP56 is better referred to as SELENBP1 and not the gene for sperm binding protein number 56 (SP56).
