# Supplementary material for: Positive selection neighboring functionally essential sites and disease-implicated regions of mammalian reproductive proteins
Source: BMC Evol Biol. 2010 Feb 11;10:39. doi: 10.1186/1471-2148-10-39 (PMC2830953; doi:10.1186/1471-2148-10-39)
Supplement: Additional file 1 — Additional Table 1 - Results of amino acid composition bias per gene. Results of the amino acid composition bias test and shown here on a per gene basis. We would expect that if two species have similarly and significantly (P < 0.05) biased amino acid composition that they would be drawn together on the phylogeny. Those with P < 0.05 scores are highlighted but are dispersed throughout different genes. The frequency distribution assumed in the maximum likelihood model calculated by Tree-Puzzle (5% chi-square p-values) was used. N/A = species not represented in the gene dataset. [file 1471-2148-10-39-S1.DOC]

**Additional Table 1: Results of amino acid composition bias per gene.**

| **TAXA** | **GENE** | | | | | | | | | | |
| --- | --- | --- | --- | --- | --- | --- | --- | --- | --- | --- | --- |
| **Species** | **Adam2** | **Catsper1**  **Exon1** | **Catsper1**  **Mammals** | **Col1a1** | **Ph20** | **Porimin** | **Prkar2a** | **Semg2** | **Sp56** | **Zp2** | **Zp3** |
| **Human** | 99.99 | 99.99 | N/A | 100.00 | 100.00 | 99.97 | 100.00 | 99.98 | 100.00 | 99.99 | 100.00 |
| **Chimp** | 99.98 | 100.00 | N/A | 100.00 | 99.98 | 100.00 | 100.00 | 99.99 | 100.00 | 99.97 | 100.00 |
| **(Bornean) Orangutan** | 99.99 | 99.90 | N/A | 100.00 | 99.89 | 97.98 | 100.00 | 100.00 | 100.00 | 99.93 | N/A |
| **Macaque** | 99.99 | 99.51 | N/A | N/A | 99.92 | 99.98 | 100.00 | 94.99 | N/A | 100.00 | 100.00 |
| **Mouse** | 99.99 | N/A | 73.79 | 100.00 | N/A | 97.32 | 100.00 | N/A | N/A | 86.86 | N/A |
| **Guinea Pig** | 99.64 | N/A | N/A | N/A | 81.63 | N/A | 97.97 | N/A | N/A | N/A | N/A |
| **Rat** | 99.59 | N/A | 7.22 | 97.80 | N/A | 45.44 | 100.00 | N/A | 99.93 | 53.15 | 71.67 |
| **Horse** | 99.75 | N/A | 58.59 | N/A | N/A | 67.66 | N/A | N/A | 99.98 | 99.97 | 98.91 |
| **Rabbit** | 99.26 | N/A | N/A | N/A | 99.28 | N/A | 95.84 | N/A | 99.94 | 99.91 | N/A |
| **Bonnet Monkey** | N/A | N/A | N/A | N/A | N/A | N/A | N/A | N/A | N/A | N/A | 99.93 |
| **Baboon** | N/A | 99.99 | N/A | N/A | N/A | N/A | N/A | N/A | N/A | N/A | N/A |
| **Hedgehog** | 99.91 | N/A | N/A | N/A | N/A | 94.88 | 99.98 | N/A | N/A | N/A | N/A |
| **Woolly Monkey** | N/A | 97.86 | N/A | N/A | N/A | N/A | N/A | N/A | N/A | N/A | N/A |
| **Elephant** | 99.84 | N/A | N/A | N/A | 95.85 | N/A | 99.94 | N/A | 99.99 | 89.53 | 81.24 |
| **Cow** | 99.92 | N/A | 99.04 | 100.00 | N/A | N/A | 100.00 | N/A | 100.00 | 99.98 | 99.64 |
| **Pika** | N/A | N/A | **3.02** | 99.99 | 99.86 | N/A | 100.00 | N/A | 100.00 | 98.67 | N/A |
| **Dog** | N/A | N/A | 59.49 | 100.00 | N/A | N/A | 100.00 | N/A | 100.00 | 99.99 | 94.15 |
| **Cat** | N/A | N/A | 69.74 | **0.10** | N/A | N/A | 99.94 | N/A | 99.94 | 99.63 | 99.38 |
| **Opossum** | N/A | N/A | N/A | 99.87 | 75.74 | 96.13 | N/A | N/A | N/A | N/A | N/A |
| **Marmoset (Callithrix)** | N/A | N/A | N/A | N/A | N/A | N/A | N/A | N/A | N/A | 99.92 | 99.93 |
| **Platypus** | N/A | N/A | N/A | N/A | 96.12 | N/A | N/A | N/A | N/A | N/A | N/A |
| **Treeshrew** | N/A | N/A | 81.50 | N/A | 98.37 | N/A | 99.90 | N/A | 100.00 | 99.80 | 99.99 |
| **Bushbaby** | N/A | N/A | N/A | N/A | N/A | N/A | 98.01 | N/A | 99.66 | 92.36 | N/A |
| **Shrew** | N/A | N/A | N/A | N/A | N/A | 57.99 | 99.98 | N/A | 85.00 | 98.45 | N/A |
| **(Black and White) Colobous** | N/A | 99.57 | N/A | N/A | N/A | N/A | N/A | 99.99 | N/A | N/A | N/A |
| **Gorilla** | N/A | 99.99 | N/A | N/A | N/A | N/A | N/A | 100.00 | N/A | N/A | N/A |
| **Kloss Gibbon** | N/A | N/A | N/A | N/A | N/A | N/A | N/A | 99.98 | N/A | N/A | N/A |
| **Squirrel** | N/A | N/A | N/A | N/A | N/A | N/A | N/A | N/A | N/A | 99.99 | N/A |
| **Pig** | N/A | N/A | N/A | N/A | N/A | N/A | N/A | N/A | N/A | N/A | 99.44 |
| **Common**  **Gibbon** | N/A | N/A | N/A | N/A | N/A | N/A | N/A | 99.77 | N/A | N/A | N/A |
| **Crab-eating**  **Macaque** | N/A | N/A | N/A | N/A | N/A | N/A | N/A | 98.67 | N/A | N/A | N/A |
| **Pig-tailed**  **Langur** | N/A | N/A | N/A | N/A | N/A | N/A | N/A | 99.96 | N/A | N/A | N/A |
| **Bonobo** | N/A | 100.00 | N/A | N/A | N/A | N/A | N/A | 100.00 | N/A | N/A | N/A |
| **Sumatran**  **Orangutan** | N/A | N/A | N/A | N/A | N/A | N/A | N/A | 100.00 | N/A | N/A | N/A |
| **Spider Monkey** | N/A | 96.23 | N/A | N/A | N/A | N/A | N/A | N/A | N/A | N/A | N/A |
| **Ring-tailed Lemur** | N/A | 46.02 | N/A | N/A | N/A | N/A | N/A | N/A | N/A | N/A | N/A |
| **Green Monkey** | N/A | 99.90 | N/A | N/A | N/A | N/A | N/A | N/A | N/A | N/A | N/A |
| **Talapoin** | N/A | 99.11 | N/A | N/A | N/A | N/A | N/A | N/A | N/A | N/A | N/A |
| **Squirrel Monkey** | N/A | 62.69 | N/A | N/A | N/A | N/A | N/A | N/A | N/A | N/A | N/A |
| **Night Monkey** | N/A | 93.93 | N/A | N/A | N/A | N/A | N/A | N/A | N/A | N/A | N/A |
| **Cotton Top**  **Tamarin** | N/A | 90.63 | N/A | N/A | N/A | N/A | N/A | N/A | N/A | N/A | N/A |
| **TOTAL TAXA**  **/GENE** | 12 | 16 | 8 | 10 | 11 | 10 | 17 | 12 | 14 | 18 | 13 |
