# Supplementary material for: Positive selection neighboring functionally essential sites and disease-implicated regions of mammalian reproductive proteins
Source: BMC Evol Biol. 2010 Feb 11;10:39. doi: 10.1186/1471-2148-10-39 (PMC2830953; doi:10.1186/1471-2148-10-39)
Supplement: Additional file 2 — Additional Table 2 - Results of base composition bias per gene. Results of the base composition bias test and shown here on a per gene basis. We would expect that if two species have similarly and significantly (P < 0.05) biased base composition that they would be drawn together on the phylogeny. Those with P < 0.05 scores are highlighted but are dispersed throughout different genes. The frequency distribution assumed in the maximum likelihood model calculated by Tree-Puzzle (5% chi-square p-values) was used. N/A = species not represented in the gene dataset. [file 1471-2148-10-39-S2.DOC]

**Additional Table 2: Results of base composition per gene.**

| **TAXA** | **GENE** | | | | | | | | | | |
| --- | --- | --- | --- | --- | --- | --- | --- | --- | --- | --- | --- |
| **Species** | **Adam2** | **Catsper1**  **Exon1** | **Catsper1**  **Mammals** | **Col1a1** | **Ph20** | **Porimin** | **Prkar2a** | **Semg2** | **Sp56** | **Zp2** | **Zp3** |
| **Human** | 13.93 | 98.52 | N/A | 85.85 | 80.08 | 92.27 | 73.84 | 98.81 | 92.88 | 29.16 | 98.77 |
| **Chimp** | 18.43 | 99.32 | N/A | 88.94 | 72.20 | 85.95 | 63.73 | 99.85 | 90.49 | 34.65 | 84.09 |
| **(Bornean)**  **Orangutan** | 12.27 | 98.69 | N/A | 87.45 | 36.98 | 5.74 | 66.81 | 99.17 | 96.80 | 63.74 | 98.77 |
| **Macaque** | 30.61 | 99.16 | N/A | N/A | 96.76 | 96.62 | 79.30 | 94.87 | N/A | 67.09 | 94.38 |
| **Mouse** | **0.00** | N/A | 56.86 | 17.58 | N/A | 31.03 | 92.97 | N/A | N/A | 96.20 | N/A |
| **Guinea Pig** | **1.39** | N/A | N/A | N/A | 75.14 | N/A | 13.88 | N/A | N/A | N/A | N/A |
| **Rat** | **0.00** | N/A | 5.73 | 5.03 | N/A | **0.51** | 93.65 | N/A | **3.83** | 43.17 | 44.42 |
| **Horse** | 27.97 | N/A | 7.40 | N/A | N/A | **0.85** | N/A | N/A | 99.55 | 98.21 | 38.75 |
| **Rabbit** | 92.11 | N/A | N/A | N/A | 34.00 | N/A | **1.21** | N/A | 5.58 | 96.54 | N/A |
| **Bonnet Monkey** | N/A | N/A | N/A | N/A | N/A | N/A | 95.84 | N/A | N/A | N/A | 98.56 |
| **Baboon** | N/A | 99.02 | N/A | N/A | N/A | N/A | 95.84 | N/A | N/A | N/A | N/A |
| **Hedgehog** | 10.85 | N/A | N/A | N/A | N/A | 25.51 | **4.99** | N/A | N/A | N/A | N/A |
| **Woolly Monkey** | N/A | 94.93 | N/A | N/A | N/A | N/A | N/A | N/A | N/A | N/A | N/A |
| **Elephant** | 28.93 | N/A | N/A | N/A | 68.92 | N/A | 6.05 | N/A | 94.41 | 60.56 | 89.55 |
| **Cow** | 58.72 | N/A | 85.30 | 28.74 | N/A | N/A | 71.85 | N/A | 97.18 | 86.75 | 24.40 |
| **Pika** | N/A | N/A | 15.78 | 99.14 | 80.92 | N/A | 98.91 | N/A | 92.85 | 56.88 | N/A |
| **Dog** | N/A | N/A | 45.31 | 69.29 | N/A | N/A | 72.37 | N/A | 91.35 | 57.55 | 28.67 |
| **Cat** | N/A | N/A | 78.89 | **0.00** | N/A | N/A | 6.05 | N/A | 99.50 | 37.76 | 85.23 |
| **Opossum** | N/A | N/A | N/A | **0.00** | 16.00 | **4.13** | N/A | N/A | N/A | N/A | N/A |
| **Marmoset (Callithrix)** | N/A | N/A | N/A | N/A | N/A | N/A | N/A | N/A | N/A | 52.30 | 98.56 |
| **Platypus** | N/A | N/A | N/A | N/A | **0.00** | N/A | N/A | N/A | N/A | N/A | N/A |
| **Treeshrew** | N/A | N/A | 41.68 | N/A | 65.23 | N/A | **0.35** | N/A | 38.00 | 44.65 | 97.98 |
| **Bushbaby** | N/A | N/A | N/A | N/A | N/A | N/A | 25.07 | N/A | 85.44 | 40.10 | N/A |
| **Shrew** | N/A | N/A | N/A | N/A | N/A | 25.31 | 10.14 | N/A | **3.23** | 98.79 | N/A |
| **(Black and White )**  **Colobous** | N/A | 86.06 | N/A | N/A | N/A | N/A | N/A | 99.55 | N/A | N/A | N/A |
| **Gorilla** | N/A | 94.50 | N/A | N/A | N/A | N/A | N/A | 99.77 | N/A | N/A | N/A |
| **Kloss Gibbon** | N/A | N/A | N/A | N/A | N/A | N/A | N/A | 99.95 | N/A | N/A | N/A |
| **Squirrel** | N/A | N/A | N/A | N/A | N/A | N/A | N/A | N/A | N/A | 99.58 | N/A |
| **Pig** | N/A | N/A | N/A | N/A | N/A | N/A | N/A | N/A | N/A | N/A | 21.02 |
| **Common Gibbon** | N/A | N/A | N/A | N/A | N/A | N/A | N/A | 99.85 | N/A | N/A | N/A |
| **Crab-eating**  **Macaque** | N/A | N/A | N/A | N/A | N/A | N/A | N/A | 99.63 | N/A | N/A | N/A |
| **Pig-tailed Langur** | N/A | N/A | N/A | N/A | N/A | N/A | N/A | 99.39 | N/A | N/A | N/A |
| **Bonobo** | N/A | 99.32 | N/A | N/A | N/A | N/A | N/A | 99.59 | N/A | N/A | N/A |
| **Sumatran**  **Orangutan** | N/A | N/A | N/A | N/A | N/A | N/A | N/A | 99.17 | N/A | N/A | N/A |
| **Spider Monkey** | N/A | 93.11 | N/A | N/A | N/A | N/A | N/A | N/A | N/A | N/A | N/A |
| **Ring-tailed Lemur** | N/A | 11.18 | N/A | N/A | N/A | N/A | N/A | N/A | N/A | N/A | N/A |
| **Green Monkey** | N/A | 96.53 | N/A | N/A | N/A | N/A | N/A | N/A | N/A | N/A | N/A |
| **Talapoin** | N/A | 99.72 | N/A | N/A | N/A | N/A | N/A | N/A | N/A | N/A | N/A |
| **Squirrel Monkey** | N/A | 70.25 | N/A | N/A | N/A | N/A | N/A | N/A | N/A | N/A | N/A |
| **Night Monkey** | N/A | 99.13 | N/A | N/A | N/A | N/A | N/A | N/A | N/A | N/A | N/A |
| **Cotton Top**  **Tamarin** | N/A | 97.79 | N/A | N/A | N/A | N/A | N/A | N/A | N/A | N/A | N/A |
| **TOTAL TAXA/**  **GENE** | 12 | 16 | 8 | 10 | 11 | 10 | 19 | 12 | 14 | 18 | 14 |
