# Supplementary material for: Positive selection neighboring functionally essential sites and disease-implicated regions of mammalian reproductive proteins
Source: BMC Evol Biol. 2010 Feb 11;10:39. doi: 10.1186/1471-2148-10-39 (PMC2830953; doi:10.1186/1471-2148-10-39)
Supplement: Additional file 3 — Additional Table 3 - Results of likelihood mapping test for phylogenetic support and conflict estimated for each gene. Results of Likelihood mapping test are shown here on a gene-by-gene basis. This table summarizes the amount of phylogenetic signal and conflict in each alignment. The three possible topologies for each quartet of species are represented by the corners of the triangle, these corners represent strong support for phylogenetic signal. Quartets present on the vertices represent incongruence in the phylogenetic signal. Quartets at the centre of the triangle represents those quartets where all three topologies are equally likely, i.e. phylogenetic signal completely lacking. Each gene is subsequently given a category based on the quality of the data, only categories 1 and 2 were used. [file 1471-2148-10-39-S3.DOC]

**Additional Table 3: Results of Likelihood mapping test for phylogenetic support and conflict estimated for each gene**.


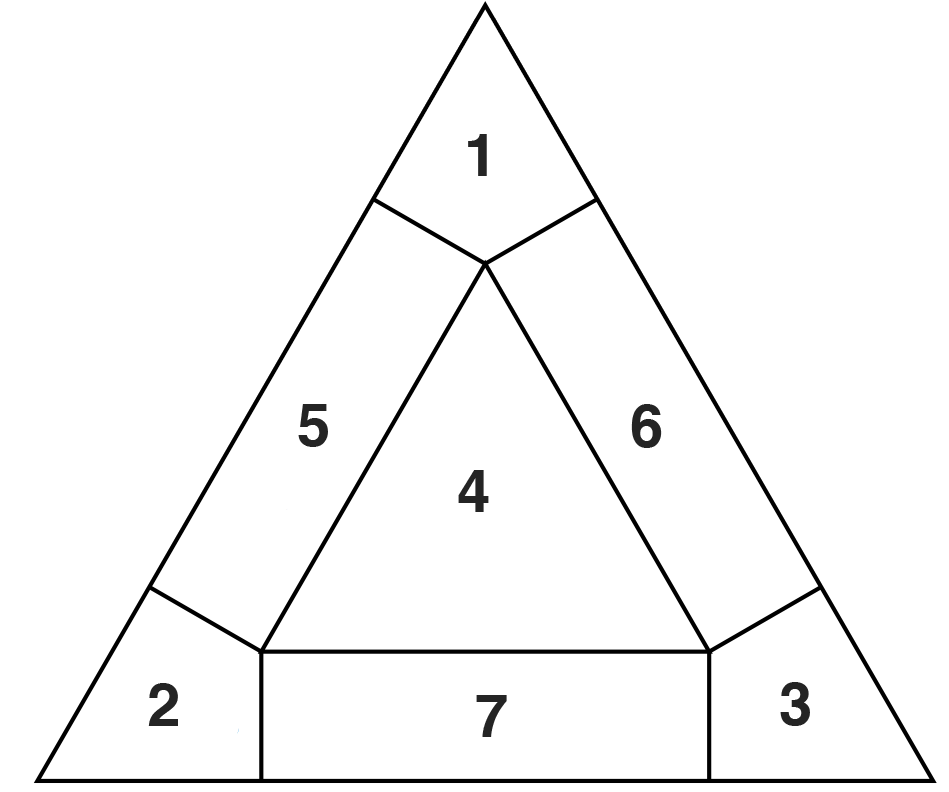


| **Gene** | **Likelihood mapping quadrant (see above)** | | | | | | | **Category** |
| --- | --- | --- | --- | --- | --- | --- | --- | --- |
|  | **1** | **2** | **3** | **4** | **5** | **6** | **7** |  |
| Adam2 | 22.9 | 28.6 | 40.0 | 5.7 | 0.0 | 2.9 | 0.0 | 1 |
| Bag2 | 18.1 | 14.3 | 21.4 | 38.1 | 3.3 | 1.9 | 2.9 | 3 |
| Catsper1 | 34.3 | 42.9 | 22.9 | 0.0 | 0.0 | 0.0 | 0.0 | 1 |
| Cd9 | 11.9 | 11.4 | 6.7 | 55.7 | 6.7 | 4.3 | 3.3 | 3 |
| Col1a1 | 28.6 | 30.0 | 37.1 | 4.3 | 0.0 | 0.0 | 0.0 | 1 |
| Glo1 | 26.2 | 23.0 | 27.8 | 15.9 | 3.2 | 0.8 | 3.2 | 3 |
| gmnn | 19.0 | 25.2 | 21.0 | 21.4 | 1.9 | 7.1 | 4.3 | 3 |
| Gmps | 19.0 | 15.2 | 20.0 | 24.8 | 6.7 | 5.7 | 8.6 | 3 |
| Hsd17b4 | 15.1 | 30.2 | 26.2 | 15.9 | 3.2 | 5.6 | 4.0 | 3 |
| lpaat-e | 23.0 | 27.8 | 27.8 | 17.5 | 3.2 | 0.8 | 0.0 | 3 |
| Pak1ip1 | 19.0 | 24.6 | 20.6 | 19.0 | 7.1 | 3.2 | 6.3 | 3 |
| Ph20 | 36.5 | 25.4 | 33.3 | 4.0 | 0.8 | 0.0 | 0.0 | 1 |
| Porimin | 18.6 | 32.9 | 35.7 | 1.4 | 4.3 | 4.3 | 2.9 | 1 |
| Prkar2a | 27.3 | 29.7 | 27.3 | 4.8 | 3.3 | 3.6 | 3.9 | 2 |
| Prm1 | 11.4 | 15.7 | 24.3 | 41.4 | 1.4 | 1.4 | 4.3 | 3 |
| Prm2 | 27.0 | 23.8 | 22.2 | 15.9 | 5.6 | 3.2 | 2.4 | 3 |
| Semg2 | 27.1 | 34.3 | 28.6 | 7.1 | 0.0 | 1.4 | 1.4 | 1 |
| Serpine2 | 28.6 | 25.7 | 21.0 | 16.7 | 3.3 | 1.4 | 3.3 | 3 |
| Slc39a6 | 21.4 | 24.6 | 23.8 | 22.2 | 2.4 | 3.2 | 2.4 | 3 |
| slpi | 28.6 | 17.1 | 34.3 | 17.1 | 0.0 | 2.9 | 0.0 | 3 |
| Sp56 | 32.9 | 24.3 | 21.4 | 14.3 | 4.3 | 0.0 | 2.9 | 2 |
| Spa17 | 27.8 | 19.8 | 19.0 | 23.0 | 0.8 | 7.9 | 1.6 | 3 |
| tfap2c | 25.4 | 23.8 | 17.5 | 28.6 | 2.4 | 0.8 | 1.6 | 3 |
| Twsg1 | 23.8 | 23.8 | 30.2 | 13.5 | 1.6 | 4.0 | 3.2 | 3 |
| Vmp1 | 30.2 | 25.4 | 23.0 | 8.7 | 4.0 | 3.2 | 5.6 | 3 |
| Zp2 | 32.9 | 24.3 | 37.1 | 2.9 | 1.4 | 0.0 | 1.4 | 1 |
| Zp3 | 29.5 | 31.7 | 29.2 | 3.9 | 2.1 | 1.4 | 2.1 | 1 |
