# Supplementary material for: Positive selection neighboring functionally essential sites and disease-implicated regions of mammalian reproductive proteins
Source: BMC Evol Biol. 2010 Feb 11;10:39. doi: 10.1186/1471-2148-10-39 (PMC2830953; doi:10.1186/1471-2148-10-39)
Supplement: Additional file 4 — Additional Table 4 - Results of root mean squared deviation (RMSD) analysis for comparing binary trees. This table summarizes the results of comparing the site stripped phylogenies with the ideal species phylogeny. In the first column is the gene name. Each of the subsequent columns represents a category of site variation that is removed (1 is the slowest evolving, 8 the most rapid). The values given for each category removed is the RMSD statistic and represents how similar the resultant site stripped topology is to the canonical species phylogeny. NB - non-binary tree, N/A - not applicable (site category not estimated for alignment). [file 1471-2148-10-39-S4.DOC]

**Additional Table 4: Results of root mean squared deviation (RMSD) analysis for comparing binary trees.**

| **Gene** | **Complete MSA** | **8** | **7** | **6** | **5** | **4** | **3** | **2** | **1** | **8+1** |
| --- | --- | --- | --- | --- | --- | --- | --- | --- | --- | --- |
| **Adam2** | 1.2247 | 0.7282 | 0.9847 | NB | NB | NB | NB | N/A | 1.2247 | 0.7282 |
| **CatE** | NB | NB | NB | NB | NB | NB | NB | NB | NB | NB |
| **CatM** | 0.0000 | 0.0000 | 0.0000 | 0.6547 | NB | NB | NB | NB | 0.0000 | 0.0000 |
| **Col1a1** | 1.4063 | NB | NB | N/A | N/A | N/A | N/A | N/A | NB | NB |
| **Ph20** | 1.0617 | 1.0617 | 1.0617 | 0.0000 | NB | NB | NB | NB | 1.0617 | 1.0617 |
| **Porimin** | NB | NB | 1.1547 | NB | NB | NB | NB | NB | NB | NB |
| **Prkar2a** | NB | NB | NB | NB | N/A | N/A | N/A | N/A | NB | NB |
| **Semg2** | NB | NB | NB | N/A | N/A | N/A | N/A | N/A | NB | NB |
| **Sp56** | NB | NB | NB | NB | N/A | N/A | N/A | N/A | NB | NB |
| **ZP2** | NB | NB | NB | NB | NB | NB | NB | NB | NB | NB |
| **ZP3** | NB | NB | NB | NB | NB | NB | NB | N/A | NB | NB |
