# Supplementary material for: Positive selection neighboring functionally essential sites and disease-implicated regions of mammalian reproductive proteins
Source: BMC Evol Biol. 2010 Feb 11;10:39. doi: 10.1186/1471-2148-10-39 (PMC2830953; doi:10.1186/1471-2148-10-39)
Supplement: Additional file 5 — Additional Table 5 - Results of the SH test for site-stripped gene versus ideal species phylogeny. This table summarizes the results of comparing the site stripped phylogenies with the ideal species phylogeny using the SH test, this is a more statistically robust approach and more suited to multi-furcating topologies such as those in the dataset. Each of the rows represents a category of site variation that is removed. For each site stripped site dataset the resultant gene tree is compared to the species phylogeny. The values given for each category removed denotes whether there is a significant difference between the site stripped tree and the species phylogeny, values of less than 0.05 represent those cases where there is a significant difference between the phylogenies. NS = No Statistical significance between gene and species tree, the species tree was taken in these cases. [file 1471-2148-10-39-S5.DOC]

**Additional Table 5: Results of the SH tests for site-stripped gene versus ideal species phylogeny.**

| **Site Category Removed (gene)** | **SH – gene** | **SH - ideal** | **Best-fit Tree** |
| --- | --- | --- | --- |
| Adam2 | | | |
| 8 | 1.0000 | 0.0970 | NS |
| 7 | 1.0000 | 0.1740 | NS |
| 6 | 0.3280 | 1.0000 | NS |
| 5 | 1.0000 | 0.2590 | NS |
| 4 | 1.0000 | 0.0880 | NS |
| 3 | 1.0000 | 0.0000 | gene |
| 1 | 1.0000 | 0.1040 | NS |
| 81 | 1.0000 | 0.0930 | NS |
| Catsper1 Exon1 | | | |
| 8 | 1.0000 | 0.0710 | NS |
| 7 | 1.0000 | 0.1180 | NS |
| 6 | 1.0000 | 0.1200 | NS |
| 5 | 1.0000 | 0.1170 | NS |
| 4 | 1.0000 | 0.1020 | NS |
| 3 | 1.0000 | 0.2140 | NS |
| 2 | 1.0000 | 0.0000 | gene |
| 1 | 1.0000 | 0.1220 | NS |
| 81 | 1.0000 | 0.0760 | NS |
| Catsper1 Mammals | | | |
| 8 | 1.0000 | 0.5060 | NS |
| 7 | 0.5020 | 1.0000 | NS |
| 6 | 1.0000 | 0.4270 | NS |
| 5 | 1.0000 | 0.1710 | NS |
| 4 | 1.0000 | 0.0000 | gene |
| 3 | 1.0000 | 0.0000 | gene |
| 2 | 1.0000 | 0.0000 | gene |
| 1 | 0.4940 | 1.0000 | NS |
| 81 | 1.0000 | 0.4900 | NS |
| Col1a1 | | | |
| 8 | 1.0000 | 0.0580 | NS |
| 7 | 1.0000 | 0.0000 | gene |
| 1 | 1.0000 | 0.0830 | NS |
| 81 | 1.0000 | 0.0660 | NS |
| Ph20 | | | |
| 8 | 1.0000 | 0.1960 | NS |
| 7 | 1.0000 | 0.2700 | NS |
| 6 | 0.4930 | 1.0000 | NS |
| 5 | 1.0000 | 0.0000 | gene |
| 4 | 1.0000 | 0.0530 | NS |
| 3 | 1.0000 | 0.0000 | gene |
| 2 | 1.0000 | 0.0000 | gene |
| 1 | 1.0000 | 0.3020 | NS |
| 81 | 1.0000 | 0.1560 | NS |
| Porimin | | | |
| 8 | 1.0000 | 0.2180 | NS |
| 7 | 1.0000 | 0.1120 | NS |
| 6 | 1.0000 | 0.2210 | NS |
| 5 | 1.0000 | 0.2140 | NS |
| 4 | 1.0000 | 0.1280 | NS |
| 3 | 1.0000 | 0.1510 | NS |
| 2 | 1.0000 | 0.0000 | gene |
| 1 | 0.1450 | 1.0000 | NS |
| 81 | 1.0000 | 0.3990 | NS |
| Prkar2a | | | |
| 8 | 1.0000 | 0.2340 | NS |
| 7 | 1.0000 | 0.0770 | NS |
| 6 | 1.0000 | 0.0000 | gene |
| 1 | 1.0000 | 0.0650 | NS |
| 81 | 1.0000 | 0.1140 | NS |
| Semg2 | | | |
| 8 | 1.0000 | 0.0380 | gene |
| 7 | 1.0000 | 0.0000 | gene |
| 1 | 1.0000 | 0.0970 | NS |
| 81 | 1.0000 | 0.0440 | gene |
| Sp56 | | | |
| 8 | 1.0000 | 0.1950 | NS |
| 7 | 1.0000 | 0.1350 | NS |
| 6 | 1.0000 | 0.0000 | gene |
| 1 | 1.0000 | 0.2470 | NS |
| 81 | 1.0000 | 0.2220 | NS |
| Zp2 | | | |
| 8 | 1.0000 | 0.1110 | NS |
| 7 | 1.0000 | 0.1370 | NS |
| 6 | 1.0000 | 0.2960 | NS |
| 5 | 1.0000 | 0.3460 | NS |
| 4 | 0.1590 | 1.0000 | NS |
| 3 | 1.0000 | 0.2210 | NS |
| 2 | 1.0000 | 0.0000 | gene |
| 1 | 0.0000 | 1.0000 | NS |
| 81 | 1.0000 | 0.1340 | NS |
| Zp3 | | | |
| 8 | 1.0000 | 0.0210 | NS |
| 7 | 1.0000 | 0.0190 | NS |
| 6 | 1.0000 | 0.0560 | NS |
| 5 | 1.0000 | 0.1240 | NS |
| 4 | 0.0910 | 1.0000 | NS |
| 3 | 1.0000 | 0.0000 | gene |
| 1 | 1.0000 | 0.0050 | gene |
| 81 | 1.0000 | 0.0280 | gene |
