# Supplementary material for: Positive selection neighboring functionally essential sites and disease-implicated regions of mammalian reproductive proteins
Source: BMC Evol Biol. 2010 Feb 11;10:39. doi: 10.1186/1471-2148-10-39 (PMC2830953; doi:10.1186/1471-2148-10-39)
Supplement: Additional file 6 — Additional Table 6(a-k) - Complete results of Maximum likelihood analysis for selective pressure variation per gene. For each gene analyzed (a-k) the results are shown in full on a gene-by-gene basis (in alphabetical order). The layout of each table is identical for each gene. The corresponding LRTs performed and all scores and values computed are shown below. The models used are given in the left-most column (Model), followed by the number of parameters associated with that model (P). The Log Likelihood or each model is given in the column (L), and the estimates of the parameters for the proportion of sites (p) and the ratio of Dn/Ds (ω) are given. Sites identified by each model as being positively selected are shown in the final column. [file 1471-2148-10-39-S6.DOC]

**Additional Table 6: Complete set of Paml results for all models tested, LRTs are given beneath each table, results are shown on a gene by gene basis.**

**(a) Complete set of estimates for Adam2 from codeml:**

| **Model** | **P** | **Estimates of parameters** | **Positively**  **selected sites** |
| --- | --- | --- | --- |
| M0 : one ratio | 1 | w = 0.3874 | None |
| **Site-specific:**  M1:Neutral | 2 | p_0_= 0.58406 , w_0_<1 | Not allowed |
| M2:Selection | 4 | p_0_=0.54635, p_1_= 0.38914  (p_2_= 0.06451), w_0_<1, w_1_ = 1, w_2_ =  5.14527 | **NEB**: 45>0.50, 18>0.95  5>0.99 **BEB:** 59>0.50,  18>0.95 5>0.99 |
| M3:Discrete(K = 2) | 3 | p_0_=  0.59766 , (p_1_= 0.40234)  w_0_= 0.11485, w_1_= 1.08488 | **NEB**:303> 0.50, 201>0.95, 159>0.99 |
| M3:Discrete(K = 3) | 5 | p_0_=0.46598, p_1_= 0.44659, (p_2_= 0.08744)  w_0_=0.06444, w_1_=0.67760, w_2_= 3.49724 | **NEB:** 304>0.50 201>0.95, 159>0.99 |
| M7: Beta | 2 | p= 0.32715, q= 0.47096 | Not allowed |
| M8: Beta&Omega > 1 | 4 | p_0_=0.92632  , p =0.37637 , q =  0.60688  (p_1_=0.07368 ), w =3.94326 | **NEB:** 51>0.50,22>0.95, 6>0.99 **BEB:** 45>0.50, 15>0.95, 5>0.99 |
| M8a: Beta&Omega = 1 | 3 | p_0_=0.69927, p =0.63216 , q =2.94862  (p_1_=0.30073 ), w = 1 | Not allowed |
| **Branch-specific: Human** |  |  |  |
| Model A | 4 | p_0_ = 0.55158, p_1_ =0.39114,  (p_2_ = 0.03352 , p_3_ = 0.02377),  w_0_ = 0.10557 w_1_ = 1, w_2_ = 3.40857 | **NEB:** 1>0.50, 0>0.95, 0>0.99 **BEB:** 1>0.50, 0>0.95, 0>0.99 |
| Model A null | 3 | p_0_ = 0.50204 , p_1_ =0.35622,  (p_2_ =  0.08291 , p_3_ = 0.05883), w_0_ = 0.10555, w_1_ = 1, w_2_ = 1 | Not allowed |
| Model B | 5 | p_0_ =  0.55732, p_1_ = 0.37303,  (p_2_ = 0.04173 ,p_3_ = 0.02793),  w_0_ = 0.11517, w_1_ = 1.08741, w_2_ = 3.25405 | **NEB:** 1>0.50, 0>0.95, 0>0.99 |
| **Branch-specific: Chimp** |  |  |  |
| Model A | 4 | p_0_ = 0.42038, p_1_ =  0.29767,  (p_2_ = 0.16507, p_3_ = 0.11689),  w_0_ =  0.10516 , w_1_ = 1, w_2_ =1.58791 | **NEB:** 5>0.50, 0>0.95, 0>0.99 **BEB:** 5>0.50, 0>0.95, 0>0.99 |
| Model A null | 3 | p_0_ =   0.34053 , p_1_ = 0.24107,  (p_2_ = 0.24497, p_3_ = 0.17342),  w_0_ =  0.10518, w_1_ = 1, w_2_ = 1 | Not allowed |
| Model B | 5 | p_0_ = 0.38462, p_1_ = 0.25656,  (p_2_ = 0.21524, p_3_ = 0.14358),  w_0_ =  0.11500, w_1_ =1.08919 , w_2_ = 1.35250 | **NEB:** 5>0.50 |
| **Branch-specific: Macaque** |  |  |  |
| Model A | 4 | p_0_ = 0.52835, p_1_ =0.37595 ,  (p_2_ = 0.05591, p_3_ = 0.03979), w_0_ = 0.10376, w_1_ = 1, w_2_ =1.71397 | **NEB:** 9>0.50  **BEB**: 2>0.50 |
| Model A null | 3 | p_0_ = 0.49793, p_1_ = 0.35442 ,  (p_2_ = 0.08625 , p_3_ = 0.06139), w_0_ =0.10375 , w_1_ = 1, w_2_ = 1 | Not allowed |
| Model B | 5 | p_0_ = 0.52879 , p_1_ =0.35559, (p_2_ =0.06913 , p_3_ = 0.04649),  w_0_ = 0.11307, w_1_ = 1.08474, w_2_ = 1.42198 | **NEB:** 9>0.50 |
| **Branch-specific: Mouse** |  |  |  |
| Model A | 4 | p_0_ =  0.58406, p_1_ = 0.41594,  (p_2_ = **0**, p_3_ = 0), w_0_ =  0.10547, w_1_ = 1, w_2_ = 1 | **NEB:** None, **BEB**: None |
| Model A null | 3 | p_0_ = 0.58406, p_1_ =0.41594,  (p_2_ = 0, p_3_ = 0), w_0_ =  0.10547, w_1_ = 1, w_2_ = 1 | Not allowed |
| Model B | 5 | p_0_ = 0.43939, p_1_ =  0.29554,  (p_2_ = 0.15848, p_3_ =0.10659),  w_0_ = 0.11674, w_1_ = 1.10607, w_2_ =0 | **NEB:** None |
| **Branch-specific: Primates** |  |  |  |
| Model A | 4 | p_0_ =  0.58406, p_1_ =0.41594,  (p_2_ = 0, p_3_ = 0), w_0_ =  0.10547 , w_1_ = 1, w_2_ = 1 | **NEB:** None, **BEB:** None |
| Model A null | 3 | p_0_ =0.58406, p_1_ = 0.41594,  (p_2_ = 0, p_3_ = 0), w_0_ =  0.10547, w_1_ = 1, w_2_ = 1 | Not allowed |
| Model B | 5 | p_0_ = 0.59766 , p_1_ = 0.40234,  (p_2_ = 0.00, p_3_ = 0),   w_0_ = 0.11485 , w_1_ =  1.08489, w_2_ = 0 | **NEB:** None |
| **Branch-specific: Rodents** |  |  |  |
| Model A | 4 | p_0_ = 0.58406, p_1_ = 0.41594,  (p_2_ = 0.00, p_3_ =  0.00), w_0_ = 0.10547, w_1_ = 1, w_2_ = 1 | **NEB:** None, **BEB:** None |
| Model A null | 3 | p_0_ = 0.58406, p_1_ = 0.41594,  (p_2_ = 0, p_3_ = 0), w_0_ =  0.10547, w_1_ = 1, w_2_ = 1 | Not allowed |
| Model B | 5 | p_0_ =  0, p_1_ = 0, (p_2_ = 0.59733, p_3_ = 0.40267),  w_0_ =0.12205, w_1_ =1.18934, w_2_ = 0.04224 | **NEB:** None |

***Likelihood Ratio Tests for adam2:***

| **Comparison** | **Null Model lnL** | **Alt Model lnL** | ***df*** | **Adjusted deltaL** | **Critical Value** | **Significant?** |
| --- | --- | --- | --- | --- | --- | --- |
| ***Site Analysis*** |  |  |  |  |  |  |
| m0 v m3Discrtk2 | -15264.16288 | -14752.16817 | 2 | 1023.989412 | 5.99 | YES |
| m3Discrtk2 v m3Discrtk3 | -14752.16817 | -14678.25281 | - | 73.915361 | 1 | YES |
| m1Neutral v m2Selection | -14753.10507 | -14691.90877 | 2 | 122.392612 | 5.99 | YES |
| m7 v m8 | -14739.14385 | -14670.28361 | 2 | 137.72048 | 5.99 | YES |
| m8a v m8 | -14724.947 | -14670.28361 | 1 | 109.326792 | 2.71 | YES |
| ***Branch Analysis*** |  |  |  |  |  |  |
| ***Human :*** |  |  |  |  |  |  |
| ModelA v m1Neutral | -14752.9021 | -14753.10507 | 2 | 0.405958 | 5.99 | NO |
| ModelA v ModelAnull | -14752.9021 | -14753.105075 | 1 | 0.40596 | 3.84 | NO |
| ModelB v m3Discrtk2 | -14751.911362 | -14752.16817 | 2 | 0.513624 | 5.99 | NO |
| ***Chimp :*** |  |  |  |  |  |  |
| ModelA v m1Neutral | -14751.74232 | -14753.10507 | 2 | 2.725508 | 5.99 | NO |
| ModelA v ModelAnull | -14751.74232 | -14751.771550 | 1 | 0.05846 | 3.84 | NO |
| ModelB v m3Discrtk2 | -14750.726249 | -14752.16817 | 2 | 2.88385 | 5.99 | NO |
| ***Macaque :*** |  |  |  |  |  |  |
| ModelA v m1Neutral | -14751.49307 | -14753.10507 | 2 | 3.224012 | 5.99 | YES |
| ModelA v ModelAnull | -14751.49307 | -14751.550205 | 1 | 0.114274 | 3.84 | NO |
| ModelB v m3Discrtk2 | -14750.57921 | -14752.16817 | 2 | 3.17792 | 5.99 | NO |
| ***Mouse :*** |  |  |  |  |  |  |
| ModelA v m1Neutral | -14753.105074 | -14753.10507 | 2 | 0 | 5.99 | NO |
| ModelA v ModelAnull | -14753.105074 | -14753.105075 | 1 | 2E-06 | 3.84 | NO |
| ModelB v m3Discrtk2 | -14751.045525 | -14752.16817 | 2 | 2.245298 | 5.99 | NO |
| ***Primates :*** |  |  |  |  |  |  |
| ModelA v m1Neutral | -14753.105075 | -14753.10507 | 2 | 2E-06 | 5.99 | NO |
| ModelA v ModelAnull | -14753.105075 | -14753.105074 | 1 | 2E-06 | 3.84 | NO |
| ModelB v m3Discrtk2 | -14752.168174 | -14752.16817 | 2 | 0 | 5.99 | NO |
| ***Rodents :*** |  |  |  |  |  |  |
| ModelA v m1Neutral | -14753.10508 | -14753.10507 | 2 | 2E-06 | 5.99 | NO |
| ModelA v ModelAnull | -14753.10508 | -14753.105075 | 1 | 0 | 3.84 | NO |
| ModelB v m3Discrtk2 | -14735.53025 | -14752.16817 | 2 | 33.27585 | 5.99 | YES |

**(b) Complete set of estimates for Catsper1_Exon1 from codeml:**

| **Model** | **P** | **Estimates of parameters** | **Positively**  **selected sites** |
| --- | --- | --- | --- |
| M0 : one ratio | 1 | w = 0.9438 | **NEB:** None, **BEB:** None |
| **Site-specific:**    M1:Neutral | 2 | p_0_=0.23061 , w_0_<1 | Not allowed |
| M2:Selection | 4 | p_0_= 0.18411, p_1_= 0.65127  (p_2_=0.16462 ), w_0_<1, w_1_ = 1, w_2_ = 3.17328 | **NEB:** 40>0.50, 4>0.95 **BEB:** 46>0.50, 4>0.95 |
| M3:Discrete(K = 2) | 3 | p_0_= 0.39805 , (p_1_=0.60195 )  w_0_=0.24359, w_1_= 1.71676 | **NEB:** 0>0.50 |
| M3:Discrete(K = 3) | 5 | p_0_= 0.23088, p_1_= 0.67010, (p_2_=0.09902)  w_0_= 0.09268 , w_1_=1.17408, w_2_= 3.90174 | **NEB :** 209>0.50, 186>0.95, 130>0.99 |
| M7: Beta | 2 | p= 0.06046 , q=0.01243 | Not allowed |
| M8: Beta&Omega > 1 | 4 | p_0_=0.82736, p =0.13661, q = 0.03850  (p_1_= 0.17264), w = 3.13071 | **NEB:** 45>0.50, 4>0.95 **BEB** : 97>0.50 7>0.95, 1>0.99 |
| M8a: Beta&Omega = 1 | 3 | p_0_=0.23129  , p =7.73621 , q = 99.00000  (p_1_= 0.76871), w = 1 | Not allowed |
| **Branch-specific: Human** |  |  |  |
| Model A | 4 | p_0_ = 0, p_1_ = 0,  (p_2_ = 0.23266, p_3_ = 0.76734),  w_0_ = 0.06615, w_1_ = 1, w_2_ = 1.56616 | **NEB:** 363>0.50,464>0.95, 363>0.99 **BEB:** 4>0.50 |
| Model A null | 3 | p_0_ = 0, p_1_ =0,  (p_2_ = 0.23273 , p_3_ = 0.76727), w_0_ =  0.06621 , w_1_ = 1, w_2_ = 1 | Not allowed |
| Model B | 5 | p_0_ = 0, p_1_ =0, (p_2_ = 0.38629, p_3_ =0.61371 ),  w_0_ = 0.22474, w_1_ = 1.69600 , w_2_ = 1.61272 | **NEB :** 363>0.50, 63>0.95, 363>0.99 |
| **Branch-specific: Chimp** |  |  |  |
| Model A | 4 | p_0_ = 0.18928, p_1_ = 0.63150,  (p_2_ = 0.04133, p_3_ =  0.13789),  w_0_ =  0.07031, w_1_ = 1, w_2_ = 1.41789 | **NEB:** 0>0.50 **BEB**: 0>0.50 |
| Model A null | 3 | p_0_ = 0.18928, p_1_ = 0.63150,  (p_2_ = 0.04133 , p_3_ = 0.13789),  w_0_ =  0.07031, w_1_ = 1, w_2_ = 1 | Not allowed |
| Model B | 5 | p_0_ = 0.31752, p_1_ =  0.48018 , (p_2_ = 0.08052, p_3_ = 0.12177),  w_0_ = 0.24359, w_1_ = 1.71676, w_2_ = 1.20375 | **NEB:** 0>0.50 |
| **Branch-specific: Macaque** |  |  |  |
| Model A | 4 | p_0_ = 0.21476, p_1_ = 0.71515,  (p_2_ = 0.01619 , p_3_ = 0.05390),  w_0_ =   0.06853, w_1_ = 1, w_2_ = 5.27750 | **NEB:** 1>0.50  **BEB:** 1>0.50 |
| Model A null | 3 | p_0_ = 0.22902, p_1_ = 0.76401,  (p_2_ = 0.00161, p_3_ =  0.00536),  w_0_ =   0.07024 , w_1_ = 1, w_2_ = 1 | Not allowed |
| Model B | 5 | p_0_ = 0.39054 , p_1_ = 0.59232,  (p_2_ = 0.00681 , p_3_ = 0.01033),  w_0_ =  0.24184, w_1_ =1.71382, w_2_ = 5.16526 | **NEB:** 0>0.50 |
| **Branch-specific: Gorilla** |  |  |  |
| Model A | 4 | p_0_ = 0.23061, p_1_ =  0.76939,  (p_2_ = 0, p_3_ = 0), w_0_ =  0.07031,  w_1_ = 1, w_2_ = 1 | **NEB:** None **BEB:** None |
| Model A null | 3 | p_0_ = 0.23061, p_1_ =0.76939 ,  (p_2_ = 0, p_3_ = 0), w_0_ =  0.07031 ,  w_1_ = 1, w_2_ = 1 | Not allowed |
| Model B | 5 | p_0_ = 0.15064, p_1_ = 0.22748 ,  (p_2_ = 0.24775, p_3_ = 0.37413),  w_0_ =  0.24771, w_1_ =1.75003, w_2_ =0 | **NEB:** None |
| **Branch-specific: Cercopithecidae** |  |  |  |
| Model A | 4 | p_0_ = 0.23061, p_1_ = 0.76939 ,  (p_2_ = 0, p_3_ = 0), w_0_ =   0.07031  w_1_ = 1, w_2_ = 1 | **NEB:** None **BEB:** None |
| Model A null | 3 | p_0_ =  0.23061 , p_1_ = 0.76939,  (p_2_ = 0 p_3_ = 0), w_0_ =  0.07031,  w_1_ = 1, w_2_ = 1 | Not allowed |
| Model B | 5 | p_0_ =  0.30816, p_1_ = 0.46799,  (p_2_ = 0.08888, p_3_ = 0.13497),  w_0_ = 0.24692, w_1_ =  1.74843, w_2_ = 0 | **NEB:** None |
| **Branch-specific: Hominidae** |  |  |  |
| Model A | 4 | p_0_ =  0.18623, p_1_ = 0.61846,  (p_2_ = 0.04520 , p_3_ = 0.15011), w_0_ = 0.06844, w_1_ = 1, w_2_ = 1 | **NEB:** None **BEB:** None |
| Model A null | 3 | p_0_ =  0.18623, p_1_ =  0.61846,  (p_2_ = 0.04520, p_3_ = 0.15011),  w_0_ =  0.06844, w_1_ = 1, w_2_ = 1 | Not allowed |
| Model B | 5 | p_0_ = 0.08972, p_1_ = 0.13965,  (p_2_ = 0.30143, p_3_ =  0.46920),  w_0_ =  0.24130 , w_1_ = 1.78475, w_2_ =  0.12024 | **NEB:** None |

***Likelihood Ratio Tests for Catsper1_Exon1:***

| **Comparison** | **Null Model lnL** | **Alt Model lnL** | ***df*** | **Adjusted deltaL** | **Critical Value** | **Significant?** |
| --- | --- | --- | --- | --- | --- | --- |
| ***Site Analysis*** |  |  |  |  |  |  |
| m0 v m3Discrtk2 | -5308.477759 | -5257.816479 | 2 | 101.32256 | 5.99 | YES |
| m3Discrtk2 v m3Discrtk3 | -5257.816479 | -5253.104026 | - | 4.712453 | 1 | YES |
| m1Neutral v m2Selection | -5267.435019 | -5253.234859 | 2 | 28.40032 | 5.99 | YES |
| m7 v m8 | -5267.801857 | -5253.289195 | 2 | 29.025324 | 5.99 | YES |
| m8a v m8 | -5267.447975 | -5253.289195 | 1 | 28.31756 | 2.71 | YES |
| ***Branch Analysis*** |  |  |  |  |  |  |
| ***Human*** |  |  |  |  |  |  |
| ModelA v m1Neutral | -5266.312655 | -5267.435019 | 2 | 2.244728 | 5.99 | NO |
| ModelA v ModelAnull | -5266.312655 | -5266.399601 | 1 | 0.173892 | 3.84 | NO |
| ModelB v m3Discrtk2 | -5256.816869 | -5257.816479 | 2 | 1.99922 | 5.99 | NO |
| ***Chimp*** |  |  |  |  |  |  |
| ModelA v m1Neutral | -5267.435029 | -5267.435019 | 2 | 2E-05 | 5.99 | NO |
| ModelA v ModelAnull | -5267.435029 | -5267.435027 | 1 | 4E-06 | 3.84 | NO |
| ModelB v m3Discrtk2 | -5257.816492 | -5257.816479 | 2 | 2.6E-05 | 5.99 | NO |
| ***Macaque*** |  |  |  |  |  |  |
| ModelA v m1Neutral | -5267.252488 | -5267.435019 | 2 | 0.365062 | 5.99 | NO |
| ModelA v ModelAnull | -5267.252488 | -5267.4349 | 1 | 0.364824 | 3.84 | NO |
| ModelB v m3Discrtk2 | -5257.80282 | -5257.816479 | 2 | 0.027318 | 5.99 | NO |
| ***Gorilla*** |  |  |  |  |  |  |
| ModelA v m1Neutral | -5267.435019 | -5267.435019 | 2 | 0 | 5.99 | NO |
| ModelA v ModelAnull | -5267.435019 | -5267.435019 | 1 | 0 | 3.84 | NO |
| ModelB v m3Discrtk2 | -5256.669264 | -5257.816479 | 2 | 2.29443 | 5.99 | NO |
| ***Hominidae*** |  |  |  |  |  |  |
| ModelA v m1Neutral | -5267.334989 | -5267.435019 | 2 | 0.20006 | 5.99 | NO |
| ModelA v ModelAnull | -5267.334989 | -5267.334989 | 1 | 0 | 3.84 | NO |
| ModelB v m3Discrtk2 | -5255.813942 | -5257.816479 | 2 | 4.005074 | 5.99 | NO |
| ***Cercopithecidae*** |  |  |  |  |  |  |
| ModelA v m1Neutral | -5267.435019 | -5267.435019 | 2 | 0 | 5.99 | NO |
| ModelA v ModelAnull | -5267.435019 | -5267.435019 | 1 | 0 | 3.84 | NO |
| ModelB v m3Discrtk2 | -5257.55949 | -5257.816479 | 2 | 0.513978 | 5.99 | NO |

**(c) Complete set of estimates for Catsper_Mammals from codeml:**

| **Model** | **P** | **Estimates of parameters** | **Positively**  **selected sites** |
| --- | --- | --- | --- |
| M0 : one ratio | 1 | w = 0.4109 | **NEB:** None **BEB:** None |
| **Site-specific:**    M1:Neutral | 2 | p_0_=0.49925, w_0_<1 | Not allowed |
| M2:Selection | 4 | p_0_= 0.45323, p_1_= 0.42564  (p_2_=0.12113), w_0_<1, w_1_ = 1, w_2_ =  4.93971 | **NEB:** 81>0.50, 18>0.95, 7>0.99 **BEB:** 85>0.50, 18>0.95 7>0.99 |
| M3:Discrete(K = 2) | 3 | p_0_=0.54823, (p_1_= 0.45177)  w_0_=0.12061, w_1_= 1.37162 | **NEB**: 0>0.50 |
| M3:Discrete(K = 3) | 5 | p_0_= 0.35362, p_1_= 0.40982, (p_2_= 0.23656)  w_0_=0.03795, w_1_=0.54802, w_2_=2.56196 | **NEB:** 180>0.50, 63>0.95, 32>0.99 |
| M7: Beta | 2 | p=0.28339 , q= 0.31193 | Not allowed |
| M8: Beta&Omega > 1 | 4 | p_0_= 0.83315, p =0.34233, q =  0.51278  (p_1_=0.16685), w =3.26879 | **NEB:** 128>0.50, 27>0.95, 7>0.99 **BEB:** 124>0.50, 30>0.95, 8>0.99 |
| M8a: Beta&Omega = 1 | 3 | p_0_=0.59360, p = 0.60936, q =  3.50222  (p_1_=0.40640), w = 1 | Not allowed |
| **Branch-specific:Ferungulata** |  |  |  |
| Model A | 4 | p_0_ =  0.48740, p_1_ =0.46800 ,  (p_2_ = 0.02276, p_3_ = 0.02185), w_0_ =  0.08841 ,  w_1_ = 1, w_2_ =  998.99981 | **NEB:** 13>0.50, 1>0.95 **BEB** : 5>0.50, 1>0.95 |
| Model A null | 3 | p_0_ =  0.49037, p_1_ = 0.49065,  (p_2_ = 0.00948, p_3_ = 0.00949),  w_0_ =   0.08503, w_1_ = 1, w_2_ = 1 | Not allowed |
| Model B | 5 | p_0_ = 0.51692 , p_1_ = 0.42612,  (p_2_ =0.03122, p_3_ = 0.02574),  w_0_ =  0.12085, w_1_ = 1.37445, w_2_ = 0 | **NEB** : None |
| **Branch-specific: Mouse** |  |  |  |
| Model A | 4 | p_0_ =  0.45052 , p_1_ = 0.44724,  (p_2_ = 0.05131, p_3_ =  0.05093),  w_0_ = 0.08353 , w_1_ = 1, w_2_ = 1 | **NEB:** None **BEB:** None |
| Model A null | 3 | p_0_ = 0.45052, p_1_ =  0.44724,  (p_2_ = 0.05131, p_3_ = 0.05093),  w_0_ =  0.08353 , w_1_ = 1, w_2_ = 1 | Not allowed |
| Model B | 5 | p_0_ = 0.23435, p_1_ = 0.18155,  (p_2_ = 0.32913, p_3_ = 0.25497),  w_0_ = 0.12693, w_1_ = 1.50506, w_2_ = 0.24614 | **NEB:** None |
| **Branch-specific: Rodents** |  |  |  |
| Model A | 4 | p_0_ =  0.47315, p_1_ = 0.47238,  (p_2_ = 0.02726, p_3_ =0.02721), w_0_ =  0.08373, w_1_ = 1, w_2_ = 999 | **NEB:** 16>0.50  **BEB:** 11>0.50 |
| Model A null | 3 | p_0_ = 0.46496 , p_1_ = 0.46638,  (p_2_ = 0.03428, p_3_ =  0.03438),  w_0_ =  0.08380, w_1_ = 1, w_2_ = 1 | Not allowed |
| Model B | 5 | p_0_ = 0.52986, p_1_ = 0.42545 ,  (p_2_ = 0.02478, p_3_ = 0.01990),  w_0_ =  0.12088, w_1_ =  1.38175, w_2_ = 999 | **NEB:** 12>0.50, 1>0.95 |

***Likelihood Ratio Tests for Catsper1_Mammals:***

| **Comparison** | **Null Model lnL** | **Alt Model lnL** | ***df*** | **Adjusted deltaL** | **Critical Value** | **Significant?** |
| --- | --- | --- | --- | --- | --- | --- |
| ***Site Analysis*** |  |  |  |  |  |  |
| m0 v m3Discrtk2 | -14318.57354 | -13774.17103 | 2 | 1088.80501 | 5.99 | YES |
| m3Discrtk2 v m3Discrtk3 | -13774.17103 | -13707.34856 | - | 66.822474 | 1 | YES |
| m1Neutral v m2Selection | -13783.62098 | -13720.79124 | 2 | 125.65948 | 5.99 | YES |
| m7 v m8 | -13774.76681 | -13703.95027 | 2 | 141.63307 | 5.99 | YES |
| m8a v m8 | -13760.31581 | -13703.95027 | 1 | 112.73108 | 2.71 | YES |
| ***Branch Analysis*** |  |  |  |  |  |  |
| ***Ferungulata*** |  |  |  |  |  |  |
| ModelA v m1Neutral | -13777.32823 | -13783.62098 | 2 | 12.585508 | 5.99 | YES |
| ModelA v ModelAnull | -13777.32823 | -13783.508699 | 1 | 12.360948 | 3.84 | YES |
| ModelB v m3Discrtk2 | -13774.16148 | -13774.17103 | 2 | 0.01911 | 5.99 | NO |
| ***Mouse*** |  |  |  |  |  |  |
| ModelA v m1Neutral | -13782.29312 | -13783.62098 | 2 | 2.655726 | 5.99 | NO |
| ModelA v ModelAnull | -13782.29312 | -13782.293116 | 1 | 0 | 3.84 | NO |
| ModelB v m3Discrtk2 | -13769.41208 | -13774.17103 | 2 | 9.51791 | 5.99 | YES |
| ***Rodents*** |  |  |  |  |  |  |
| ModelA v m1Neutral | -13774.79204 | -13783.62098 | 2 | 17.657886 | 5.99 | YES |
| ModelA v ModelAnull | -13774.79204 | -13783.349416 | 1 | 17.11476 | 3.84 | YES |
| ModelB v m3Discrtk2 | -13765.68047 | -13774.17103 | 2 | 16.981128 | 5.99 | YES |

**(d)** **Complete set of estimates for Col1a1 from codeml:**

| **Model** | **P** | **Estimates of parameters** | **Positively**  **selected sites** |
| --- | --- | --- | --- |
| M0 : one ratio | 1 | w = 0.1416 | **NEB:** None **BEB:** None |
| **Site-specific:**  M1:Neutral | 2 | p_0_= 0.85392 , w_0_<1 | Not allowed |
| M2:Selection | 4 | p_0_= 0.85767, p_1_= 0.12688  (p_2_=0.01544), w_0_<1, w_1_ = 1, w_2_ = 4.61965 | **NEB:** 18>0.50, 6>0.95, 1>0.99 **BEB:** 55>0.50, 7>0.95, 1>0.99 |
| M3:Discrete(K = 2) | 3 | p_0_=  0.88124, (p_1_= 0.11876 )  w_0_= 0.02425, w_1_=1.42696 | **NEB:** 98>0.50, 103>0.95, 66>0.99 |
| M3:Discrete(K = 3) | 5 | p_0_=  0.83159 , p_1_= 0.14630, (p_2_=0.02211)  w_0_= 0.01085 , w_1_=0.76692, w_2_= 3.91072 | **NEB:** 28>0.50, 6>0.95, 3>0.99 |
| M7: Beta | 2 | p= 0.02991, q=0.18206 | Not allowed |
| M8: Beta&Omega > 1 | 4 | p_0_= 0.98023, p = 0.04796, q =  0.32286  (p_1_=0.01977), w =4.09285 | **NEB:** 25>0.50, 6>0.95, 3>0.99 **BEB**: 66>0.50, 21>0.95, 8>0.99 |
| M8a: Beta&Omega = 1 | 3 | p_0_=0.85466 , p = 1.09499, q =  65.75907  (p_1_= 0.14534), w = 1 | Not allowed |
| **Branch-specific: Human** |  |  |  |
| Model A | 4 | p_0_ = 0.81042 , p_1_ = 0.13884 ,  (p_2_ = 0.04332, p_3_ = 0.00742), w_0_ =  0.01504, w_1_ = 1, w_2_ = 1 | **NEB:** None **BEB:** None |
| Model A null | 3 | p_0_ = 0.81042, p_1_ = 0.13884,  (p_2_ = 0.04331, p_3_ = 0.00742),   w_0_ =   0.01504  , w_1_ = 1, w_2_ = 1 | Not allowed |
| Model B | 5 | p_0_ = 0, p_1_ = 0, (p_2_ = 0.88103, p_3_ = 0.11897),  w_0_ = 0.02393, w_1_ = 1.43904, w_2_ = 0.05321 | **NEB:** None |
| **Branch-specific: Chimp** |  |  |  |
| Model A | 4 | p_0_ =  0.85392, p_1_ = 0.14608,  (p_2_ = 0, p_3_ = 0), w_0_ =  0.01534, w_1_ = 1, w_2_ = 1 | **NEB:** None **BEB:** None |
| Model A null | 3 | p_0_ = 0.85392, p_1_ =  0.14608 (p_2_ = 0, p_3_ = 0), w_0_ =  0.01534, w_1_ = 1, w_2_ = 1 | Not allowed |
| Model B | 5 | p_0_ = 0, p_1_ = 0 (p_2_ = 0.88141, p_3_ = 0.11859),  w_0_ =  0.02450, w_1_ = 1.44502, w_2_ = 0 | **NEB:** None |
| **Branch-specific: Primates** |  |  |  |
| Model A | 4 | p_0_ =  0.85392, p_1_ =  0.14608 ,  (p_2_ = 0, p_3_ = 0), w_0_ =   0.01534 , w_1_ = 1, w_2_ = 1 | **NEB:** None **BEB:** None |
| Model A null | 3 | p_0_ = 0.85392, p_1_ = 0.14608,  (p_2_ = 0, p_3_ = 0), w_0_ =  0.01534, w_1_ = 1, w_2_ = 1 | Not allowed |
| Model B | 5 | p_0_ = 0.73216  , p_1_ = 0.09862 ,  (p_2_ =0.14914 , p_3_ = 0.02009),  w_0_ = 0.02445, w_1_ = 1.44146, w_2_ = 0 | **NEB:** None |
| **Branch-specific: Mouse** |  |  |  |
| Model A | 4 | p_0_ =  0.81967, p_1_ = 0.14052,  (p_2_ = 0.03398 , p_3_ = 0.00583),  w_0_ =  0.01369 , w_1_ = 1, w_2_ = 1 | **NEB:** None **BEB:** None |
| Model A null | 3 | p_0_ =0.81968  , p_1_ = 0.14052,  (p_2_ = 0.03398  , p_3_ =0.00583),  w_0_ =0.01369      , w_1_ = 1, w_2_ = 1 | Not allowed |
| Model B | 5 | p_0_ = 0.57848, p_1_ = 0.07820,  (p_2_ = 0.30244, p_3_ =  0.04088),  w_0_ =  0.02242, w_1_ = 1.45166, w_2_ = 0.13101 | **NEB:** None |
| **Branch-specific: Rodents** |  |  |  |
| Model A | 4 | p_0_ = 0.84028, p_1_ = 0.13798,  (p_2_ =  0.01867, p_3_ = 0.00307), w_0_ =  0.01325 , w_1_ = 1, w_2_ = 72.73297 | **NEB:** 22>0.50, 1>0.95, **BEB:** 19>0.50, 1>0.95 |
| Model A null | 3 | p_0_ =  0.81681 , p_1_ = 0.13522,  (p_2_ = 0.04116, p_3_ = 0.00681),  w_0_ =   0.01181, w_1_ = 1, w_2_ = 1 | Not allowed |
| Model B | 5 | p_0_ = 0.86550 , p_1_ = 0.11522 ,  (p_2_ = 0.01701, p_3_ = 0.00227),  w_0_ = 0.02083, w_1_ = 1.34868, w_2_ =72.76640 | **NEB:** 18>0.50, 1>0.95 |
| **Branch-specific: Glires** |  |  |  |
| Model A | 4 | p_0_ = 0.85246, p_1_ = 0.14301,  (p_2_ = 0.00388, p_3_ = 0.00065), w_0_ =   0.01514, w_1_ = 1, w_2_ = 106.93835 | **NEB:** 6>0.50  **BEB**: 4>0.50 |
| Model A null | 3 | p_0_ =  0.81333, p_1_ = 0.13800 , (p_2_ = 0.04161 , p_3_ = 0.00706), w_0_ =  0.01495, w_1_ = 1, w_2_ = 1 | Not allowed |
| Model B | 5 | p_0_ = 0.87908, p_1_ = 0.11577,  (p_2_ = 0.00455, p_3_ = 0.00060),  w_0_ =  0.02391, w_1_ = 1.43229, w_2_ = 77.02610 | **NEB:** 6>0.50 |

***Likelihood Ratio Tests for Col1a1:***

| **Comparison** | **Null Model lnL** | **Alt Model lnL** | ***df*** | **Adjusted deltaL** | **Critical Value** | **Significant?** |
| --- | --- | --- | --- | --- | --- | --- |
| ***Site Analysis*** |  |  |  |  |  |  |
| m0 v m3Discrtk2 | -14262.14652 | -13910.65753 | 2 | 702.977978 | 5.99 | YES |
| m3Discrtk2 v m3Discrtk3 | -13910.65753 | -13895.56993 | - | 15.087598 | 1 | YES |
| m1Neutral v m2Selection | -13918.46452 | -13896.43221 | 2 | 44.064616 | 5.99 | YES |
| m7 v m8 | -13923.25517 | -13895.60715 | 2 | 55.296052 | 5.99 | YES |
| m8a v m8 | -13918.48142 | -13895.60715 | 1 | 45.74854 | 2.71 | YES |
| ***Branch Analysis*** |  |  |  |  |  |  |
| ***Human*** |  |  |  |  |  |  |
| ModelA v m1Neutral | -13918.09113 | -13918.46452 | 2 | 0.746788 | 5.99 | NO |
| ModelA v ModelAnull | -13918.09113 | -13918.091126 | 1 | 0 | 3.84 | NO |
| ModelB v m3Discrtk2 | -13907.9124 | -13910.65753 | 2 | 5.490264 | 5.99 | NO |
| ***Chimp*** |  |  |  |  |  |  |
| ModelA v m1Neutral | -13918.46452 | -13918.46452 | 2 | 4E-06 | 5.99 | NO |
| ModelA v ModelAnull | -13918.46452 | -13918.464527 | 1 | 1E-05 | 3.84 | NO |
| ModelB v m3Discrtk2 | -13907.0651 | -13910.65753 | 2 | 7.184856 | 5.99 | YES |
| ***Primates*** |  |  |  |  |  |  |
| ModelA v m1Neutral | -13918.46453 | -13918.46452 | 2 | 1.4E-05 | 5.99 | NO |
| ModelA v ModelAnull | -13918.46453 | -13918.464520 | 1 | 1.4E-05 | 3.84 | NO |
| ModelB v m3Discrtk2 | -13910.46919 | -13910.65753 | 2 | 0.376672 | 5.99 | NO |
| ***Mouse*** |  |  |  |  |  |  |
| ModelA v m1Neutral | -13917.11309 | -13918.46452 | 2 | 2.702866 | 5.99 | NO |
| ModelA v ModelAnull | -13917.11309 | -13917.113087 | 1 | 0 | 3.84 | NO |
| ModelB v m3Discrtk2 | -13908.74546 | -13910.65753 | 2 | 3.824134 | 5.99 | NO |
| ***Rodents*** |  |  |  |  |  |  |
| ModelA v m1Neutral | -13902.08547 | -13918.46452 | 2 | 32.758092 | 5.99 | YES |
| ModelA v ModelAnull | -13902.08547 | -13912.86005 | 1 | 21.54916 | 3.84 | YES |
| ModelB v m3Discrtk2 | -13897.52977 | -13910.65753 | 2 | 26.255526 | 5.99 | YES |
| ***Gleries*** |  |  |  |  |  |  |
| ModelA v m1Neutral | -13916.25375 | -13918.46452 | 2 | 4.421542 | 5.99 | NO |
| ModelA v ModelAnull | -13916.25375 | -13917.746645 | 1 | 2.985792 | 3.84 | NO |
| ModelB v m3Discrtk2 | -13908.48629 | -13910.65753 | 2 | 4.342474 | 5.99 | NO |

**(e)** **Complete set of estimates for Ph20 from codeml:**

| **Model** | **P** | **Estimates of parameters** | **Positively**  **selected sites** |
| --- | --- | --- | --- |
| M0 : one ratio | 1 | w = 0.4409 | **NEB:** None **BEB:** None |
| **Site-specific:**    M1:Neutral | 2 | p_0_= 0.52978, w_0_<1 | Not allowed |
| M2:Selection | 4 | p_0_= 0.51704, p_1_= 0.45258  (p_2_= 0.03039), w_0_<1, w_1_ = 1, w_2_ = 6.32123 | **NEB:** 10>0.50, 3>0.95, 1>0.99 **BEB:** 10>0.50, 3>0.95 1>0.99 |
| M3:Discrete(K = 2) | 3 | p_0_=  0.54429, (p_1_= 0.45571)  w_0_=0.14194, w_1_=1.08180 | **NEB:** 233>0.50, 144>0.95, 102>0.99 |
| M3:Discrete(K = 3) | 5 | p_0_= 0.43915, p_1_=0.43535 , (p_2_= 0.12550)  w_0_= 0.09890 , w_1_=0.70405, w_2_= 2.25887 | **NEB** : 57>0.50, 9>0.05, 2>0.99 |
| M7: Beta | 2 | p= 0.42610 , q=0.47032 | Not allowed |
| M8: Beta&Omega > 1 | 4 | p_0_=0.87658, p =0.56141, q =0.83349  (p_1_= 0.12342), w =2.20500 | **NEB:** 56>0.50, 4>0.95 **BEB:** 39>0.50, 3>0.95 |
| M8a: Beta&Omega = 1 | 3 | p_0_=  0.60502, p =1.16966, q = 5.24454  (p_1_=0.39498), w = 1 | Not allowed |
| **Branch-specific: Human** |  |  |  |
| Model A | 4 | p_0_ =  0.29830, p_1_ = 0.26626,  (p_2_ = 0.23007, p_3_ = 0.20536), w_0_ =  0.12976 , w_1_ = 1, w_2_ = 1.20759 | **NEB:** 1>0.50 **BEB:** 1>0.50 |
| Model A null | 3 | p_0_ = 0.26060, p_1_ = 0.23265,  (p_2_ = 0.26773 , p_3_ = 0.23902),  w_0_ =   0.12974 , w_1_ = 1, w_2_ = 1 | Not allowed |
| Model B | 5 | p_0_ = 0.31095, p_1_ =  0.26252,  (p_2_ =0.23127 , p_3_ = 0.19525),  w_0_ =0.14031, w_1_ = 1.07815, w_2_ = 1.18613 | **NEB:** 1>0.50 |
| **Branch-specific: Chimp** |  |  |  |
| Model A | 4 | p_0_ = 0.52978, p_1_ = 0.47022,  (p_2_ = 0, p_3_ = 0), w_0_ =  0.13089 , w_1_ = 1,  w_2_ = 1 | **NEB:** None **BEB:** None |
| Model A null | 3 | p_0_ =  0.52978 , p_1_ = 0.47022,  (p_2_ =0, p_3_ = 0),  w_0_ =  0.13089 , w_1_ = 1, w_2_ = 1 | Not allowed |
| Model B | 5 | p_0_ = 0.54429, p_1_ = 0.45571, (p_2_ = 0, p_3_ = 0),  w_0_ =  0.14194 , w_1_ = 1.08180, w_2_ = 0 | **NEB:** None |
| **Branch-specific: Macaque** |  |  |  |
| Model A | 4 | p_0_ = 0.52692, p_1_ = 0.46582 ,  (p_2_ = 0.00385  , p_3_ = 0.00340),  w_0_ =   0.13004, w_1_ = 1, w_2_ = 39.75311 | **NEB:** 2>0.50  **BEB:** 1>0.50 |
| Model A null | 3 | p_0_ = 0.50516 , p_1_ = 0.44657,  (p_2_ =  0.02562, p_3_ = 0.02265),  w_0_ =   0.13023 , w_1_ = 1, w_2_ = 1 | Not allowed |
| Model B | 5 | p_0_ = 0.46754, p_1_ = 0.39145,  (p_2_ = 0.07675, p_3_ = 0.06426),  w_0_ =0.14263, w_1_ = 1.08843, w_2_ = 0 | **NEB:** None |
| **Branch-specific: Primates** |  |  |  |
| Model A | 4 | p_0_ = 0.52978, p_1_ = 0.47022 ,  (p_2_ = 0, p_3_ = 0),  w_0_ =  0.13089, w_1_ = 1, w_2_ = 1 | **NEB:** None **BEB:** None |
| Model A null | 3 | p_0_ = 0.52978, p_1_ = 0.47022,  (p_2_ = 0, p_3_ = 0),  w_0_ =  0.13089 w_1_ = 1, w_2_ = 1 | Not allowed |
| Model B | 5 | p_0_ = 0.51983, p_1_ = 0.43545 ,  (p_2_ = 0.02434 , p_3_ =  0.02039),  w_0_ =  0.14235 , w_1_ = 1.08587, w_2_ = 0 | **NEB:** None |
| **Branch-specific: GuineaPig** |  |  |  |
| Model A | 4 | p_0_ = 0.50741, p_1_ = 0.42958 ,  (p_2_ = 0.03412, p_3_ = 0.02888),  w_0_ =  0.12708, w_1_ = 1, w_2_ = 11.47503 | **NEB:** 16>0.50, 3>0.95, 2>0.99 **BEB:** 13>0.50 3>0.95, 1>0.99 |
| Model A null | 3 | p_0_ = 0.44588, p_1_ = 0.38045 ,  (p_2_ = 0.09371, p_3_ =  0.07996),  w_0_ =   0.12080, w_1_ = 1, w_2_ = 1 | Not allowed |
| Model B | 5 | p_0_ = 0.52497 , p_1_ = 0.41362 ,  (p_2_ = 0.03435  , p_3_ = 0.02706),  w_0_ =  0.14010, w_1_ = 1.09953, w_2_ = 12.56461 | **NEB:** 16>0.50, 3>0.95, 2>0.99 |
| **Branch-specific: Glires** |  |  |  |
| Model A | 4 | p_0_ = 0.49833, p_1_ = 0.44076,  (p_2_ = 0.03232 , p_3_ =  0.02859),  w_0_ =    0.12994, w_1_ = 1, w_2_ = 3.95170 | **NEB:** 1>0.50 **BEB** 1>0.50 |
| Model A null | 3 | p_0_ = 0.46298, p_1_ =  0.41043,  (p_2_ = 0.06711, p_3_ =  0.05949),  w_0_ = 0.13002, w_1_ = 1, w_2_ = 1 | Not allowed |
| Model B | 5 | p_0_ =  0.50949, p_1_ = 0.42529 ,  (p_2_ = 0.03555, p_3_ = 0.02967),  w_0_ = 0.14085, w_1_ =1.08107 , w_2_ = 3.54385 | **NEB:** 1>0.50 |

***Likelihood Ratio Tests for Ph-20:***

| **Comparison** | **Null Model lnL** | **Alt Model lnL** | ***df*** | **Adjusted deltaL** | **Critical Value** | **Significant?** |
| --- | --- | --- | --- | --- | --- | --- |
| ***Site Analysis*** |  |  |  |  |  |  |
| m0 v m3Discrtk2 | -10118.56222 | -9888.813777 | 2 | 459.496888 | 5.99 | YES |
| m3Discrtk2 v m3Discrtk3 | -9888.813777 | -9873.203363 | - | 15.610414 | 1 | YES |
| m1Neutral v m2Selection | -9889.378832 | -9875.395012 | 2 | 27.96764 | 5.99 | YES |
| m7 v m8 | -9894.867839 | -9873.241137 | 2 | 43.253404 | 5.99 | YES |
| m8a v m8 | -9884.334003 | -9873.241137 | 1 | 22.185732 | 2.71 | YES |
| ***Branch Analysis*** |  |  |  |  |  |  |
| ***Human*** |  |  |  |  |  |  |
| ModelA v m1Neutral | -9888.811109 | -9889.378832 | 2 | 1.135446 | 5.99 | NO |
| ModelA v ModelAnull | -9888.811109 | -9888.813275 | 1 | -0.004332 | 3.84 | NO |
| ModelB v m3Discrtk2 | -9888.293271 | -9888.813777 | 2 | 1.041012 | 5.99 | NO |
| ***Chimp*** |  |  |  |  |  |  |
| ModelA v m1Neutral | -9889.378832 | -9889.378832 | 2 | 0 | 5.99 | NO |
| ModelA v ModelAnull | -9889.378832 | -9889.378832 | 1 | 0 | 3.84 | NO |
| ModelB v m3Discrtk2 | -9888.813777 | -9888.813777 | 2 | 0 | 5.99 | NO |
| ***Macaque*** |  |  |  |  |  |  |
| ModelA v m1Neutral | -9887.391663 | -9889.378832 | 2 | 3.974338 | 5.99 | NO |
| ModelA v ModelAnull | -9887.391663 | -9889.162813 | 1 | 3.5423 | 3.84 | NO |
| ModelB v m3Discrtk2 | -9888.672405 | -9888.813777 | 2 | 0.282744 | 5.99 | NO |
| ***Primates*** |  |  |  |  |  |  |
| ModelA v m1Neutral | -9889.378832 | -9889.378832 | 2 | 0 | 5.99 | NO |
| ModelA v ModelAnull | -9889.378832 | -9889.378832 | 1 | 0.049672 | 3.84 | NO |
| ModelB v m3Discrtk2 | -9888.788941 | -9888.813777 | 2 | 0 | 5.99 | NO |
| ***GuineaPig*** |  |  |  |  |  |  |
| ModelA v m1Neutral | -9874.173396 | -9889.378832 | 2 | 30.410872 | 5.99 | YES |
| ModelA v ModelAnull | -9874.173396 | -9881.983656 | 1 | 15.62052 | 3.84 | YES |
| ModelB v m3Discrtk2 | -9873.408532 | -9888.813777 | 2 | 30.81049 | 5.99 | YES |
| ***Gleries*** |  |  |  |  |  |  |
| ModelA v m1Neutral | -9889.153276 | -9889.378832 | 2 | 0.451112 | 5.99 | NO |
| ModelA v ModelAnull | -9889.153276 | -9889.249378 | 1 | 0.192204 | 3.84 | NO |
| ModelB v m3Discrtk2 | -9888.602245 | -9888.813777 | 2 | 0.423064 | 5.99 | NO |

**(f)** **Complete set of estimates for Porimin from codeml:**

| **Model** | **P** | **Estimates of parameters** | **Positively**  **selected sites** |
| --- | --- | --- | --- |
| M0 : one ratio | 1 | w = 0.6134 | **NEB:** None **BEB:** None |
| **Site-specific:**  M1:Neutral | 2 | p_0_= 0.42486 , w_0_<1 | Not allowed |
| M2:Selection | 4 | p_0_= 0.37006, p_1_= 0.48647  (p_2_= 0.14347), w_0_<1, w_1_ = 1, w_2_ =  13.43331 | **NEB:** 27>0.50, 11>0.95, 4>0.99 **BEB:** 27>0.50, 11>0.95, 4>0.99 |
| M3:Discrete(K = 2) | 3 | p_0_=  0.56656, (p_1_= 0.43344)  w_0_= 0.23415, w_1_= 1.81265 | **NEB:** 84>0.50, 50>0.95, 29>0.99 |
| M3:Discrete(K = 3) | 5 | p_0_=0.33512, p_1_= 0.51600, (p_2_= 0.14888 )  w_0_= 0.10238, w_1_=0.88313, w_2_= 12.54783 | **NEB:** 27>0.50, 11>0.95, 5>0.99 |
| M7: Beta | 2 | p=0.35409, q= 0.23712 | Not allowed |
| M8: Beta&Omega > 1 | 4 | p_0_= 0.85067, p = 0.41864, q =  0.3295  (p_1_= 0.14933), w =12.21841 | **NEB:** 27>0.50, 11>0.95, 5>0.99 **BEB:** 30>0.50, 13>0.95, 5>0.99 |
| M8a: Beta&Omega = 1 | 3 | p_0_= 0.46679 , p = 1.26832, q =6.36786  (p_1_= 0.53321), w = 1 | Not allowed |
| **Branch-specific: Human** |  |  |  |
| Model A | 4 | p_0_ = 0.42486 , p_1_ = 0.57514,  (p_2_ = 0, p_3_ = 0), w_0_ =    0.12495, w_1_ = 1, w_2_ = 1 | **NEB:** None **BEB:** None |
| Model A null | 3 | p_0_ =  0.42486, p_1_ = 0.57514 ,  (p_2_ = 0, p_3_ = 0), w_0_ =  0.12495, w_1_ = 1, w_2_ = 1 | Not allowed |
| Model B | 5 | p_0_ =  0.16009 , p_1_ = 0.12082,  (p_2_ = 0.40981, p_3_ = 0.30928),  w_0_ = 0.23916, w_1_ = 1.86372 , w_2_ = 0 | **NEB:** None |
| **Branch-specific: Chimp** |  |  |  |
| Model A | 4 | p_0_ = 0.42486, p_1_ = 0.57514,  (p_2_ = 0, p_3_ = 0), w_0_ =   0.12495, w_1_ = 1, w_2_ = 1 | **NEB:** None **BEB:** None |
| Model A null | 3 | p_0_ = 0.42486, p_1_ = 0.57514,  (p_2_ = 0, p_3_ = 0), w_0_ =  0.12495, w_1_ = 1, w_2_ = 1 | Not allowed |
| Model B | 5 | p_0_ = 0.09110, p_1_ = 0.06866,  (p_2_ = 0.47913 , p_3_ = 0.36111),  w_0_ = 0.23986, w_1_ = 1.87172 , w_2_ = 0 | **NEB:** None |
| **Branch-specific: Macaque** |  |  |  |
| Model A | 4 | p_0_ =  0.42486, p_1_ = 0.57514,  (p_2_ = 0, p_3_ = 0), w_0_ =  0.12495, w_1_ = 1, w_2_ = 1 | **NEB:** None **BEB:** None |
| Model A null | 3 | p_0_ = 0.42486, p_1_ = 0.57514 (p_2_ = 0, p_3_ = 0),  w_0_ = 0.12495, w_1_ = 1, w_2_ = 1 | Not allowed |
| Model B | 5 | p_0_ = 0.39230, p_1_ = 0.29515 ,  (p_2_ = 0.17836, p_3_ = 0.13419),  w_0_ = 0.24000, w_1_ = 1.87154 , w_2_ = 0 | **NEB:** None |
| **Branch-specific: Primate** |  |  |  |
| Model A | 4 | p_0_ = 0.42486 , p_1_ = 0.57514,  (p_2_ = 0, p_3_ = 0), w_0_ =    0.12495, w_1_ = 1, w_2_ = 1 | **NEB:** None **BEB:** None |
| Model A null | 3 | p_0_ = 0.42486, p_1_ = 0.57514,  (p_2_ = 0, p_3_ = 0), w_0_ =   0.12495, w_1_ = 1, w_2_ = 1 | Not allowed |
| Model B | 5 | p_0_ = 0.54026, p_1_ = 0.41384,  (p_2_ =  0.02599 , p_3_ = 0.01991),  w_0_ = 0.23535, w_1_ = 1.82481, w_2_ = 0 | **NEB:** None |
| **Branch-specific: Rodents** |  |  |  |
| Model A | 4 | p_0_ =  0.37722, p_1_ = 0.46360,  (p_2_ = 0.07141 , p_3_ = 0.08777), w_0_ =  0.12103 , w_1_ = 1, w_2_ =  1.62302 | **NEB:** 7>0.50  **BEB:**3>0.50 |
| Model A null | 3 | p_0_ = 0.35014 , p_1_ = 0.43358,  (p_2_ = 0.09663, p_3_ = 0.11965),  w_0_ =   0.11989, w_1_ = 1, w_2_ = 1 | Not allowed |
| Model B | 5 | p_0_ = 0.11762, p_1_ = 0.07504,  (p_2_ = 0.49288 , p_3_ = 0.31446),  w_0_ =  0.24682, w_1_ = 2.38910 , w_2_ =  0.44329 | **NEB:** None |
| **Branch-specific: Mouse** |  |  |  |
| Model A | 4 | p_0_ =   0.42486, p_1_ =  0.57514,  (p_2_ = 0, p_3_ = 0), w_0_ =  0.12495, w_1_ = 1, w_2_ = 1 | **NEB:** None **BEB:** None |
| Model A null | 3 | p_0_ = 0.42486 , p_1_ = 0.57514,  (p_2_ = 0, p_3_ = 0), w_0_ =  0.12495, w_1_ = 1, w_2_ = 1 | Not allowed |
| Model B | 5 | p_0_ = 0.33716, p_1_ = 0.25582 ,  (p_2_ = 0.23142 , p_3_ = 0.17560),  w_0_ = 0.24563, w_1_ =  1.95234, w_2_ = 0 | **NEB:** None |

***Likelihood Ratio Tests for Porimin:***

| **Comparison** | **Null Model lnL** | **Alt Model lnL** | ***df*** | **Adjusted deltaL** | **Critical Value** | **Significant?** |
| --- | --- | --- | --- | --- | --- | --- |
| ***Site Analysis*** |  |  |  |  |  |  |
| m0 v m3Discrtk2 | -3531.807465 | -3450.780903 | 2 | 162.053124 | 5.99 | YES |
| m3Discrtk2 v m3Discrtk3 | -3450.780903 | -3425.484737 | - | 25.296166 | 1 | YES |
| m1Neutral v m2Selection | -3457.912676 | -3425.762565 | 2 | 64.300222 | 5.99 | YES |
| m7 v m8 | -3460.199984 | -3425.0665 | 2 | 70.266968 | 5.99 | YES |
| m8a v m8 | -3456.92084 | -3425.0665 | 1 | 63.70868 | 2.71 | YES |
| ***Branch Analysis*** |  |  |  |  |  |  |
| ***Human*** |  |  |  |  |  |  |
| ModelA v m1Neutral | -3457.912676 | -3457.912676 | 2 | 0 | 5.99 | NO |
| ModelA v ModelAnull | -3457.912676 | -3457.912676 | 1 | 0 | 3.84 | NO |
| ModelB v m3Discrtk2 | -3449.853993 | -3454.648196 | 2 | 9.588406 | 5.99 | YES |
| ***Chimp*** |  |  |  |  |  |  |
| ModelA v m1Neutral | -3457.912676 | -3457.912676 | 2 | 0 | 5.99 | NO |
| ModelA v ModelAnull | -3457.912676 | -3457.912676 | 1 | 0 | 3.84 | NO |
| ModelB v m3Discrtk2 | -3449.287783 | -3454.648196 | 2 | 10.720826 | 5.99 | YES |
| ***Macaque*** |  |  |  |  |  |  |
| ModelA v m1Neutral | -3457.912676 | -3457.912676 | 2 | 0 | 5.99 | NO |
| ModelA v ModelAnull | -3457.912676 | -3457.912676 | 1 | 0 | 3.84 | NO |
| ModelB v m3Discrtk2 | 3450.484142 | -3454.648196 | 2 | 8.328108 | 5.99 | YES |
| ***Primates*** |  |  |  |  |  |  |
| ModelA v m1Neutral | -3457.912676 | -3457.912676 | 2 | 0 | 5.99 | NO |
| ModelA v ModelAnull | -3457.912676 | -3457.912676 | 1 | 0 | 3.84 | NO |
| ModelB v m3Discrtk2 | -3450.762138 | -3454.648196 | 2 | 7.772116 | 5.99 | YES |
| ***Mouse*** |  |  |  |  |  |  |
| ModelA v m1Neutral | -3457.912676 | -3457.912676 | 2 | 0 | 5.99 | NO |
| ModelA v ModelAnull | -3457.912676 | -3457.912676 | 1 | 0 | 3.84 | NO |
| ModelB v m3Discrtk2 | -3449.813378 | -3454.648196 | 2 | 9.669636 | 5.99 | YES |
| ***Rodents*** |  |  |  |  |  |  |
| ModelA v m1Neutral | -3456.595524 | -3457.912676 | 2 | 2.634304 | 5.99 | NO |
| ModelA v ModelAnull | -3456.595524 | -3456.686416 | 1 | 0.181784 | 3.84 | NO |
| ModelB v m3Discrtk2 | -3446.023598 | -3454.648196 | 2 | 17.249196 | 5.99 | YES |

**(g) Complete set of estimates for Prkar2a from codeml:**

| **Model** | **P** | **Estimates of parameters** | **Positively**  **selected sites** |
| --- | --- | --- | --- |
| M0 : one ratio | 1 | w = 0.1702 | **NEB:** None **BEB:** None |
| **Site-specific:**    M1:Neutral | 2 | p_0_= 0.82433, w_0_<1 | Not allowed |
| M2:Selection | 4 | p_0_=0.82382, p_1_= 0.14886  (p_2_=0.02732 ), w_0_<1, w_1_ = 1, w_2_ =3.69285 | **NEB:** 11>0.50, 1>0.95 **BEB:** 12>0.50, 1>0.95 |
| M3:Discrete(K = 2) | 3 | p_0_= 0.83396 , (p_1_= 0.16604)  w_0_= 0.04367, w_1_= 1.16177 | **NEB:** 63>0.50, 49>0.95, 40>0.99 |
| M3:Discrete(K = 3) | 5 | p_0_= 0.80399, p_1_= 0.15766 , (p_2_=0.03835)  w_0_= 0.03588, w_1_=0.76687, w_2_= 3.06009 | **NEB:** 14>0.50, 4>0.95, 1>0.99 |
| M7: Beta | 2 | p=0.11561 , q=0.48352 | Not allowed |
| M8: Beta&Omega > 1 | 4 | p_0_= 0.95102, p =0.16339, q =0.98823  (p_1_= 0.04898), w =2.60992 | **NEB:** 14>0.50, 4>0.95, 1>0.99 **BEB:** 19>0.50, 4>0.95 |
| M8a: Beta&Omega = 1 | 3 | p_0_= 0.83787 , p =0.66680, q =  13.25913  (p_1_= 0.16213), w = 1 | Not allowed |
| **Branch-specific: Human** |  |  |  |
| Model A | 4 | p_0_ =   0.68624, p_1_ = 0.14624,  (p_2_ = 0.13809, p_3_ = 0.02943),  w_0_ =   0.03941, w_1_ = 1, w_2_ = 1.05712 | **NEB:** 0>0.50  **BEB:** 0>0.50 |
| Model A null | 3 | p_0_ = 0.68604, p_1_ = 0.14620 ,  (p_2_ = 0.13829, p_3_ =  0.02947) ,  w_0_ =  0.03941 , w_1_ = 1, w_2_ = 1 | Not allowed |
| Model B | 5 | p_0_ = 0.69630, p_1_ = 0.13863  (p_2_ =  0.13765 , p_3_ = 0.02741),  w_0_ = 0.04367, w_1_ =  1.16178, w_2_ = 1.03491 | **NEB:** 0>0.50 |
| **Branch-specific: Chimp** |  |  |  |
| Model A | 4 | p_0_ =  0.67905 , p_1_ =0.14539 ,  (p_2_ = 0.14460, p_3_ = 0.03096),  w_0_ =   0.03868, w_1_ = 1, w_2_ = 1 | **NEB:** None **BEB:** None |
| Model A null | 3 | p_0_ =0.67907 , p_1_ = 0.14539,  (p_2_ =0.14458 , p_3_ = 0.03095),  w_0_ =  0.03868, w_1_ = 1, w_2_ = 1 | Not allowed |
| Model B | 5 | p_0_ =   0.61758, p_1_ = 0.12369 ,  (p_2_ = 0.21556  , p_3_ = 0.04317),  w_0_ = 0.04286, w_1_ = 1.15810, w_2_ = 0.66314 | **NEB:** None |
| **Branch-specific: Macaque** |  |  |  |
| Model A | 4 | p_0_ = 0.81640, p_1_ = 0.15987,  (p_2_ = 0.01984, p_3_ = 0.00389),  w_0_ =0.03897, w_1_ = 1, w_2_ = 999.0 | **NEB:** 9>0.50, 8>0.95 7>0.99 **BEB:** 9>0.50 5>0.95, 3>0.99 |
| Model A null | 3 | p_0_ =  0.74151, p_1_ = 0.14779,  (p_2_ =  0.09230, p_3_ = 0.01840),  w_0_ = 0.03801, w_1_ = 1, w_2_ = 1 | Not allowed |
| Model B | 5 | p_0_ = 0.82834, p_1_ = 0.14634 ,  (p_2_ = 0.02152 , p_3_ = 0.00380),  w_0_ = 0.04446, w_1_ =1.21729, w_2_ = 999.0 | **NEB:** 9>0.50, 8>0.95 7>0.99 |
| **Branch-specific: Primate** |  |  |  |
| Model A | 4 | p_0_ =  0.82427  , p_1_ = 0.17566,  (p_2_ = 0.00006, p_3_ = 0.00001),  w_0_ = 0.03941, w_1_ = 1, w_2_ = 1 | **NEB:** None **BEB:** None |
| Model A null | 3 | p_0_ = 0.82320, p_1_ = 0.17543,  (p_2_ = 0.00113 , p_3_ = 0.00024),  w_0_ = 0.03941 , w_1_ = 1, w_2_ = 1 | Not allowed |
| Model B | 5 | p_0_ =  0.63801, p_1_ = 0.12703,  (p_2_ = 0.19595 , p_3_ = 0.03901),  w_0_ = 0.04367, w_1_ = 1.16177, w_2_ = 0 | **NEB:** None |
| **Branch-specific: Mouse** |  |  |  |
| Model A | 4 | p_0_ = 0.82433, p_1_ = 0.17567,  (p_2_ = 0, p_3_ = 0),  w_0_ =  0.03941, w_1_ = 1, w_2_ = 1 | **NEB:** None **BEB:** None |
| Model A null | 3 | p_0_ =  0.82433, p_1_ = 0.17567,  (p_2_ =  0, p_3_ = 0)  w_0_ =  0.03941, w_1_ = 1, w_2_ = 1 | Not allowed |
| Model B | 5 | p_0_ =  0.34272, p_1_ = 0.06745,  (p_2_ = 0.49284, p_3_ = 0.09699) ,  w_0_ = 0.04509  , w_1_ = 1.22455, w_2_ =  0.01827 | **NEB:** None |
| **Branch-specific: Rodent** |  |  |  |
| Model A | 4 | p_0_ =   0.82433 , p_1_ = 0.17567,  (p_2_ = 0, p_3_ = 0),  w_0_ = 0.03941, w_1_ = 1, w_2_ = 1 | **NEB:** None **BEB:** None |
| Model A null | 3 | p_0_ = 0.82410  , p_1_ = 0.17562,  (p_2_ = 0.00024, p_3_ = 0.00005)  w_0_ = 0.03941, w_1_ = 1, w_2_ = 1 | Not allowed |
| Model B | 5 | p_0_ =  0.83396 , p_1_ = 0.16604 , (p_2_ = 0,  p_3_ = 0),  w_0_ = 0.04367, w_1_ = 1.16177, w_2_ = 0 | **NEB:** None |
| **Branch-specific: Gleries** |  |  |  |
| Model A | 4 | p_0_ = 0.82419, p_1_ = 0.17563,(p_2_ = 0.00015 , p_3_ = 0.00003),w_0_ = 0.03940, w_1_= 1, w_2_ = 1 | **NEB:** None **BEB:** None |
| Model A null | 3 | p_0_ = 0.82433, p_1_ = 0.17567,(p_2_ = 0, p_3_ = 0),w_0_ =  0.03941, w_1_= 1, w_2_ = 1 | Not allowed |
| Model B | 5 | p_0_ =  0.83396, p_1_ = 0.16604,(p_2_ = 0 , p_3_ = 0),w_0_ =  0.04367 , w_1_ = 1.16178, w_2_ = 0 | **NEB:** None |

***Likelihood Ratio Tests for Prkar2a:***

| **Comparison** | **Null Model lnL** | **Alt Model lnL** | ***df*** | **Adjusted deltaL** | **Critical Value** | **Significant?** |
| --- | --- | --- | --- | --- | --- | --- |
| ***Site Analysis*** |  |  |  |  |  |  |
| m0 v m3Discrtk2 | -5556.273714 | -5355.949957 | 2 | 400.647514 | 5.99 | YES |
| m3Discrtk2 v m3Discrtk3 | -5355.949957 | -5347.614731 | - | 8.335226 | 1 | YES |
| m1Neutral v m2Selection | -5356.92345 | -5348.913336 | 2 | 16.020228 | 5.99 | YES |
| m7 v m8 | -5365.685274 | -5350.657546 | 2 | 30.055456 | 5.99 | YES |
| m8a v m8 | -5355.804613 | -5350.657546 | 1 | 10.294134 | 2.71 | YES |
| ***Branch Analysis*** |  |  |  |  |  |  |
| ***Human*** |  |  |  |  |  |  |
| ModelA v m1Neutral | -5356.923464 | -5356.92345 | 2 | 2.8E-05 | 5.99 | NO |
| ModelA v ModelAnull | -5356.923464 | -5356.923463 | 1 | 1E-06 | 3.84 | NO |
| ModelB v m3Discrtk2 | -5355.94997 | -5355.949957 | 2 | 2.6E-05 | 5.99 | NO |
| ***Chimp*** |  |  |  |  |  |  |
| ModelA v m1Neutral | -5356.189374 | -5356.92345 | 2 | 1.468152 | 5.99 | NO |
| ModelA v ModelAnull | -5356.189374 | -5356.189374 | 1 | 0 | 3.84 | NO |
| ModelB v m3Discrtk2 | -5355.260357 | -5355.949957 | 2 | 1.3792 | 5.99 | NO |
| ***Macaque*** |  |  |  |  |  |  |
| ModelA v m1Neutral | -5310.912882 | -5356.92345 | 2 | 92.021136 | 5.99 | YES |
| ModelA v ModelAnull | -5310.912882 | -5351.943651 | 1 | 82.061538 | 3.84 | YES |
| ModelB v m3Discrtk2 | -5309.213976 | -5355.949957 | 2 | 93.471962 | 5.99 | YES |
| ***Mouse*** |  |  |  |  |  |  |
| ModelA v m1Neutral | -5356.923451 | -5356.92345 | 2 | 2E-06 | 5.99 | NO |
| ModelA v ModelAnull | -5356.923451 | -5356.923451 | 1 | 0 | 3.84 | NO |
| ModelB v m3Discrtk2 | -5354.063074 | -5355.949957 | 2 | 3.773766 | 5.99 | NO |
| ***Primates*** |  |  |  |  |  |  |
| ModelA v m1Neutral | -5356.92345 | -5356.92345 | 2 | 0 | 5.99 | NO |
| ModelA v ModelAnull | -5356.92345 | -5356.923453 | 1 | 6E-06 | 3.84 | NO |
| ModelB v m3Discrtk2 | -5355.949948 | -5355.949957 | 2 | 1.8E-05 | 5.99 | NO |
| ***Rodents*** |  |  |  |  |  |  |
| ModelA v m1Neutral | -5356.923451 | -5356.92345 | 2 | 2E-06 | 5.99 | NO |
| ModelA v ModelAnull | -5356.923451 | -5356.924649 | 1 | 0.002396 | 3.84 | NO |
| ModelB v m3Discrtk2 | -5355.949957 | -5355.949957 | 2 | 0 | 5.99 | NO |
| ***Gleries*** |  |  |  |  |  |  |
| ModelA v m1Neutral | -5356.924709 | -5356.92345 | 2 | 0.002518 | 5.99 | NO |
| ModelA v ModelAnull | -5356.924709 | -5356.923450 | 1 | 0.002518 | 3.84 | NO |
| ModelB v m3Discrtk2 | -5355.949957 | -5355.949957 | 2 | 0 | 5.99 | NO |

**(h) Complete set of estimates for Semg2 from codeml:**

| **Model** | **P** | **Estimates of parameters** | **Positively**  **selected sites** |
| --- | --- | --- | --- |
| M0 : one ratio | 1 | w =1.0641 | **NEB:** 0>0.50  **BEB:** 0>0.50 |
| **Site-specific:**  M1:Neutral | 2 | p_0_=0.38310, w_0_<1 | Not allowed |
| M2:Selection | 4 | p_0_= 0.32252, p_1_= 0.65009  (p_2_= 0.02739), w_0_<1, w_1_ = 1, w_2_ = 11.91464 | **NEB:** 9>0.50, 3>0.95, 2>0.99 **BEB:** 12>0.50, 3>0.95, 1>0.99 |
| M3:Discrete(K = 2) | 3 | p_0_=  0.66349, (p_1_= 0.33651)  w_0_=0.20188, w_1_= 3.08834 | **NEB:** 114>0.50, 41>0.95, 12>0.99 |
| M3:Discrete(K = 3) | 5 | p_0_=0.49325, p_1_=0.49341, (p_2_=0.01334)  w_0_= 0.00, w_1_=2.06418 , w_2_= 23.98764 | **NEB:** 119>0.50, 115>0.95, 115>0.99 |
| M7: Beta | 2 | p= 0.01164, q= 0.00500 | Not allowed |
| M8: Beta&Omega > 1 | 4 | p_0_=  0.97236 , p =0.01163, q = 0.00500  (p_1_= 0.02764), w =12.26405 | **NEB**: 9>0.50, 3>0.95, 3>0.99 **BEB:** 41>0.50, 5>0.95, 3>0.99 |
| M8a: Beta&Omega = 1 | 3 | p_0_=  0.38310, p = 0.00500, q =  14.26940  (p_1_= 0.61690), w = 1 | Not allowed |
| **Branch-specific: Human** |  |  |  |
| Model A | 4 | p_0_ =  0.38310, p_1_ = 0.61690,  (p_2_ = 0, p_3_ = 0), w_0_ =  0, w_1_ = 1, w_2_ = 1 | **NEB:** None **BEB:** None |
| Model A null | 3 | p_0_ = 0.38310, p_1_ = 0.61690,  (p_2_ = 0, p_3_ = 0), w_0_ =  0, w_1_ = 1, w_2_ = 1 | Not allowed |
| Model B | 5 | p_0_ = 0.21303 , p_1_ = 0.11303,  (p_2_ =  0.44032, p_3_ = 0.23362),  w_0_ = 0.17569 , w_1_ =  3.20350, w_2_ = 0.3217 | **NEB:** None |
| **Branch-specific: Chimp** |  |  |  |
| Model A | 4 | p_0_ =  0.38310, p_1_ = 0.61690,  (p_2_ = 0, p_3_ = 0), w_0_ =  0, w_1_ = 1, w_2_ = 1 | **NEB:** None **BEB:** None |
| Model A null | 3 | p_0_ = 0.38310, p_1_ = 0.61690,  (p_2_ = 0, p_3_ = 0), w_0_ =  0, w_1_ = 1, w_2_ = 1 | Not allowed |
| Model B | 5 | p_0_ = 0.22146 , p_1_ =  0.11226 ,  (p_2_ = 0.44216, p_3_ = 0.22412),  w_0_ = 0.20426, w_1_ = 3.12611, w_2_ = 0 | **NEB:** None |
| **Branch-specific: Gibbon** |  |  |  |
| Model A | 4 | p_0_ = 0.37508 , p_1_ = 0.58617,  (p_2_ = 0.01512, p_3_ =  0.02363),  w_0_ =  0, w_1_ = 1, w_2_ =  3.98917 | **NEB:** 0>0.50 **BEB** 0>0.50 |
| Model A null | 3 | p_0_ = 0.38310 , p_1_ = 0.61690,  (p_2_ = 0, p_3_ = 0), w_0_ =  0, w_1_ = 1, w_2_ = 1 | Not allowed |
| Model B | 5 | p_0_ = 0.42329, p_1_ = 0.19930,  (p_2_ = 0.25660  , p_3_ = 0.12082),  w_0_ = 0.20510 , w_1_ =  3.26832, w_2_ = 0.90879 | **NEB:** None |
| **Branch-specific: Macaque** |  |  |  |
| Model A | 4 | p_0_ = 0.38126 , p_1_ = 0.60914,  (p_2_ = 0.00369, p_3_ =0.00590),  w_0_ =  0, w_1_ = 1, w_2_ = 5.32751 | **NEB:** 0>0.50 **BEB:** 0>0.50 |
| Model A null | 3 | p_0_ =  0.38310  , p_1_ = 0.61690,  (p_2_ = 0, p_3_ = 0), w_0_ =  0, w_1_ = 1, w_2_ = 1 | Not allowed |
| Model B | 5 | p_0_ =   0.64582 , p_1_ = 0.32351,  (p_2_ = 0.02043, p_3_ = 0.01023),  w_0_ =  0.20187, w_1_ = 3.10553, w_2_ = 1.95963 | **NEB:** 0>0.50 |
| **Branch-specific: Great Apes** |  |  |  |
| Model A | 4 | p_0_ =  0.01190 , p_1_ =  0.01828,  (p_2_ = 0.38227, p_3_ = 0.58755), w_0_ =  0, w_1_ = 1, w_2_ = 560.61630 | **NEB:** 411>0.50, 11>0.95, 3>0.99 **BEB:** 0>0.50 |
| Model A null | 3 | p_0_ = 0, p_1_ = 0,  (p_2_ =0.39448, p_3_ = 0.60552),  w_0_ =  0, w_1_ = 1, w_2_ = 1 | Not allowed |
| Model B | 5 | p_0_ = 0, p_1_ = 0, (p_2_ = 0.69371 p_3_ = 0.30629)  w_0_ = 0.23221  w_1_ = 3.22380, w_2_ = 999.0 | **NEB:** 411>0.50, 411>0.95, 3>0.99 |

***Likelihood Ratio Tests for Semg2:***

| **Comparison** | **Null Model lnL** | **Alt Model lnL** | ***df*** | **Adjusted deltaL** | **Critical Value** | **Significant?** |
| --- | --- | --- | --- | --- | --- | --- |
| ***Site Analysis*** |  |  |  |  |  |  |
| m0 v m3Discrtk2 | -2967.008783 | -2941.937033 | 2 | 50.1435 | 5.99 | YES |
| m3Discrtk2 v m3Discrtk3 | -2941.937033 | -2935.293302 | - | 6.643731 | 1 | YES |
| m1Neutral v m2Selection | -2955.125231 | -2940.671045 | 2 | 28.908372 | 5.99 | YES |
| m7 v m8 | -2955.947411 | -2940.757151 | 2 | 30.38052 | 5.99 | YES |
| m8a v m8 | -2955.125215 | -2940.757151 | 1 | 28.736128 | 2.71 | YES |
| ***Branch Analysis*** |  |  |  |  |  |  |
| ***Human*** |  |  |  |  |  |  |
| ModelA v m1Neutral | -2955.125231 | -2955.125231 | 2 | 0 | 5.99 | NO |
| ModelA v ModelAnull | -2955.125231 | -2955.125231 | 1 | 0 | 3.84 | NO |
| ModelB v m3Discrtk2 | -2941.105158 | -2941.937033 | 2 | 1.66375 | 5.99 | NO |
| ***Chimp*** |  |  |  |  |  |  |
| ModelA v m1Neutral | -2955.125231 | -2955.125231 | 2 | 0 | 5.99 | NO |
| ModelA v ModelAnull | -2955.125231 | -2955.125231 | 1 | 0 | 3.84 | NO |
| ModelB v m3Discrtk2 | -2941.658024 | -2941.937033 | 2 | 0.558018 | 5.99 | NO |
| ***Macaque*** |  |  |  |  |  |  |
| ModelA v m1Neutral | -2955.117673 | -2955.125231 | 2 | 0.015116 | 5.99 | NO |
| ModelA v ModelAnull | -2955.117673 | -2955.125231 | 1 | 0.015116 | 3.84 | NO |
| ModelB v m3Discrtk2 | -2941.927647 | -2941.937033 | 2 | 0.018772 | 5.99 | NO |
| ***Gibbon*** |  |  |  |  |  |  |
| ModelA v m1Neutral | -2955.044674 | -2955.125231 | 2 | 0.161114 | 5.99 | NO |
| ModelA v ModelAnull | -2955.044674 | -2955.125231 | 1 | 0.161114 | 3.84 | NO |
| ModelB v m3Discrtk2 | -2941.578651 | -2941.937033 | 2 | 0.716764 | 5.99 | NO |
| ***Great Apes*** |  |  |  |  |  |  |
| ModelA v m1Neutral | -2953.710406 | -2955.125231 | 2 | 2.82965 | 5.99 | NO |
| ModelA v ModelAnull | -2953.710406 | -2954.526953 | 1 | 1.633094 | 3.84 | NO |
| ModelB v m3Discrtk2 | -2940.560072 | -2941.937033 | 2 | 2.753922 | 5.99 | NO |

**(i)** **Complete set of estimates for SP56 from codeml:**

| **Model** | **P** | **Estimates of parameters** | **Positively**  **selected sites** |
| --- | --- | --- | --- |
| M0 : one ratio | 1 | w = 0.1138 | **NEB:** None **BEB:** None |
| **Site-specific:**    M1:Neutral | 2 | p_0_= 0.83328, w_0_<1 | Not allowed |
| M2:Selection | 4 | p_0_= 0.82941, p_1_=  0.16297  (p_2_= 0.00762), w_0_<1, w_1_ = 1, w_2_ =  5.47520 | **NEB:** 3>0.50, 2>0.95, 1>0.99 **BEB:** 5>0.50, 2>0.95, 2>0.99 |
| M3:Discrete(K = 2) | 3 | p_0_=  0.76649,  (p_1_= 0.23351)  w_0_= 0.01604, w_1_=  0.54550 | **NEB:** 0>0.50 |
| M3:Discrete(K = 3) | 5 | p_0_=  0.73297, p_1_=  0.25204,  (p_2_=  0.01498 )  w_0_= 0.01160, w_1_=0.42179, w_2_=3.42528 | **NEB:** 7>0.50, 3>0.50, 2>0.50 |
| M7: Beta | 2 | p= 0.13262, q=  q=0.75478 | Not allowed |
| M8: Beta&Omega > 1 | 4 | p_0_=0.98807   , p = 0.16114, q = 1.12262  (p_1_= 0.01193), w =3.81710 | **NEB:** 6>0.50, 3>0.95, 2>0.99 **BEB:** 8>0.50, 2>0.95, 2>0.99 |
| M8a: Beta&Omega = 1 | 3 | p_0_=  0.94946, p = 0.17834 , q =  1.69928  (p_1_= 0.05054), w = 1 | Not allowed |
| **Branch-specific: Human** |  |  |  |
| Model A | 4 | p_0_ = 0, p_1_ = 0,  (p_2_ = 0.83165 , p_3_ = 0.16835),  w_0_ =  0.03462 , w_1_ = 1, w_2_ = 48.8805 | **NEB:** 472>0.50, 472>0.95, 472>0.99 **BEB** 1>0.50, 1>0.95 |
| Model A null | 3 | p_0_ = 0, p_1_ = 0,  (p_2_ = 0.83164 , p_3_ = 0.16836),  w_0_ =  0.03462, w_1_ = 1, w_2_ = 1 | Not allowed |
| Model B | 5 | p_0_ = 0, p_1_ = 0, (p_2_ = 0.76604, p_3_ = 0.23396),  w_0_ = 0.01545, w_1_ =  0.54490,  w_2_ =  62.40015 | **NEB:** 472>0.50, 472>0.95, 472>0.99 |
| **Branch-specific: Chimp** |  |  |  |
| Model A | 4 | p_0_ = 0.80249, p_1_ = 0.16131 ,  (p_2_ = 0.03014, p_3_ = 0.00606),  w_0_ =  0.03519, w_1_ = 1, w_2_ = 1 | **NEB:** None **BEB:** None |
| Model A null | 3 | p_0_ = 0.80249, p_1_ = 0.16131,  (p_2_ = 0.03014 , p_3_ = 0.00606),  w_0_ =  0.03519, w_1_ = 1, w_2_ = 1 | Not allowed |
| Model B | 5 | p_0_ = 0.62552 , p_1_ =  0.19108 ,  (p_2_ = 0.14048 , p_3_ =  0.04291),  w_0_ = 0.01562, w_1_=0.54531, w_2_ = 0.24929 | **NEB:** None |
| **Branch-specific: Primates** |  |  |  |
| Model A | 4 | p_0_ = 0.83328, p_1_ = 0.16672,  (p_2_ = 0, p_3_ = 0),  w_0_ =  0.03562 , w_1_ = 1, w_2_ = 1 | **NEB:** None **BEB:** None |
| Model A null | 3 | p_0_ =   0.83328, p_1_ = 0.16672,  (p_2_ = 0, p_3_ = 0),  w_0_ =  0.03562, w_1_ = 1, w_2_ = 1 | Not allowed |
| Model B | 5 | p_0_ = 0.38342, p_1_ = 0.11670,  (p_2_ = 0.38324, p_3_ = 0.11664),  w_0_ =0.01628 , w_1_ = 0.55447, w_2_ = 0 | **NEB:** None |
| **Branch-specific: Rat** |  |  |  |
| Model A | 4 | p_0_ = 0.83328, p_1_ = 0.16672,  (p_2_ = 0, p_3_ = 0),  w_0_ =  0.03562, w_1_ = 1, w_2_ = 1 | **NEB:** None **BEB:** None |
| Model A null | 3 | p_0_ =  0.83328, p_1_ = 0.16672  ,  (p_2_ = 0, p_3_ =0),  w_0_ = 0.03562 , w_1_ = 1, w_2_ = 1 | Not allowed |
| Model B | 5 | p_0_ =  0.59285, p_1_ =  0.18131 ,  (p_2_ =0.17295, p_3_ =  0.05289),  w_0_ = 0.01642, w_1_ =0.56302, w_2_ = 0 | **NEB:** None |
| **Branch-specific: Gleries** |  |  |  |
| Model A | 4 | p_0_ = 0.79879, p_1_ = 0.16079,  (p_2_ = 0.03364, p_3_ =0.00677 ),  w_0_ =  0.03470, w_1_ = 1, w_2_ = 1 | **NEB:** None **BEB:** None |
| Model A null | 3 | p_0_ = 0.79880 , p_1_ = 0.16079,  (p_2_ = 0.03364 , p_3_ = 0.00677),  w_0_ =  0.03470, w_1_ = 1, w_2_ = 1 | Not allowed |
| Model B | 5 | p_0_ = 0.74743, p_1_ = 0.22662 ,  (p_2_ =0.01991, p_3_ = 0.00604),  w_0_ =  0.01584, w_1_ =0.54725 w_2_ = 1.02713 | **NEB:** None |

***Likelihood Ratio Tests for SP56:***

| **Comparison** | **Null Model lnL** | **Alt Model lnL** | ***df*** | **Adjusted deltaL** | **Critical Value** | **Significant?** |
| --- | --- | --- | --- | --- | --- | --- |
| ***Site Analysis*** |  |  |  |  |  |  |
| m0 v m3Discrtk2 | -6552.992663 | -6367.942407 | 2 | 370.100512 | 5.99 | YES |
| m3Discrtk2 v m3Discrtk3 | -6367.942407 | -6344.10859 | - | 23.833817 | 1 | YES |
| m1Neutral v m2Selection | -6388.017894 | -6378.223309 | 2 | 19.58917 | 5.99 | YES |
| m7 v m8 | -6359.47666 | -6344.832366 | 2 | 29.288588 | 5.99 | YES |
| m8a v m8 | -6356.733864 | -6344.832366 | 1 | 23.802996 | 2.71 | YES |
| ***Branch Analysis*** |  |  |  |  |  |  |
| ***Human*** |  |  |  |  |  |  |
| ModelA v m1Neutral | -6385.540328 | -6388.017894 | 2 | 4.955132 | 5.99 | NO |
| ModelA v ModelAnull | -6385.540328 | -6385.767844 | 1 | 0.227516 | 3.84 | NO |
| ModelB v m3Discrtk2 | -6365.078427 | -6378.223309 | 2 | 26.289764 | 5.99 | YES |
| ***Chimp*** |  |  |  |  |  |  |
| ModelA v m1Neutral | -6387.863284 | -6388.017894 | 2 | 0.30922 | 5.99 | NO |
| ModelA v ModelAnull | -6387.863284 | -6387.863284 | 1 | 0 | 3.84 | NO |
| ModelB v m3Discrtk2 | -6367.537145 | -6378.223309 | 2 | 21.372328 | 5.99 | YES |
| ***Primate*** |  |  |  |  |  |  |
| ModelA v m1Neutral | -6388.017894 | -6388.017894 | 2 | 0 | 5.99 | NO |
| ModelA v ModelAnull | -6388.017894 | -6388.017894 | 1 | 0 | 3.84 | NO |
| ModelB v m3Discrtk2 | -6367.453226 | -6378.223309 | 2 | 21.540166 | 5.99 | YES |
| ***Rat*** |  |  |  |  |  |  |
| ModelA v m1Neutral | -6388.017894 | -6388.017894 | 2 | 0 | 5.99 | NO |
| ModelA v ModelAnull | -6388.017894 | -6388.017894 | 1 | 0 | 3.84 | NO |
| ModelB v m3Discrtk2 | -6367.011208 | -6378.223309 | 2 | 22.424202 | 5.99 | YES |
| ***Gleries*** |  |  |  |  |  |  |
| ModelA v m1Neutral | -6387.711015 | -6388.017894 | 2 | 0.613758 | 5.99 | NO |
| ModelA v ModelAnull | -6387.711015 | -6387.711015 | 1 | 0 | 3.84 | NO |
| ModelB v m3Discrtk2 | -6367.692232 | -6378.223309 | 2 | 21.062154 | 5.99 | YES |

**(j) Complete set of estimates for ZP2 from codeml:**

| **Model** | **P** | **Estimates of parameters** | **Positively**  **selected sites** |
| --- | --- | --- | --- |
| M0 : one ratio | 1 | w = 0.5851 | **NEB:** None **BEB:** None |
| **Site-specific:**    M1:Neutral | 2 | p_0_= 0.52875, w_0_<1 | Not allowed |
| M2:Selection | 4 | p_0_= 0.49854, p_1_=0.43661  (p_2_= 0.06485), w_0_<1,  w_1_ = 1, w_2_ =  2.66652 | **NEB:** 37> 0.50, 9>0.95, 5>0.99 **BEB:** 38>0.50, 7>0.95, 5>0.99 |
| M3:Discrete(K = 2) | 3 | p_0_=  0.60760, (p_1_= 0.39240)  w_0_= 0.23102, w_1_= 1.28434 | **NEB:** 270>0.50, 156>0.95, 127>0.99 |
| M3:Discrete(K = 3) | 5 | p_0_= 0.35030, p_1_= 0.48387 , (p_2_=0.16583)  w_0_= 0.09764 , w_1_=0.64952, w_2_=1.89310 | **NEB:** 270>0.50, 156>0.95, 124>0.99 |
| M7: Beta | 2 | p=0.45862, q= 0.41203 | Not allowed |
| M8: Beta&Omega > 1 | 4 | p_0_=  0.87339, p =0.63945 , q = 0.75356  (p_1_=0.12661), w =2.04655 | **NEB:** 78>0.50, 15>0.95, 7>0.99 **BEB:** 59>0.50, 9>0.95, 6>0.99 |
| M8a: Beta&Omega = 1 | 3 | p_0_=0.59796, p =1.33725, q =  4.63739  (p_1_=0.40204), w = 1 | Not allowed |
| **Branch-specific: Human** |  |  |  |
| Model A | 4 | p_0_ =  0.00646, p_1_ =0.00577,  (p_2_ = 0.52184, p_3_ = 0.46593), w_0_ =  0.17071, w_1_ = 1, w_2_ = 129.20059 | **NEB:** 721>0.50, 721>0.95, 2>0.99 **BEB** : 721>0.50 |
| Model A null | 3 | p_0_ = 0, p_1_ = 0,  (p_2_ = 0.52832 , p_3_ = 0.47168), w_0_ =  0.17072, w_1_ = 1, w_2_ = 1 | Not allowed |
| Model B | 5 | p_0_ = 0, p_1_ = 0, (p_2_ = 0.60601, p_3_ = 0.39399),  w_0_ = 0.22951,  w_1_ = 1.28055, w_2_ = 304.30581 | **NEB:** 721>0.50, 721>0.95, 721>0.99 |
| **Branch-specific: Chimp** |  |  |  |
| Model A | 4 | p_0_ = 0.52875 , p_1_ = 0.47125,  (p_2_ = 0, p_3_ = 0),  w_0_ =    0.17142 , w_1_ = 1, w_2_ = 1 | **NEB**: None **BEB**: None |
| Model A null | 3 | p_0_ = 0.52875, p_1_ = 0.47125 ,  (p_2_ = 0, p_3_ = 0),  w_0_ =   0.17142, w_1_ = 1, w_2_ = 1 | Not allowed |
| Model B | 5 | p_0_ = 0.19036, p_1_ =  0.12287,  (p_2_ = 0.41737, p_3_ = 0.26940),  w_0_ = 0.23187, w_1_ = 1.28943, w_2_ = 0 | **NEB**: None |
| **Branch-specific: Macaque** |  |  |  |
| Model A | 4 | p_0_ =  0.52875 , p_1_ =  0.47125,  (p_2_ = 0, p_3_ = 0),  w_0_ =   0.17142, w_1_ = 1, w_2_ = 1 | **NEB**: None **BEB**: None |
| Model A null | 3 | p_0_ = 0.52875 , p_1_ = 0.47125,  (p_2_ = 0, p_3_ = 0),  w_0_ =  0.17142 , w_1_ = 1, w_2_ = 1 | Not allowed |
| Model B | 5 | p_0_ =  0.34814, p_1_ = 0.22492 ,  (p_2_ = 0.25937, p_3_ = 0.16757),  w_0_ =  0.23307, w_1_ =  1.29682,  w_2_ = 0 | **NEB**: None |
| **Branch-specific: Primates** |  |  |  |
| Model A | 4 | p_0_ = 0.52875 , p_1_ = 0.47125,  (p_2_ = 0, p_3_ = 0),  w_0_ =  0.17142, w_1_ = 1, w_2_ = 1 | **NEB**: None **BEB**: None |
| Model A null | 3 | p_0_ =  0.52875 , p_1_ = 0.47125 ,  (p_2_ = 0, p_3_ = 0),  w_0_ =  0.17142, w_1_ = 1, w_2_ = 1 | Not allowed |
| Model B | 5 | p_0_ = 0.60760 ,  p_1_ = 0.39240, (p_2_ = 0, p_3_ = 0),  w_0_ =  0.23102, w_1_ = 1.28434  , w_2_ = 0 | **NEB**: None |
| **Branch-specific: Mouse** |  |  |  |
| Model A | 4 | p_0_ = 0.52491, p_1_ = 0.46777,  (p_2_ = 0.00387, p_3_ = 0.003450),  w_0_ =  0.17129 , w_1_ = 1, w_2_ = 1 | **NEB**: None **BEB**: None |
| Model A null | 3 | p_0_ =  0.52491 , p_1_ = 0.46777,  (p_2_ = 0.00387 , p_3_ = 0.00345),  w_0_ =  0.17129, w_1_ = 1, w_2_ = 1 | Not allowed |
| Model B | 5 | p_0_ = 0.09078 , p_1_ = 0.05550 ,  (p_2_ = 0.52981, p_3_ = 0.32392),  w_0_ = 0.24077, w_1_ =   1.35457, w_2_ = 0.20651 | **NEB**: None |
| **Branch-specific: Rodent** |  |  |  |
| Model A | 4 | p_0_ = 0.52875  , p_1_ = 0.47125 ,  (p_2_ = 0, p_3_ = 0),  w_0_ =  0.17142  , w_1_ = 1, w_2_ = 1 | **NEB**: None **BEB**: None |
| Model A null | 3 | p_0_ = 0.52875, p_1_ = 0.47125,  (p_2_ = 0, p_3_ = 0),  w_0_ =  0.17142, w_1_ = 1, w_2_ = 1 | Not allowed |
| Model B | 5 | p_0_ = 0.60760 , p_1_ = 0.39240,  (p_2_ = 0, p_3_ = 0),  w_0_ = 0.23102, w_1_ = 1.28434, w_2_ = 0 | **NEB**: None |
| **Branch-specific: Gleries** |  |  |  |
| Model A | 4 | p_0_ = 0.52875 , p_1_ = 0.47125, (p_2_ = 0, p_3_ = 0), w_0_ = 0.17142  , w_1_= 1, w_2_ =1 | **NEB**: None **BEB**: None |
| Model A null | 3 | p_0_ = 0.52875  p_1_ = 0.47125, (p_2_ = 0, p_3_ = 0), w_0_ =  0.17142, w_1_= 1, w_2_ = 1 | Not allowed |
| Model B | 5 | p_0_ =   0.60760, p_1_ = 0.39240 , (p_2_ = 0, p_3_ = 0), w_0_ =   0.23102, w_1_ = 1.28434, w_2_ = 1 | **NEB**: None |

***Likelihood Ratio Tests for Zp2:***

| **Comparison** | **Null Model lnL** | **Alt Model lnL** | ***df*** | **Adjusted deltaL** | **Critical Value** | **Significant?** |
| --- | --- | --- | --- | --- | --- | --- |
| ***Site Analysis*** |  |  |  |  |  |  |
| m0 v m3Discrtk2 | -17943.47552 | -17603.98372 | 2 | 678.983612 | 5.99 | YES |
| m3Discrtk2 v m3Discrtk3 | -17603.98372 | -17568.54552 | - | 35.438198 | 1 | YES |
| m1Neutral v m2Selection | -17614.28213 | -17575.47844 | 2 | 77.607378 | 5.99 | YES |
| m7 v m8 | -17623.26626 | -17567.9771 | 2 | 110.57833 | 5.99 | YES |
| m8a v m8 | -17606.26881 | -17567.9771 | 1 | 76.58343 | 2.71 | YES |
| ***Branch Analysis*** |  |  |  |  |  |  |
| ***Human*** |  |  |  |  |  |  |
| ModelA v m1Neutral | -17612.86998 | -17614.28213 | 2 | 2.824302 | 5.99 | NO |
| ModelA v ModelAnull | -17612.86998 | -17613.49354 | 1 | 1.247122 | 3.84 | NO |
| ModelB v m3Discrtk2 | -17602.77289 | -17603.98372 | 2 | 2.421646 | 5.99 | NO |
| ***Chimp*** |  |  |  |  |  |  |
| ModelA v m1Neutral | -17614.28213 | -17614.28213 | 2 | 0 | 5.99 | NO |
| ModelA v ModelAnull | -17614.28213 | -17614.28213 | 1 | 0 | 3.84 | NO |
| ModelB v m3Discrtk2 | -17602.52026 | -17603.98372 | 2 | 2.92691 | 5.99 | NO |
| ***Macaque*** |  |  |  |  |  |  |
| ModelA v m1Neutral | -17614.28213 | -17614.28213 | 2 | 0 | 5.99 | NO |
| ModelA v ModelAnull | -17614.28213 | -17614.282132 | 1 | 0 | 3.84 | NO |
| ModelB v m3Discrtk2 | -17602.14825 | -17603.98372 | 2 | 3.670928 | 5.99 | NO |
| ***Primate*** |  |  |  |  |  |  |
| ModelA v m1Neutral | -17614.28213 | -17614.28213 | 2 | 0 | 5.99 | NO |
| ModelA v ModelAnull | -17614.28213 | -17614.282132 | 1 | 0 | 3.84 | NO |
| ModelB v m3Discrtk2 | -17603.98372 | -17603.98372 | 2 | 0 | 5.99 | NO |
| ***Mouse*** |  |  |  |  |  |  |
| ModelA v m1Neutral | -17614.27981 | -17614.28213 | 2 | 0.004644 | 5.99 | NO |
| ModelA v ModelAnull | 17614.27981 | -17614.279810 | 1 | 0 | 3.84 | NO |
| ModelB v m3Discrtk2 | -17596.42652 | -17603.98372 | 2 | 15.114388 | 5.99 | YES |
| ***Rodents*** |  |  |  |  |  |  |
| ModelA v m1Neutral | -17614.28213 | -17614.28213 | 2 | 0 | 5.99 | NO |
| ModelA v ModelAnull | -17614.28213 | -17614.28213 | 1 | 0 | 3.84 | NO |
| ModelB v m3Discrtk2 | -17603.98372 | -17603.98372 | 2 | 0 | 5.99 | NO |
| ***Gliries*** |  |  |  |  |  |  |
| ModelA v m1Neutral | -17614.28213 | -17614.28213 | 2 | 0 | 5.99 | NO |
| ModelA v ModelAnull | -17614.28213 | -17614.282132 | 1 | 0 | 3.84 | NO |
| ModelB v m3Discrtk2 | -17603.98372 | -17603.98372 | 2 | 0 | 5.99 | NO |

(k) **Complete set of estimates for Zp3 from Codeml:**

| **Model** | **P** | **Estimates of parameters** | **Positively**  **selected sites** |
| --- | --- | --- | --- |
| M0 : one ratio | 1 | w = 0.2942 | **NEB:** None **BEB :** None |
| **Site-specific:**    M1:Neutral | 2 | p_0_= 0.62945, w_0_<1 | Not allowed |
| M2:Selection | 4 | p_0_= 0.62752, p_1_=  0.34283  (p_2_= 0.02965 ), w_0_<1,  w_1_ = 1, w_2_ =  2.72294 | **NEB:** 6>0.50 **BEB:** 6>0.50 |
| M3:Discrete(K = 2) | 3 | p_0_=  0.60846, (p_1_= 0.39154)  w_0_= 0.06037, w_1_=  0.88050 | **NEB:** None |
| M3:Discrete(K = 3) | 5 | p_0_=   0.48596, p_1_=  0.37056 , (p_2_=  0.14348)  w_0_= 0.02736, w_1_=0.45881, w_2_=1.60996 | **NEB** : 55>0.50, 13>0.95, 2>0.99 |
| M7: Beta | 2 | p= 0.23323, q=  0.43003 | Not allowed |
| M8: Beta&Omega > 1 | 4 | p_0_=  0.91489 , p = 0.30029, q =  0.77328  (p_1_= 0.08511), w =1.92305 | **NEB:** 29>0.50 **BEB:** 48>0.50 |
| M8a: Beta&Omega = 1 | 3 | p_0_=  0.73389, p =  0.41997, q =  2.58250  (p_1_= 0.26611), w = 1 | Not allowed |
| **Branch-specific: Human** |  |  |  |
| Model A | 4 | p_0_ = 0.62945, p_1_ = 0.37055,  (p_2_ = 0, p_3_ = 0),  w_0_ = 0.07074, w_1_ = 1, w_2_ = 1 | **NEB:** None **BEB:** None |
| Model A null | 3 | p_0_ = 0.62945,  p_1_ = 0.37055,  (p_2_ = 0, p_3_ = 0),  w_0_ =  0.07074, w_1_ = 1, w_2_ = 1 | Not allowed |
| Model B | 5 | p_0_ = 0.60846, p_1_ = 0.39154, (p_2_ = 0, p_3_ = 0),  w_0_ = 0.06037, w_1_ = 0.8805, w_2_ = 0 | **NEB:** None |
| **Branch-specific: Chimp** |  |  |  |
| Model A | 4 | p_0_ = 0.62945, p_1_ = 0.37055 (p_2_ = 0, p_3_ = 0), w_0_ =0.07074, w_1_ = 1, w_2_ = 1 | **NEB:** None **BEB:** None |
| Model A null | 3 | p_0_ =  0.62945, p_1_ = 0.37055,  (p_2_ = 0, p_3_ = 0),  w_0_ =   0.07074, w_1_ = 1, w_2_ = 1 | Not allowed |
| Model B | 5 | p_0_ = 0.60846, p_1_ = 0.39154, (p_2_ = 0, p_3_ = 0),  w_0_ = 0.06037, w_1_ =0.88050 , w_2_ = 0 | **NEB:** None **BEB:** None |
| **Branch-specific: Macaque** |  |  |  |
| Model A | 4 | p_0_ = 0.62945, p_1_ = 0.37055(p_2_ = 0, p_3_ = 0), w_0_ =  0.07074, w_1_ = 1, w_2_ = 1 | **NEB:** None **BEB:** None |
| Model A null | 3 | p_0_ = 0.62945 , p_1_ =0.37055,  (p_2_ = 0, p_3_ = 0),  w_0_ =   0.07074, w_1_ = 1, w_2_ = 1 | Not allowed |
| Model B | 5 | p_0_ = 0.10253, p_1_ = 0.06587,  (p_2_ = 0.50631, p_3_ =  0.32529),  w_0_ = 0.06103, w_1_ = 0.89043, w_2_ = 0 | **NEB:** None |
| **Branch-specific: Primate** |  |  |  |
| Model A | 4 | p_0_ = 0.62945 , p_1_ = 0.37055 (p_2_ = 0, p_3_ = 0), w_0_ =  0.07074, w_1_ = 1, w_2_ = 1 | **NEB:** None **BEB:** None |
| Model A null | 3 | p_0_ = 0.62945 , p_1_ = 0.37055,  (p_2_ = 0, p_3_ = 0),  w_0_ =   0.07074, w_1_ = 1, w_2_ = 1 | Not allowed |
| Model B | 5 | p_0_ = 0.59717, p_1_ = 0.38351,  (p_2_ = 0.01176, p_3_ =  0.00756),  w_0_ =  0.06012, w_1_ = 0.88290, w_2_ = 0.27875 | **NEB:** None |
| **Branch-specific: Rat** |  |  |  |
| Model A | 4 | p_0_ = 0.60809, p_1_ = 0.35826 (p_2_ = 0.02117, p_3_ = 0.01247), w_0_ =  0.06634, w_1_ = 1, w_2_ = 1 | **NEB:** None **BEB:** None |
| Model A null | 3 | p_0_ = 0.60809, p_1_ =0.35826 (p_2_ = 0.02117, p_3_ = 0.01247), w_0_ =  0.06634, w_1_ = 1, w_2_ = 1 | Not allowed |
| Model B | 5 | p_0_ = 0.49723 , p_1_ = 0.32974,  (p_2_ = 0.10403, p_3_ = 0.06899),  w_0_ = 0.05374, w_1_ = 0.89663 , w_2_ = 0.14627 | **NEB:** None |

***Likelihood Ratio Tests for Zp3:***

| **Comparison** | **Null Model lnL** | **Alt Model lnL** | ***df*** | **Adjusted deltaL** | **Critical Value** | **Significant?** |
| --- | --- | --- | --- | --- | --- | --- |
| ***Site Analysis*** |  |  |  |  |  |  |
| m0 v m3Discrtk2 | -6880.089351 | -6681.63976 | 2 | 396.899182 | 5.99 | YES |
| m3Discrtk2 v m3Discrtk3 | -6681.63976 | -6669.675671 | - | 11.964089 | 1 | YES |
| m1Neutral v m2Selection | -6682.720517 | -6680.813569 | 2 | 3.813896 | 5.99 | NO |
| m7 v m8 | -6676.795 | -6669.317906 | 2 | 14.954188 | 5.99 | YES |
| m8a v m8 | -6673.756883 | -6669.317906 | 1 | 8.877954 | 2.71 | YES |
| ***Branch Analysis*** |  |  |  |  |  |  |
| ***Human*** |  |  |  |  |  |  |
| ModelA v m1Neutral | -6682.720517 | -6682.720517 | 2 | 0 | 5.99 | NO |
| ModelA v ModelAnull | -6682.720517 | -6682.720517 | 1 | 0 | 3.84 | NO |
| ModelB v m3Discrtk2 | -6681.63976 | -6681.639917 | 2 | 0.000314 | 5.99 | NO |
| ***Chimp*** |  |  |  |  |  |  |
| ModelA v m1Neutral | -6682.720517 | -6682.720517 | 2 | 0 | 5.99 | NO |
| ModelA v ModelAnull | -6682.720517 | -6682.720517 | 1 |  | 3.84 | NO |
| ModelB v m3Discrtk2 | -6681.63976 | -6681.639917 | 2 | -  0.000314 | 5.99 | NO |
| ***Macaque*** |  |  |  |  |  |  |
| ModelA v m1Neutral | -6682.720517 | -6682.720517 | 2 | 0 | 5.99 | NO |
| ModelA v ModelAnull | -6682.720517 | -6682.720517 | 1 | 0 | 3.84 | NO |
| ModelB v m3Discrtk2 | -6679.655164 | -6681.639917 | 2 | -  3.969506 | 5.99 | NO |
| ***Primate*** |  |  |  |  |  |  |
| ModelA v m1Neutral | -6682.720517 | -6682.720517 | 2 | 0 | 5.99 | NO |
| ModelA v ModelAnull | -6682.720517 | -6682.720517 | 1 | 0 | 3.84 | NO |
| ModelB v m3Discrtk2 | -6681.628031 | -6681.639917 | 2 | 0.023772 | 5.99 | NO |
| ***Rat*** |  |  |  |  |  |  |
| ModelA v m1Neutral | -6681.986301 | -6682.720517 | 2 | 1.468432 | 5.99 | NO |
| ModelA v ModelAnull | -6681.986301 | -6681.986301 | 1 | 0 | 3.84 | NO |
| ModelB v m3Discrtk2 | -6680.445369 | -6681.639917 | 2 | 2.389096 | 5.99 | NO |
