# Supplementary material for: Positive selection neighboring functionally essential sites and disease-implicated regions of mammalian reproductive proteins
Source: BMC Evol Biol. 2010 Feb 11;10:39. doi: 10.1186/1471-2148-10-39 (PMC2830953; doi:10.1186/1471-2148-10-39)
Supplement: Additional file 7 — Additional Table 7 - Summary of data used in the analysis. Species names, unique identifiers and sequence lengths are given for all data. Summary of data used in the analysis. Species names, unique identifiers for Ensembl (ENS) or Swiss-Prot and database versions are given. The sequence length per species are given for all genes. [file 1471-2148-10-39-S7.DOC]

**Additional Table 7: Summary of data used in the analysis. Species names, unique identifiers and sequence lengths are given for all data.**

| **Species** | **ID** | **Database Version** | **Length A.A.** |
| --- | --- | --- | --- |
| **Adam2 (Taxa 12)** | | | |
| *Homo sapiens* | ENST00000265708 | 49 | 735 |
| Equus caballus | ENSECAT00000000152 | 49 | 741 |
| Erinaceus europaeus | **ENSEEUG00000003704** | 49 | 729 |
| Loxodonta africana | **ENSLAFG00000002139** | 49 | 734 |
| Macaca mulatta | ENSMMUT00000004445 | 49 | 735 |
| Pan troglodytes | ENSPTRT00000037401 | 49 | 735 |
| Pongo pygmaeus | ENSPPYT00000021616 | 49 | 719 |
| Rattus norvegicus | ENSRNOT00000023105 | 49 | 738 |
| Mus musculus | ENSMUST00000022618 | 49 | 735 |
| *Oryctolagus cuniculus* | Q28660: AAA93321 | 57 | 751 |
| *Cavia porcellus* | Q60411: CAA77784_2 | 60 | 735 |
| *Bos taurus* | O77780: AAc62753 | 56 | 745 |
| **Catsper1_Mammalia (Taxa 8)** | | | |
| *Bos taurus* | ENSBTAG00000014720 | 49 | 712 |
| Canis familiaris | **ENSCAFG00000013070** | 49 | 766 |
| Equus caballus | **ENSECAG00000024405** | 49 | 790 |
| Felis catus | **ENSFCAG00000013283** | 49 | 691 |
| Mus musculus | ENSMUST00000043380 | 49 | 686 |
| Rattus norvegicus | **ENSRNOG00000028285** | 49 | 678 |
| Ochotona princeps | **ENSOPRG00000002908** | 49 | 663 |
| Tupaia belangeri | ENSTBEG00000003936 | 49 | 750 |
| **Catsper1_Exon1 (Taxa 16)** | | | |
| *Lemur catta* | Q6TYP8: AAQ95788 | 10 | 360 |
| *Pongo pygmaeus* | Q6TYP9:AAQ95787 | 11 | 443 |
| *Gorilla gorilla* | Q6TYQ0: AAQ95786 | 11 | 405 |
| *Pan paniscus* | Q6TYQ1:AAQ95784 | 10 | 405 |
| *Pan troglodytes* | Q6TYQ2: AAQ95783 | 11 | 405 |
| *Colobus guereza* | Q6TYQ3: AAQ95782 | 10 | 395 |
| *Papio hamadryas* | Q6TYQ4: AAQ95781 | 10 | 387 |
| *Cercopithecus aethiops* | Q6TYQ5: AAQ95780 | 10 | 383 |
| *Macaca mulatta* | Q6TYQ6:AAQ95779 | 11 | 390 |
| *Miopithecus talapoin* | Q6TYQ7:AAQ95778 | 10 | 395 |
| *Saimiri sciureus* | Q6TYQ8:AAQ95777 | 10 | 387 |
| *Aotus trivirgatus* | Q6TYQ9:AAQ95776 | 10 | 383 |
| *Saguinus oedipus* | Q6TYR0:AAQ95775 | 10 | 391 |
| *Ateles geoffroyi* | Q6TYR1:AAQ95774 | 10 | 385 |
| *Lagothrix lagotricha* | Q6TYR2:AAQ95773 | 10 | 386 |
| *Homo sapiens* | ENSG00000175294 | 49 | 780 |
| **PH-20 (Taxa 11)** | | | |
| Cavia porcellus | ENSCPOG00000001716 | 49 | 507 |
| *Homo sapiens* | ENST00000340011 | 49 | 511 |
| Loxodonta africana | **ENSLAFG00000004093** | 49 | 483 |
| Macaca mulatta | ENSMMUT00000008023 | 49 | 512 |
| Oryctolagus cuniculus | ENSOCUT00000011444 | 49 | 511 |
| Ochotona princes | **ENSOPRG00000003627** | 49 | 492 |
| Pan troglodytes | **ENSPTRG00000019643** | 49 | 511 |
| Pongo pygmaeus | **ENSPPYG00000017961** | 49 | 509 |
| Tupaia belangeri | **ENSTBEG00000011237** | 49 | 343 |
| Ornithorhynchus anatinus | ENSOANT00000000657 | 49 | 513 |
| Monodelphis domestica | **ENSMODG00000015368** | 49 | 487 |
| **Semg2 (Taxa 12)** | | | |
| *Homo sapiens* | ENST00000372769 | 49 | 511 |
| Macaca mulatto | ENSMMUG00000017560 | 49 | 706 |
| Pan troglodytes | Q5U7N4: ABO52928 | 25 | 407 |
| Pongo pygmaeus | ENSPPYT00000012826 | 49 | 581 |
| *Colobus guereza* | Q5U7M8: AAV51950 | 17 | 582 |
| *Gorilla gorilla* | Q5U7N3:AAV51945 | 18 | 474 |
| *Hylobates klossi* | Q6X2M3:AAP86629 | 18 | 522 |
| *Hylobates lar* | Q5U7N1:AAV51947 | 18 | 582 |
| *Macaca fascicularis* | Q5U7N0: AAV51948 | 17 | 582 |
| *Macaca nemestrina* | Q5U7M9:AAV51949 | 17 | 582 |
| *Pongo abelii* | P0C7A4: ABO52991 | 1 | 581 |
| **ZP2 (Taxa 18)** | | | |
| Homo sapiens | **ENSG00000103310** | 49 | 745 |
| Bos taurus | **ENSBTAG00000011504** | 49 | 713 |
| Canis familiaris | **ENSCAFG00000017814** | 49 | 742 |
| Equus caballus | **ENSECAG00000019961** | 49 | 744 |
| Felis catus | **ENSFCAG00000015165** | 49 | 640 |
| Loxodonta africana | **ENSLAFG00000015577** | 49 | 740 |
| Macaca mulatto | **ENSMMUG00000016759** | 49 | 745 |
| Mus musculus | **ENSMUSG00000030911** | 49 | 713 |
| Ochotona princeps | **ENSOPRG00000011530** | 49 | 736 |
| Oryctolagus cuniculus | **ENSOCUG00000012909** | 49 | 742 |
| Otolemur garnettii | **ENSOGAG00000011155** | 49 | 724 |
| Pan troglodytes | **ENSPTRG00000007856** | 49 | 739 |
| Pongo pygmaeus | **ENSPPYG00000007168** | 49 | 637 |
| Sorex araneus | **ENSSARG00000003743** | 49 | 674 |
| Spermophilus tridecemlineatus | **ENSSTOG00000007211** | 49 | 735 |
| Tupaia belangeri | **ENSTBEG00000014353** | 49 | 694 |
| Callithrix jacchus | P79160: CAA71740 | 30 | 747 |
| Rattus rattus | O54767: BAA24487 | 44 | 695 |
| **ZP3 (Taxa13)** | | | |
| Homo sapiens | ENST00000394857 | 49 | 424 |
| Bos taurus | **ENSBTAG00000014834** | 49 | 421 |
| Equus caballus | **ENSECAG00000008731** | 49 | 426 |
| Felis catus | **ENSFCAG00000013474** | 49 | 316 |
| Loxodonta africana | **ENSLAFG00000012464** | 49 | 226 |
| Macaca mulatta | ENSMMUT00000005152 | 49 | 371 |
| Pan troglodytes | ENSPTRT00000035756 | 49 | 373 |
| Tupaia belangeri | **ENSTBEG00000011784** | 49 | 370 |
| Canis familiaris | **ENSCAFG00000013648** | 49 | 426 |
| [*Sus scrofa*](http://www.expasy.org/cgi-bin/get-entries?OC=Sus scrofa (Pig)) | P42098 NP_999058 | 54 |  |
| Rattus rattus | **P97708:NM_053762** | 53 | 424 |
| [*Callithrix sp*](http://www.expasy.org/cgi-bin/get-entries?OC=Callithrix sp. (Marmoset)) | P53786: AAB31866 | 47 | 424 |
| Macaca radiata | P53785: CAA57961 | 43 | 424 |
| **PRKAR2A (Taxa17)** | | | |
| Homo sapiens | ENST00000265563 | 49 | 404 |
| Bos taurus | ENSBTAT00000018886 | 49 | 401 |
| Canis familiaris | ENSCAFT00000019230 | 49 | 403 |
| Cavia porcellus | ENSCPOT00000001232 | 49 | 350 |
| Macaca mulatta | ENSMMUT00000000115 | 49 | 403 |
| Mus musculus | ENSMUST00000035220 | 49 | 402 |
| Pan troglodytes | ENSPTRT00000027889 | 49 | 396 |
| Pongo pygmaeus | ENSPPYT00000016160 | 49 | 382 |
| Rattus norvegicus | **ENSRNOG00000020284** | 49 | 401 |
| Tupaia belangeri | **ENSTBEG00000017443** | 49 | 401 |
| Erinaceus europaeus | **ENSEEUG00000003335** | 49 | 316 |
| Felis catus | **ENSFCAG00000008392** | 49 | 269 |
| Loxodonta africana | **ENSLAFG00000003894** | 49 | 310 |
| Ochotona princeps | **ENSOPRG00000011607** | 49 | 376 |
| Oryctolagus cuniculus | **ENSOCUG00000012426** | 49 | 304 |
| Otolemur garnettii | **ENSOGAG00000006822** | 49 | 316 |
| Sorex araneus | **ENSSARG00000008087** | 49 | 304 |
| **Col1a1(Taxa 10)** | | | |
| Homo sapiens | **ENSG00000108821** | 49 | 1464 |
| Bos taurus | PO2453: NP_001029211 | 72 | 1463 |
| Canis familiaris | **ENSCAFG00000017018** | 49 | 1476 |
| Felis catus | **ENSFCAG00000004564** | 49 | 1154 |
| Mus musculus | P11087 :NP_031768 | 94 | 1225 |
| Ochotona princeps | **ENSOPRG00000013172** | 49 | 1450 |
| Pan troglodytes | **ENSPTRG00000009393** | 49 | 1464 |
| Rattus rattus | ENSRNOT00000005311 | 49 | 1453 |
| Pongo pygmaeus | **ENSPPYG00000008937** | 49 | 1463 |
| Monodelphis domestica | ENSMODT00000015251 | 49 | 1458 |
| **Porimin (Taxa 10)** | | | |
| Homo sapiens | ENST00000361236 | 49 | 189 |
| Equus caballus | ENSECAT00000012074 | 49 | 117 |
| Erinaceus europaeus | **ENSEEUG00000012089** | 49 | 184 |
| Macaca mulatta | **ENSMMUG00000003403** | 49 | 188 |
| Mus musculus | **ENSMUSG00000050912** | 49 | 195 |
| Pan troglodytes | **ENSPTRG00000022738** | 49 | 189 |
| Pongo pygmaeus | **ENSPPYG00000003809** | 49 | 157 |
| Rattus norvegicus | **ENSRNOG00000010584** | 49 | 187 |
| Sorex araneus | **ENSSARG00000006647** | 49 | 130 |
| Monodelphis domestica | ENSMODT00000000748 | 49 | 155 |
| **SP-56(Taxa 14)** | | | |
| Homo sapiens | ENST00000368867 | 49 | 472 |
| Bos taurus | **ENSBTAG00000008091** | 49 | 472 |
| Canis familiaris | **ENSCAFG00000012675** | 49 | 472 |
| Equus caballus | ENSECAT00000019402 | 49 | 471 |
| Felis catus | **ENSFCAG00000001859** | 49 | 478 |
| Loxodonta africana | ENSLAFT00000011764 | 49 | 477 |
| Ochotona princes | ENSOPRT00000007267 | 49 | 458 |
| Oryctolagus cuniculus | **ENSOCUG00000003371** | 49 | 413 |
| Otolemur garnettii | **ENSOGAG00000008124** | 49 | 349 |
| Pan troglodytes | ENSPTRT00000002391 | 49 | 472 |
| Pongo pygmaeus | **ENSPPYG00000000866** | 49 | 489 |
| Rattus norvegicus | **ENSRNOG00000020992** | 49 | 473 |
| Sorex araneus | **ENSSARG00000000216** | 49 | 327 |
| Tupaia belangeri | **ENSTBEG00000002411** | 49 | 434 |
