# Supplementary material for: Positive selection neighboring functionally essential sites and disease-implicated regions of mammalian reproductive proteins
Source: BMC Evol Biol. 2010 Feb 11;10:39. doi: 10.1186/1471-2148-10-39 (PMC2830953; doi:10.1186/1471-2148-10-39)
Supplement: Additional file 8 — Additional Table 8 - Parameters for Phylogeny Reconstruction per gene. The parameters used to reconstruct each gene tree in MrBayes are shown. The model of rate heterogeneity for each gene is shown, along with the number of generations required, and the number of markov chains (these values vary based on the size of the dataset). [file 1471-2148-10-39-S8.DOC]

**Additional Table 8: Parameters for Phylogeny Reconstruction per gene.**

| **Gene** | **Rate Heterogeniety** | **Number of Generations** | **Markov Chains** |
| --- | --- | --- | --- |
| **Adam2** | discrete gamma | 400000 | 64 |
| **Catsper1 Exon1** | invariable sites and discrete gamma | 500000 | 8 |
| **Catsper1 Mammals** | discrete gamma | 500000 | 64 |
| **Col1a1** | discrete gamma | 500000 | 64 |
| **Ph20** | discrete gamma | 500000 | 64 |
| **Porimin** | discrete gamma | 500000 | 64 |
| **Prkar2a** | invariable sites and discrete gamma | 550000 | 64 |
| **Semg2** | discrete gamma | 500000 | 64 |
| **Sp56** | invariable sites and discrete gamma | 600000 | 30 |
| **Zp2** | discrete gamma | 500000 | 64 |
| **Zp3** | discrete gamma | 500000 | 64 |
