# Supplementary material for: Positive selection neighboring functionally essential sites and disease-implicated regions of mammalian reproductive proteins
Source: BMC Evol Biol. 2010 Feb 11;10:39. doi: 10.1186/1471-2148-10-39 (PMC2830953; doi:10.1186/1471-2148-10-39)
Supplement: Additional file 9 — Additional Table 9 - Likelihood ratio tests (LRTs) performed using all evolutionary models used in selection analysis. Details on all likelihood ratio tests performed in the analysis. The models are denoted by their abbreviated names, Model A1 is denoted as Model A null throughout the manuscript. The number of degrees of freedom (df) are shown, this is relevant for the chi-squared test for significance, the critical values in each instance are given in the final column. [file 1471-2148-10-39-S9.DOC]

**Additional Table_9: Likelihood ratio tests (LRTs) performed using all evolutionary models used in selection analysis**

| **Comparison** | ***df*** | ***Δl*** | **Critical χ^2^ values** |
| --- | --- | --- | --- |
| M0 v M3k2 | 2 | X2 | ≥ 5.99 |
| M3k2 v M3k3 | - | X1 | ≥ 1.00 |
| M1 v M2 | 2 | X2 | ≥ 5.99 |
| M7 v M8 | 2 | X2 | ≥ 5.99 |
| M8 v M8a* | 1 | X2 | ≥ 2.71 (@5%)  ≥ 5.41 (@1%) |
| M1 v Model A | 2 | X2 | ≥ 5.99 |
| Model A v Model A1(denoted as model a null throughout the manuscript) | 1 | X2 | ≥ 3.84 (@5%) |
| M3k2 v Model B | 2 | X2 | ≥ 5.99 |

* One degree of freedom for the chi square and comparison using a 50:50 mixture of point mass 0 and χ^2^, so the critical χ^2^ values are 2.71 at **5%** and 5.41 at **1%**, and not 10% and 2% respectively.
